# From Diagnosis to Disease Staging: Multisite Validation of Cerebrospinal Fluid Molecular Tests in Multiple Sclerosis

**DOI:** 10.1002/ana.78047

**Published:** 2025-10-03

**Authors:** Laura Ghezzi, Peter Kosa, Mark Greenwood, Enrique Alvarez, C. L. Freedman, Anne H. Cross, Francesca Pace, Mark S. Freedman, Joanna Kocot, Laura Piccio, Bibiana Bielekova

**Affiliations:** ^1^ Department of Biomedical, Surgical and Dental Sciences University of Milan Milan Italy; ^2^ IRCCS Foundation Ca'Granda Ospedale Maggiore Policlinico Milan Italy; ^3^ Neuroimmunological Diseases Section, Laboratory of Clinical Immunology and Microbiology, National Institute of Allergy and Infectious Diseases, National Institutes of Health Bethesda MD USA; ^4^ Department of Mathematical Sciences Montana State University Bozeman MT USA; ^5^ Department of Neurology, Rocky Mountain Multiple Sclerosis Center at the University of Colorado Aurora CO USA; ^6^ University of Ottawa Department of Medicine and the Ottawa Hospital Research Institute Ottawa ON Canada; ^7^ Department of Neurology Washington University School of Medicine St Louis MI USA; ^8^ Charles Perkins Centre and Brain and Mind Centre, Neuroscience, School of Medical Sciences, Faculty of Medicine and Health University of Sydney Sydney New South Wales Australia

## Abstract

**Objective:**

The growing demand for personalized treatment in multiple sclerosis (MS) highlights the need for more precise biomarkers that can outperform magnetic resonance imaging and clinical assessment in patient stratification. Advances in multiplex proteomic technologies suggest that cerebrospinal fluid (CSF) analysis at MS onset may not only improve diagnostic accuracy, but also offer prognostic and staging information, as well as insight into molecular therapeutic targets.

**Methods:**

This multicenter study retrospectively analyzed cryopreserved CSF samples from 160 individuals undergoing diagnostic evaluation for possible neuroimmunological disorder, and among these, followed a cohort of 96 people with confirmed MS for at least 3 years. The goal was to externally validate previously published CSF‐based diagnostic and prognostic classifiers.

**Results:**

Upon unblinding, the CSF‐based molecular diagnostic test distinguished 96 people with confirmed MS from 30 individuals with other inflammatory neurological diseases, and 34 individuals with non‐inflammatory neurological diseases, achieving an area under the receiver operating characteristic curve of 0.94 (*p* = 4.7 × 10^−21^). The test also differentiated 65 individuals with relapsing–remitting MS from 31 individuals with progressive MS, with an area under the receiver operating characteristic curve of 0.76 (*p* = 1.4 × 10^−5^). The prognostic classifier predicted prospectively measured Expanded Disability Status Scale scores at follow up (rho = 0.43, *p* = 2.54 × 10^−5^).

**Interpretation:**

This multicenter external validation study demonstrates that CSF‐based molecular tests can robustly distinguish MS from other neurological conditions, stratify MS subtypes, and predict future disability progression in real‐world settings. These results lay the groundwork for development of next‐generation molecular tools to personalize care in MS. ANN NEUROL 2026;99:328–340

Clinical diagnoses of neurological disorders carry an estimated error rate of 20 to 40% when compared with pathological gold standards.^1^, ^2^ A similar degree of diagnostic uncertainty exists for multiple sclerosis (MS): magnetic resonance imaging (MRI)‐based diagnostic accuracy yields an area under the receiver operating characteristic curve (AUROC) of 0.65–0.70.^3^ Misdiagnosis can delay necessary treatment for people with MS (pwMS), while exposing others to ineffective or unnecessary therapies. Consequently, MS diagnostic criteria continue to evolve.^4^

To improve accuracy, current MS criteria incorporate cerebrospinal fluid (CSF) biomarkers of intrathecal humoral immune activation—specifically, the immunoglobulin G (IgG) index and oligoclonal bands (OCBs).^5^ However, these biomarkers were established decades ago and lack specificity for MS. Recent advances in high‐throughput multiplex proteomics and mass spectrometry now allow relative and absolute quantification of thousands of proteins in human CSF. These innovations open the door to molecular diagnostic tests with higher accuracy, prognostic value, and potential to guide therapeutic decisions.

Using machine learning, we previously developed and internally validated CSF biomarker‐based classifiers with clinical utility for MS. These include:

- A molecular diagnostic test that distinguishes MS from different inflammatory and non‐inflammatory central nervous system diseases who presented for diagnostic work‐up of neuroimmunological disorder, with an AUROC of 0.98 in an independent cohort.^6^
- A classifier that differentiates relapsing–remitting MS (RRMS) from progressive MS (progMS), with an AUROC of 0.91.^6^ We observed that the 2 progressive MS subtypes—primary progressive (PPMS) and secondary progressive (SPMS)—show overlapping intrathecal proteomic profiles that are indistinguishable using current assays.^6^
- More recently, we identified a CSF‐based prognostic test that predicts future treatment‐adjusted rates of disability progression, validated in an independent cohort.^7^

Although these findings underscore the potential of CSF biomarker‐based models to improve MS diagnosis and management, they were all derived from a single research center. Therefore, external validation in real‐world clinical settings is essential to confirm their robustness, generalizability, and potential for regulatory approval. The multicenter SPINal Fluid COnsortiuM for MS (SPINCOMS) was established to evaluate the clinical performance of these CSF‐based molecular tests in a diverse cohort of pwMS and individuals undergoing diagnostic workup for central nervous system conditions that can mimic MS clinically or radiologically.

## Methods

### Study Design

**FIGURE 1:**
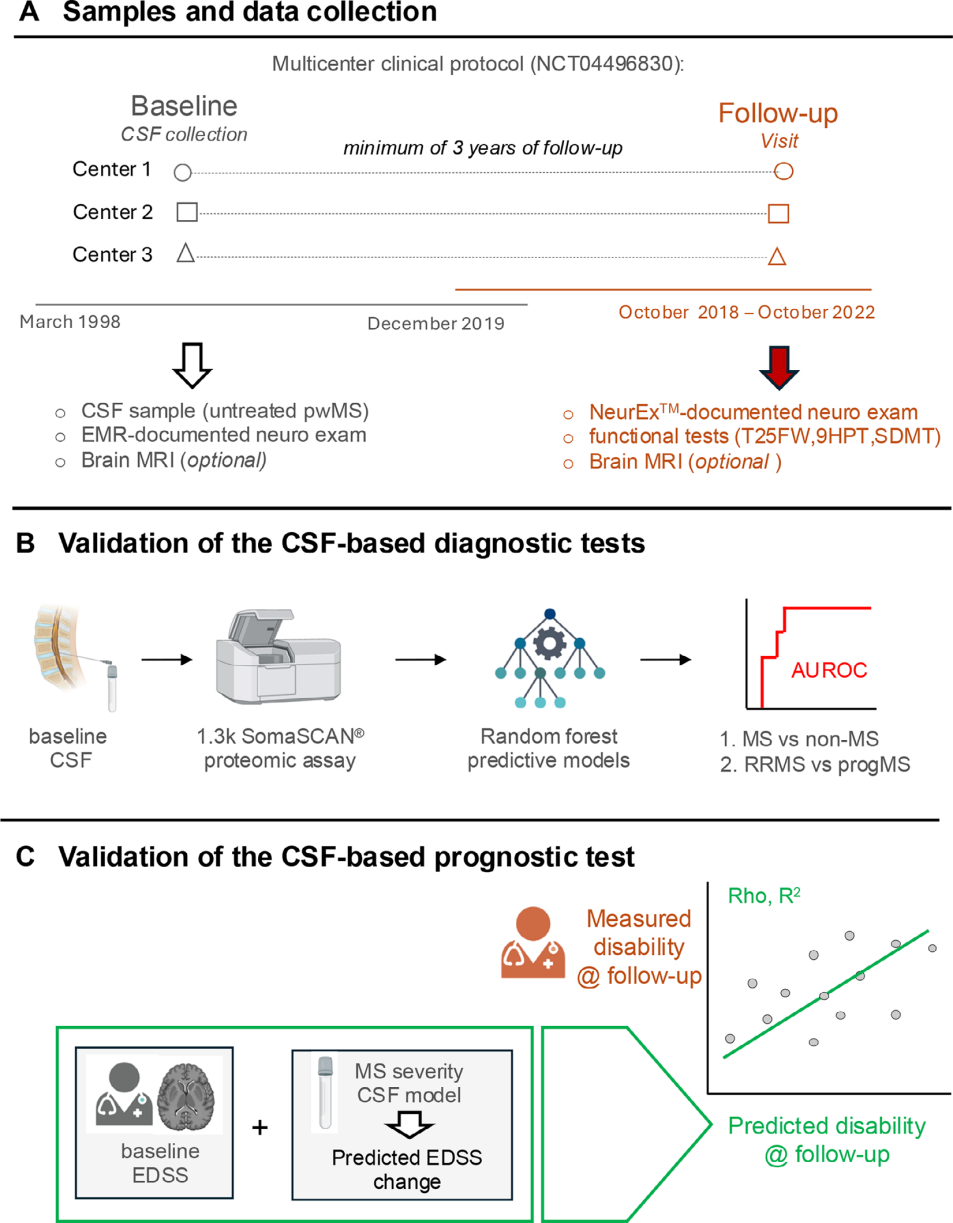
Study design. (A) This study took advantage of prospectively (between March 1998 and December 2019) collected and cryopreserved cerebrospinal fluid (CSF) samples from 3 academic centers under a multicenter clinical protocol (NTC04496830; Washington University in St. Louis; University of Ottawa; University of Colorado). Baseline CSF collected in the untreated state was accompanied with neurological examinations documented in electronic medical records (EMR), and with optional brain and spinal cord magnetic resonance imaging (MRI) performed at time of collection. Follow‐up visits occurred between October 2018 and October 2022, and consisted of neurological exam documented in NeurEx™ app, functional tests, and optional brain MRI (which was performed as part of their clinical work‐up). (B) Baseline CSF was analyzed using SOMAScan assay®, and the generated proteomic information was plugged into previously described random forest models to predict probability of multiple sclerosis (MS) diagnosis and progressive MS diagnosis. The performance of these model predictions was evaluated by calculating the area under the receiver operating characteristic curve (AUROC). (C) SOMAScan‐generated CSF proteomic data was also used to predict future accumulation of disability from the baseline CSF. The predicted disability accumulation between baseline and follow‐up visits was added to baseline disability levels (either generated based on documented neuro examinations or predicted from baseline MRI), and the predicted disability at follow‐up was compared to measured disability at follow‐up determined by a neurologist. EDSS = Expanded Disability Status Scale; T25FW=times 25‐Foot Walk; 9HPT=Nine‐Hole Peg Test; SDMT= symbol digit modalities test; progMS= progressive MS; AUROC= area under the receiver operating characteristic curve. [Color figure can be viewed at www.annalsofneurology.org]

This multicenter study, conducted in the USA and Canada, involved a retrospective analysis of cryopreserved CSF samples collected from individuals undergoing diagnostic evaluation for possible neuroimmunological disorders, combined with prospective clinical follow up of those ultimately diagnosed with MS. To avoid introducing bias in favor of CSF‐based biomarkers, we selected patients solely based on their final clinical diagnosis, without considering routine CSF tests (such as oligoclonal bands or IgG index), as these were already available to the clinicians and thus inherently reflected in the clinical diagnosis. The study was approved by the Washington University in St. Louis Institutional Review Board (HRPO #202004205) and registered on ClinicalTrials.gov under the SPINCOMS multicenter protocol (NCT04496830). All participants signed an informed consent. The overall study design is illustrated in Figure 1.

Based on prior data,^6^, ^7^ we estimated that a sample size of 100 participants per classifier would provide >90% power to independently validate the clinical utility of each CSF‐based molecular test. For the MS diagnostic classifier, we aimed to recruit 50 individuals with confirmed MS and 50 with alternative diagnoses. For the RRMS versus progMS (PPMS + SPMS) classifier, we aimed to enroll 50 participants per group. We intended to follow the combined MS cohort (*n* = 100) prospectively to validate the CSF‐based prognostic model.^7^ The numbers of recruited participants and the data collected were negatively impacted by the COVID‐19 pandemic.

Demographic and clinical data at the time of CSF collection were extracted from medical records and entered into a REDCap database under anonymized codes. Neurological examinations were retrospectively transcribed into NeurEx,^8^ an iPad‐based app that automates disability scale calculations, such as the Expanded Disability Status Scale (EDSS)^9^ or Scripps Neurological Rating Scale^10^. Although NeurEx standardizes scoring, it cannot correct for variation in examination performance or documentation. As baseline examinations were performed by different clinicians and recorded as free text rather than structured case report forms, medical record‐derived EDSS values were expected to be imprecise.

SPINCOMS Centers prioritized enrolling pwMS with brain and spinal cord MRIs performed within 3 months of diagnostic lumbar puncture (“baseline MRI”) and obtaining “follow‐up” MRIs as clinically indicated. To offset limitations in baseline EDSS precision, we incorporated machine learning‐based MRI models to predict disability (see Methods section).

Participant selection and retrieval of cryopreserved CSF samples occurred at 3 non‐National Institutes of Health (NIH) SPINCOMS academic centers (Washington University in St. Louis, University of Ottawa, and University of Colorado; Fig 1), all blinded to CSF and MRI model outputs. The NIH site generated CSF biomarker data (MS probability, progMS probability, and disease severity) and MRI‐based EDSS predictions while blinded to clinical information. Statistical analyses were performed independently by a biostatistician (M.G.) not involved in data generation.

The control groups of people with other inflammatory or non‐inflammatory diseases, used in the diagnostic classifier analysis, contributed only retrospective demographic data and were not prospectively followed (Table 1).

**TABLE 1:** Demographic and Clinical Characteristics of Study Participants

	RRMS			ProgMS		OIND		NIND
	CSF	Follow‐up visit in person/telehealth		CSF	Follow‐up visit in person/telehealth	CSF		CSF
Total (*n*)	65	44/21		31	26/5	**30**		**34**
Center 1 (Colorado)	12	10/2		7	7/0	6		11
Center 2 (Ottawa)	6	6/0		18	18/0	5		4
Center 3 (Wash U)	47	28/19		6	1/5	19		19
Age, yr (range)	38.1 ± 10.7 (18–67)	47.2 ± 11.5		51.0 ± 10.5 (36–81)	60.1 ± 9.1	41.3 ± 13.2 (17–62)		39.7 ± 12.5 (18–69)
Sex (F/M)	44/21			17/14		20/10		28/6
Disease duration at lumbar puncture (yr)	4.19 ± 5.89			6.06 ± 5.0		NA		NA
Disease duration at follow‐up visit (yr)		13.20 ± 7.96			16.58 ± 7.98	NA		NA
Median EDSS, IQR (range)^a^	2.0; 1.5 (0.0–7.0)	2.25, 2.5 (0.0–7.5)		3.0; 1.0 (1.0–6.0)	6.0, 3.0 (1.5–8.0)	NA		NA
DMT (Y/N)	0/65	50/15		0/31	15/16	NA		NA
Ocrelizumab		12			12			
Rituximab		3			3			
Teriflunomide		3						
Interferon‐beta 1a		4						
Interferon beta 1b		1						
Copaxone		8						
Fingolimod		3						
Alemtuzumab		1						
Siponimod		1						
Dimethyl fumarate		5						
Natalizumab		6						
Relapse rate		0.14 ± 0.28			0	NA		NA
CSF results						NA		NA
OCB (+/−)	49/12			23/6		5/15		0/12
IgG index altered (Y/N)	36/25			14/14		5/16		0/12
Follow‐up period, yr (mean ± SD)		8.18 ± 9.83			10.97 ± 5.85	NA		NA
MRI, *n* (brain/upper spinal cord)	39	15		15	6	NA		NA
Clinical testing (Y/N)^b^		38/27			22/10	NA		NA
SDMT		50 ± 13			34 ± 14	NA		NA
T25FW (s)		5.59 ± 2.83			10.13 ± 4.87	NA		NA
9HPT, D/ND (s)		23 ± 8.41	23.7 ± 6.81		28.24 ± 9.74	31.18 ± 9.27	NA	NA

Sample sizes changed for some variables based on available information – see supplemental information for additional details. Disease durations, disease‐modifying therapies, relapse rate, and clinical testing results are reported in the table, but were not included in the analyses.

^a^ Retrospectively calculated from the clinical chart at cerebrospinal fluid collection, while obtained from neurological examination at follow‐up visit; 7 people with multiple sclerosis lacked Expanded Disability Status Scale scores at baseline and 5 at follow‐up visit.

^b^ Complete motor function data (9‐Hole Peg Test and Timed 25‐Foot Walk) were available for 59 participants, and Symbol Digit Modalities Test was completed by 60 participants.

25FWT = 25‐Foot Walk Test; 9HPT = 9‐Hole Peg Test; DMT = disease‐modifying therapies; D/ND = dominant/non‐dominant hand; EDSS = Expanded Disability Status Scale; NA = not applicable; NIND = non‐inflammatory neurological diseases; OIND = other inflammatory neurological diseases; ProgMS = progressive multiple sclerosis; RRMS = relapsing–remitting multiple sclerosis; SDMT = Symbol Digit Modalities Test.

### CSF Collection and Processing

CSF was collected at each of the 3 SPINCOMS centers between March 1988 and December 2019. In all 3 centers, CSF was centrifuged at 300 *g* for 10 minutes at 4°C, aliquoted, and frozen at −80°C for future use.

### Clinical Assessments at Follow‐Up Visits

At follow‐up visits (performed between October 2018 and October 2022), pwMS underwent clinical evaluation including EDSS scoring, manual dexterity testing (9‐Hole Peg Test [9HPT]), ambulation assessment (Timed 25‐Foot Walk [T25FW]), and cognitive screening (Symbol Digit Modalities Test [SDMT]).^11^ 9HPT and T25FW were available for 59 participants, and SDMT was completed by 60 participants. One participant completed only SDMT due to physical limitations. Of the 37 participants missing 9HPT/T25FW, 26 missed in‐person visits (because of restrictions related to the COVID‐19 pandemic), 4 declined testing, and 7 were physically unable to perform the tasks.

### MRI Details

Baseline MRIs (brain and upper cervical spine) were available for 54 participants, and follow‐up MRIs for 21 participants. Scans were acquired on 1.5 or 3T clinical systems, and included T2‐weighted and/or fluid‐attenuated inversion recovery, and T1‐weighted sequences that may include magnetization prepared rapid gradient echo, echo planar imaging, or volumetric true fast imaging. MRIs were rated semiquantitatively by a single blinded rater (B.B.) using a validated protocol shown to be consistent across 1.5 and 3T systems,^12^ and entered into FileMaker‐based case report forms to automatically compute Combinatorial MRI Scale (COMRIS) disability predictions. Details of the COMRISv2 algorithm used to estimate EDSS are published.^13^

### CSF Protein Quantification (SOMAscan)

Relative concentrations of 1,305 CSF proteins were measured using the SOMAscan 1.3 k assay, which utilizes Slow Off‐rate Modified Aptamers (SOMAmers) to bind target proteins. Raw relative fluorescent units were normalized within and across assay plates, as described.^7^

### CSF Soluble CD27 (sCD27) and Neurofilament Light Chain Enzyme‐Linked Immunosorbent Assay

sCD27, a previously validated MS biomarker, was quantified by homebrew electrochemiluminescence enzyme‐linked immunosorbent assay on the Meso Scale Discovery platform with the MSD R‐Plex Human sCD27 Antibody Set (Meso Scale Discovery, Rockville, MD, USA; #F213D‐3), as described.^14^, ^15^ Neurofilament light chain was measured using UmanDiagnostics ELISA kit (Umeå, Sweden; #10‐7,002 RUO), following the manufacturer's instructions. All samples were diluted 1:2 and analyzed blindly.

### Validation of SOMAscan‐Based Diagnostic Predictors

Due to the discontinuation of the original 1.1 k SOMAscan platform used in Barbour et al,^6^ we reconstructed analogous diagnostic and prognostic models using the 1.3 k platform, adjusting for age and sex using published healthy volunteer data.^6^, ^7^

The original molecular diagnostic test of MS^6^ was constructed using 22 protein ratios derived from 24 unique CSF proteins quantified with the SOMAscan 1.1 k platform. One of these SOMAmers, targeting IgG heavy chain, was excluded from the 1.3 k assay by the manufacturer due to cross‐reactivity (with mouse IgG‐Fc receptor). As this SOMAmer was involved in 2 of the original 22 ratios (ie, IgG heavy chain/DSG2 and IgG heavy chain/IgM), its absence in the 1.3 k panel reduced the measurable set to 20 protein ratios based on 21 unique CSF proteins: TNFRSF17, CD48, PRTN3, PLA2G7, PDCD1LG2, CCL7, SLAMF6, TLR4/LY96 complex, CSF3, CDKN1B, TNFRSF6B, TNC, CRK, PGK1, MAPK14, FLT4, F9, CXCL13, TNFRSF4, DCTPP1, and MMP7.

The published progMS classifier^6^ consisted of 21 protein ratios derived from 24 unique CSF proteins: EDA2R, SELL, CFD, SERPING1, ICOSLG, LTA/LTB, IL22, INHBA, GP6, EDAR, PRTN3, LILRB2, STX1A, JAM3, EPHA5, GZMA, NTRK3, IL10, CLEC1B, TYRO3, BOC, ETHE1, UNC5C, and RGMA.

The adaptation of these classifiers to the 1.3 k platform was performed using samples from the NIH (not the SPINCOMS) cohort.^7^

Random forest models^6^ generated probabilities (0–1) for MS and progMS diagnosis, dichotomized at 0.5. Predicted classifications were compared with clinical diagnoses using AUROC analysis (*pROC* R package^16^; the R Foundation for Statistical Computing, Austria, Vienna; Fig 1B).

### Validation of SOMAscan‐Based Prognostic Model

The CSF‐based prognostic model^7^ estimates the MS Disease Severity Scale (MS‐DSS^17^) using 57 SOMAmer ratios (and 75 unique proteins) measured by 1.3 k SOMAscan. MS‐DSS is a gradient boosting machine model that integrates 13 clinical and demographic predictors, and has been shown to correlate with future disability progression rates in an independent validation cohort (*r* = 0.54, *p* = 1.56e−06^17^). Disability progression was originally quantified using the Combinatorial Weight‐Adjusted Disability Scale (CombiWISE^18^), a machine learning‐derived linear scale ranging from 0 to 100 that integrates EDSS, Scripps Neurological Rating Scale, T25FW, and 9HPT. Although CombiWISE correlates strongly with EDSS (Spearman's rho = 0.98, *p* < 0.0001), its granularity enables reliable quantification of progression slopes over short (1–5 years) intervals,^18^ which is not possible using EDSS.

Because the SPINCOMS cohort lacked the full set of clinical variables needed to compute MS‐DSS and CombiWISE, we could not perform a direct validation of the CSF‐based prognostic model. Instead, we devised an indirect validation by estimating disability at the follow‐up timepoint. Specifically, we used the CSF‐predicted MS‐DSS to derive a CombiWISE slope, which was then converted to an EDSS change using a linear regression model derived from an external (NIH) cohort.^7^ This predicted EDSS change was added to 2 separate baseline EDSS estimates: (1) the NeurEx™‐derived EDSS from transcribed neurological examinations, and (2) the COMRISv2‐predicted EDSS from baseline MRI.^13^ The resulting predicted follow‐up EDSS values were then compared with the actual EDSS scores measured at the in‐person follow‐up visit (Fig 1C).

### Statistical Analyses

All analysis results were generated using R version 4.3.3 (The R Foundation for Statistical Computing).^19^ All graphs were made using base R or ggplot2.^20^ Multiple linear regression models were used for models of quantitative variables. AUROC confidence intervals were generated using bootstrapping and *p* values using the method in Mason and Graham (2002).^21^ All raw data (SOMAscan relative fluorescent units, proteomic and predictive models, NFL, and sCD27 concentrations) and analysis codes are available as Supporting Information.

## Results

### CSF‐Based Molecular Diagnostic Test Accurately Differentiates MS from Other Neurological Diseases

The molecular diagnostic test for MS was validated in a blinded analysis using relative concentrations of 21 CSF proteins measured by the SOMAscan 1.3 k assay in 160 individuals: 96 with clinically confirmed MS and 64 non‐MS controls, including individuals with other inflammatory or non‐inflammatory neurological diseases. Many other inflammatory neurological diseases cases presented with clinical or MRI findings that can mimic MS. The classifier outputs a continuous probability score (0–1), representing the likelihood of an MS diagnosis.

**FIGURE 2:**
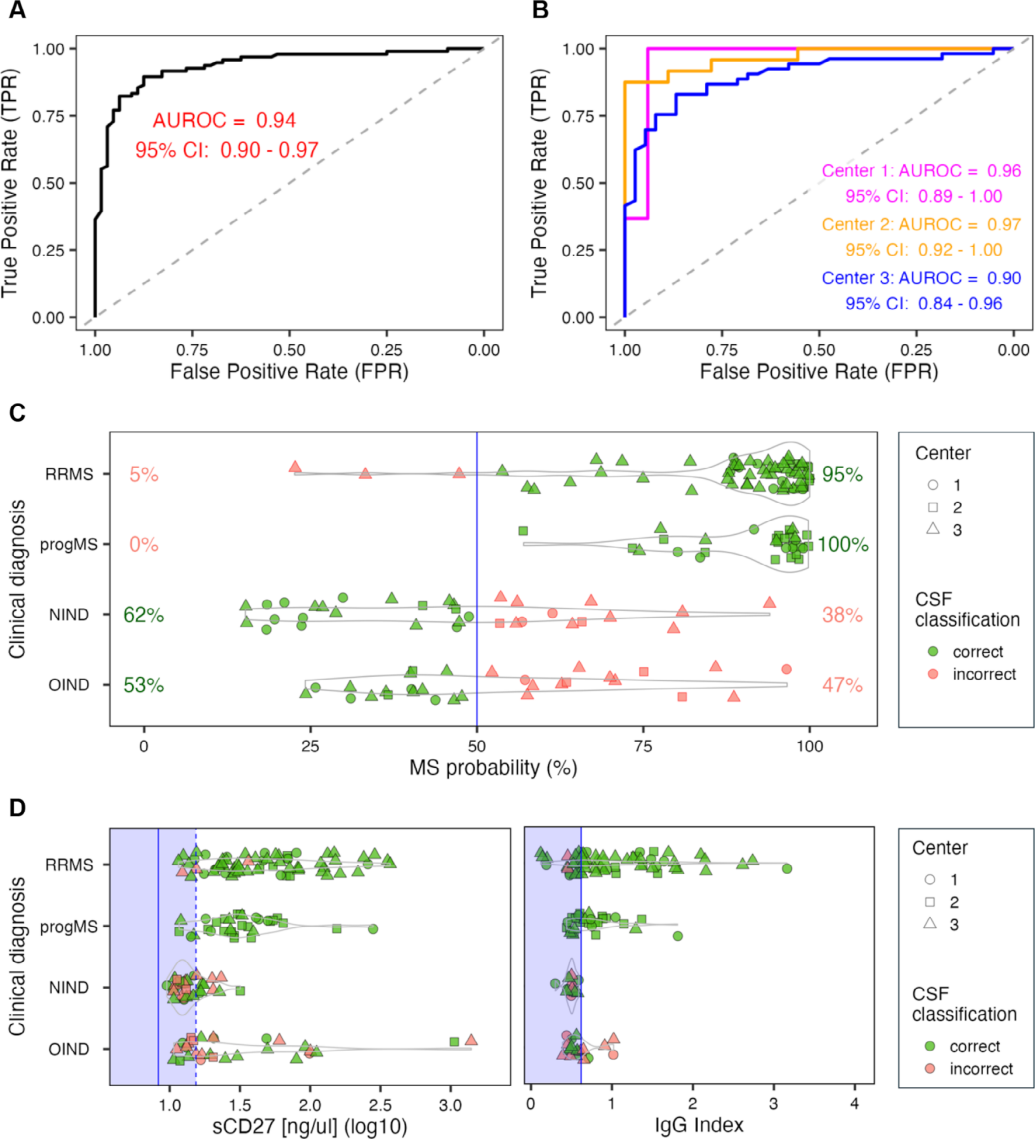
Multiple sclerosis (MS) versus non‐MS classifier. (A) area under the receiver operating characteristic curve (AUROC) was generated by comparing predicted and clinician‐generated diagnosis of MS versus non‐MS controls, with 95% confidence interval (CI). (B) AUROC of MS versus non‐MS classifier separated into the 3 academic centers that contributed cerebrospinal fluid (CSF) samples for the study. (C) MS probability (*x*‐axis) versus clinical diagnosis (*y*‐axis) of relapsing–remitting MS (RRMS), progressive MS (progMS), non‐inflammatory neurological diseases (NIND), and other inflammatory neurological diseases (OIND). The blue vertical line marks 50% probability cutoff. The percentages show proportions of correctly (green) and incorrectly (pink) classified samples within each clinical diagnostic group. (D) Distribution of CSF levels of soluble sCD27 measured by enzyme‐linked immunosorbent assay (plot on the left) and available immunoglobulin G (IgG) index values among the 4 clinical diagnostic groups. The solid blue vertical line represents healthy donor average in the Neuroimmunological Diseases Section's (NDS) healthy cohort (for sCD27) and normal cutoff value (0.62). The vertical blue dashed line (plot on the left) represents 2 standard deviations of the NDS healthy cohort. Light blue shaded area represents normal (healthy) values. (C,D) The shape of individual points represents individual centers that contributed the samples, the green color highlights samples that were correctly classified by the molecular diagnostic test, whereas pink points mark misclassified samples. [Color figure can be viewed at www.annalsofneurology.org]

After unblinding, the median predicted probability of MS was 0.95 for pwMS and 0.47 for non‐MS controls. The classifier achieved an AUROC of 0.94 (95% CI 0.90–0.97; *p* = 4.7 × 10^−21^; Fig 2A). Using a predefined probability cutoff of 0.5, the molecular test showed an overall predictive accuracy of 0.81, with sensitivity of 0.97, specificity of 0.58, positive predictive value of 0.78, and negative predictive value of 0.92.

Diagnostic performance varied across SPINCOMS centers, with Center 2 showing the highest and Center 3 the lowest accuracy (Fig 2B). Only 3 of 96 pwMS (3%) were misclassified as non‐MS by the molecular test (Fig 2C). Among these, IgG index and OCB values were available for 2 participants, both of whom had values in the normal (healthy donor) range. Additionally, these same individuals showed normal CSF levels of sCD27, a validated biomarker of intrathecal T‐cell activation (Fig 2D).^14^, ^15^ These findings suggest that the few pwMS cases misclassified by the molecular test lacked the typical biological signature of MS—namely, intrathecal activation of adaptive immunity involving both B and T cells, which is the target of MS treatments.

As a sensitivity analysis to assess whether the SOMAscan‐based MS diagnostic test outperforms traditional CSF biomarkers, we calculated AUROC values for dichotomized (normal/abnormal) IgG index and OCB results in all pwMS and controls with available data (89 pwMS and 32 non‐MS controls). We then compared these with the SOMAscan‐based classifier performance in the same individuals using DeLong's test for statistical comparison. In this cohort, the SOMAscan classifier achieved an AUROC of 0.929, significantly higher than OCB alone (AUROC 0.821, *p* = 0.003), IgG index (AUROC 0.703, *p* = 2.2 × 10^−8^), and the combination of OCB + IgG index (AUROC 0.785, *p* = 0.0009). In conclusion, the CSF‐based molecular diagnostic test reliably distinguished MS from clinically similar disorders with high accuracy and strong statistical significance, supporting its clinical utility in differentiating MS from mimicking conditions.

### CSF‐Based Molecular Classifier of ProgMS Validates its Clinical Utility

**FIGURE 3:**
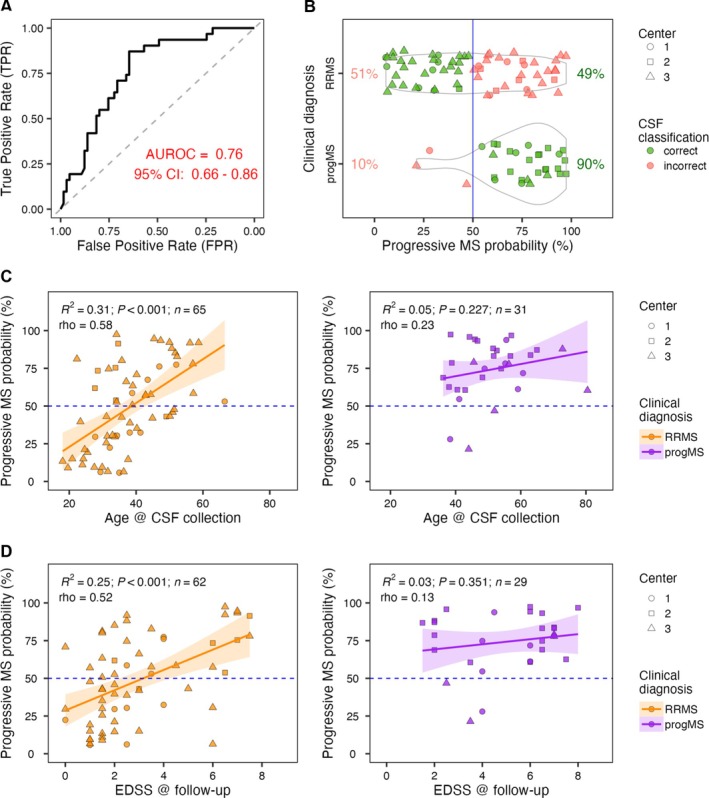
Relapsing–Remitting Multiple Sclerosis (RRMS) versus progressive (progMS) classifier. (A) Area under the receiver operating characteristic curve (AUROC) was generated by comparing predicted and clinician‐generated diagnosis of RRMS versus progressive MS (primary + secondary progressive MS). 95% CI = 95% confidence interval. (B) The progMS probability (*x*‐axis) versus clinical diagnosis (*y*‐axis) of RRMS and progMS. The blue vertical line marks 50% probability cutoff. The percentages show proportions of correctly (green) and incorrectly (pink) classified samples within each clinical diagnostic group. The shape of individual points represents individual centers that contributed the samples, the green color highlights samples that were correctly classified by the molecular diagnostic test, whereas pink points mark misclassified samples. (C) Relationship of progMS diagnostic probability and age at the time of cerebrospinal fluid (CSF) sample collection in clinically‐diagnosed RRMS (left) and progMS (right) individuals. (D) Relationship between progMS diagnostic classifier probability and Expanded Disability Status Scale (EDSS) measured at the follow‐up visit in clinically‐diagnosed RRMS (left) and progMS (right) individuals. (C,D) The shape of individual points represents individual centers that contributed the samples, the orange color highlights RRMS samples, whereas purple points show progMS samples. The orange and purple lines with shaded areas represent linear regression models with 95% confidence intervals for RRMS and progMS samples, respectively. Each panel reports the linear fit's coefficient of determination (*R* ^2^), its *p*‐value (*p*) and sample size (n), with the Spearman correlation coefficient (rho) shown just below. The blue horizontal dashed lines mark 50% probability cutoff. [Color figure can be viewed at www.annalsofneurology.org]

The CSF‐based progMS classifier demonstrated good sensitivity at the time of diagnosis, although with lower specificity compared with the MS diagnostic classifier. The predictive AUROC was 0.76 (95% CI 0.66–0.86; *p* = 1.4 × 10^−5^), with a median predicted probability of 0.78 for individuals with progMS and 0.47 for those with RRMS (Fig 3A,B).

Analysis of misclassified cases revealed two key trends:

1. The classifier tended to assign older RRMS patients to the progMS category, while correctly identifying younger progMS patients as such (Fig 3C).

2. Higher CSF‐predicted probabilities of progMS were associated with greater disability at follow up, as measured by EDSS (Fig 3D).

These simple correlations raised the question of whether the progMS classifier truly captures biological progression or merely reflects age and baseline disability. To address this, we fit a multiple linear regression model with follow‐up EDSS as the outcome, and baseline EDSS, age, and CSF‐predicted ProgMS probability as predictors. Both baseline EDSS ($\hat{\beta }$ = 0.33, *p* = 0.022) and CSF‐predicted ProgMS probability ($\hat{\beta }$ = 2.9, *p* = 0.002) were statistically significant, whereas age showed only a marginal effect ($\hat{\beta }$ = 0.04, *p* = 0.085). The model explained a substantial proportion of the variance (*R* ^2^ = 0.38).

Thus, the CSF‐based progMS classifier provides independent, non‐redundant predictive value for future disability and, among the variables tested, had the strongest effect size and statistical significance.

In summary, the CSF‐based molecular classifier not only effectively distinguished RRMS from progMS, but also independently predicted greater disability at follow up in clinically diagnosed RRMS patients whom it classified as progMS. Compared with clinical diagnosis, it more frequently assigned younger individuals with high disability and older individuals to the progMS category, reflecting underlying intrathecal biology rather than misclassification.

### Clinical Utility of the CSF‐Based Molecular Prognostic Predictor

Despite prioritization of prospective EDSS documentation, baseline EDSS scores at the time of CSF collection were unavailable for most SPINCOMS participants. Due to the lack of structured clinical notes or case report forms, retrospective EDSS assessments were expected to be imprecise. To address this, in 54 pwMS with available baseline MRIs, we used published COMRIS models^12^, ^13^ to estimate baseline EDSS (see Methods).

**FIGURE 4:**
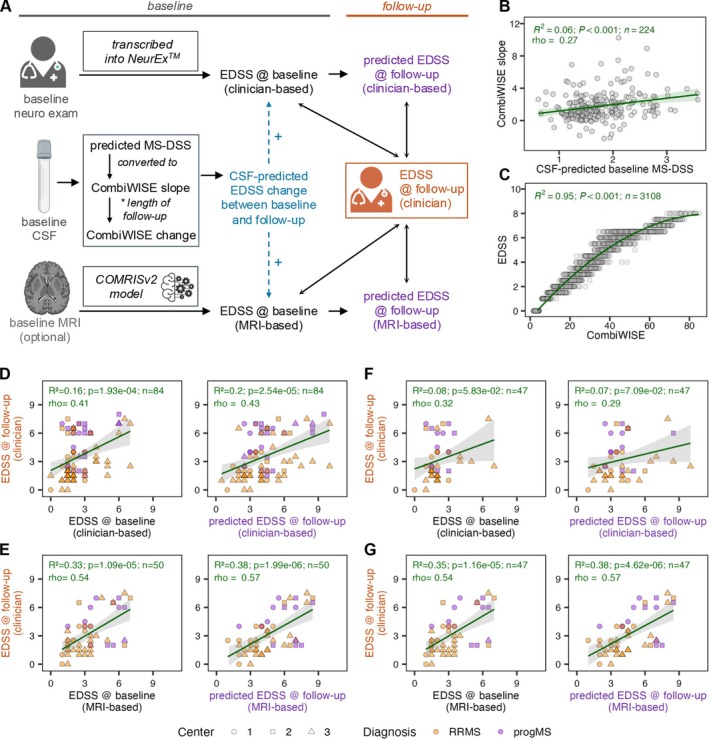
Multiple sclerosis (MS) prognostic model. (A) At baseline, cerebrospinal fluid (CSF) was collected alongside a neurological examination documented in the electronic medical record (EMR), with optional brain and spinal cord magnetic resonance imaging (MRI). The neurological examination was transcribed into the NeurEx™ app to generate an Expanded Disability Status Scale (EDSS) score at baseline. MRIs were reviewed and semi‐quantitatively graded by a neurologist blinded to diagnosis; these MRI scores were inputted into the COMRISv2 machine‐learning model to predict EDSS from imaging. CSF proteomic data (generated via SOMAScan) were inputted into a random forest model predicting the Multiple Sclerosis Disease Severity Scale (MS‐DSS), which was converted into a CombiWISE slope reflecting the rate of disability progression. The predicted CombiWISE slope, combined with the time interval between baseline and follow‐up, was used to estimate change in EDSS. Predicted EDSS at follow‐up was obtained by adding the CSF‐predicted EDSS change to baseline EDSS (either clinician‐ or MRI‐derived). Correlations between baseline EDSS (clinician‐ or MRI‐based) and predicted EDSS at follow up are illustrated by diagonal and vertical double‐headed arrows. (B) In the Neuroimmunological Disease Section's (NDS) longitudinal cohort, CSF‐predicted MS‐DSS correlated with CombiWISE slope; linear regression (green line) was used to estimate CombiWISE slope from baseline MS‐DSS. (C) In the NDS cross‐sectional cohort (*n* = 3,108 visits), CombiWISE strongly correlated with EDSS. A quadratic model (green curve) was fitted to convert CombiWISE change into EDSS change. (D) Left: correlation between clinician‐derived EDSS at baseline and follow‐up (*n* = 84). Right: correlation between clinician‐measured EDSS at follow‐up and CSF‐predicted EDSS at follow‐up using baseline clinician‐derived EDSS (*n* = 84). (E) Left: correlation between MRI‐based EDSS at baseline and clinician‐measured EDSS at follow‐up (n = 50). Right: correlation between clinician‐measured EDSS at follow‐up and CSF‐predicted EDSS at follow‐up using baseline MRI‐based EDSS (n = 50). (F, G) Same comparisons as (D, E), respectively, but restricted to the intersecting cohort with both clinician‐ and MRI‐based baseline EDSS (*n* = 47). In (D–G), shapes represent sample collection centers; orange and purple indicate relapsing–remitting MS (RRMS) and progressive MS (progMS), respectively. [Color figure can be viewed at www.annalsofneurology.org]

The validation concept for the CSF‐based MS severity predictor is illustrated in Figure 4A and described in Methods. Briefly, due to the lack of baseline clinical predictors necessary to directly validate CSF‐based MS severity, we validated the model indirectly by predicting (prospectively measured) EDSS at follow up. To do this, we used a (non‐SPINCOMS) NIH cohort^7^ to generate linear models (Fig 4B), allowing us to convert CSF‐based predictions of annualized progression slopes measured by granular combinatorial weight‐adjusted disability scale (CombiWISE^18^; see methods) into annualized EDSS change. To estimate the predicted change in disability, each slope was multiplied by the follow‐up interval (ie, years between the baseline and follow‐up EDSS measurements). Because this predicted change was often smaller than the minimally detectable 0.5‐point EDSS increment, we used the continuous CombiWISE scale (range 0–100) for intermediate calculations. We then converted CombiWISE change back to EDSS using its strong correlation with EDSS, also derived from the NIH cohort (Fig 4C).

Of the 96 pwMS with CSF data, 7 lacked baseline EDSS and 5 lacked follow‐up EDSS, yielding 84 pwMS with complete datasets for prognostic validation. In this cohort, baseline EDSS correlated weakly with follow‐up EDSS (rho = 0.41, *p* < 0.001), which is consistent with known MS progression rates—approximately 1 EDSS point per decade—and the cohort's mean follow‐up period of 10.2 years. Importantly, adding CSF‐predicted disability change improved the explained variance in follow‐up EDSS from 16% to 21% (Fig 4D), a statistically significant gain (*p* = 0.02).

Among the 54 pwMS with baseline MRI, 4 lacked follow‐up EDSS, resulting in 50 pwMS eligible for COMRISv2 analysis. In this subset, COMRISv2‐predicted baseline EDSS showed a moderate correlation with follow‐up EDSS (rho = 0.54, *p* < 0.001). Adding the CSF‐predicted disability change further increased the explained variance from 33% to 39% (Fig 4E), again reaching statistical significance (*p* = 0.05).

Given the superior predictive performance of MRI‐based EDSS over clinician‐derived EDSS, we tested whether COMRISv2 outperforms clinical examinations for baseline disability measurement. We analyzed 47 pwMS with both retrospective, clinician‐documented neurological examinations (converted to EDSS) and baseline MRIs scored with COMRISv2 (Fig 4F,G). In this group, neither the clinician‐derived baseline EDSS nor the CSF‐adjusted EDSS correlated significantly with follow‐up EDSS (Fig 4F). In contrast, COMRISv2‐predicted EDSS showed a moderate correlation (rho = 0.54, *p* < 0.001), explaining 35% of follow‐up EDSS variance. Adding the CSF‐predicted disability change increased this to 39% (Fig 4G).

These findings support our initial hypothesis: when neurological examinations are recorded as free‐text in medical records, they cannot be reliably translated into structured disability scales such as EDSS. In such cases, clinical‐grade MRIs, if available, can be semiquantitatively scored using validated protocols,^12^, ^13^ and converted into physical and cognitive disability estimates via COMRIS models. All COMRIS models were internally validated, and here we provide external validation of the EDSS COMRISv2 model in the SPINCOMS cohort.

In conclusion, whether using the full cohort of 84 pwMS with clinical EDSS data or the subset of 50 with COMRISv2‐based EDSS estimates, we confirmed that incorporating CSF‐predicted disability change improves prediction of follow‐up EDSS.

## Discussion

Diagnosing MS remains challenging due to the overlap of its clinical and radiological features with those of other neurological disorders.^22^ MS pathogenesis involves early and persistent inflammation, neuroaxonal injury, and a heterogeneous interplay with aging, neuroplasticity, and remyelination, all contributing to its variable clinical course.^23^ In this context, CSF proteomics offers a valuable window into these biological processes and can guide individualized therapeutic decisions.

In this multicenter study, we leveraged decades of cryopreserved CSF samples from 3 North American MS centers to externally validate previously developed CSF‐based molecular classifiers. These tests differentiate MS from other central nervous system pathologies, and distinguish RRMS from ProgMS at disease onset. In addition, we integrated CSF proteomic classifiers with an MRI‐based algorithm (COMRISv2) to predict EDSS measured by clinicians at follow‐up visit.

Despite slightly lower performance than in prior single‐center internal validation (AUROC 0.94 vs 0.98 for MS diagnosis; 0.76 vs 0.91 for ProgMS), both CSF‐based diagnostic tests demonstrated strong clinical utility, especially when compared with older MRI‐based MS diagnostic tools.^3^ To better understand the sources of misclassification, we examined available data and, when possible, consulted the patients’ neurologists for updated clinical perspectives.

Consistent with the original study,^6^ most pwMS who were classified as non‐MS lacked evidence of intrathecal adaptive immune activation, specifically, elevated IgG index, OCB, and sCD27 levels. CSF biomarkers measured by the 1.3 k SOMAscan that reflect adaptive immunity (eg, CD48, CXCL10, CXCL13, ICOS, IgA, IgM, IL2RG, KLKR1, LY9, SLAMF7, TNFRSF17) were either within the normal range or only marginally elevated in 2 of the 3 misclassified patients, suggesting likely non‐inflammatory etiologies.

One of these patients is still alive, and the treating neurologist has acknowledged diagnostic uncertainty. The second patient is deceased. Although the treating neurologist believed the diagnosis of MS was correct, we identified the following red flags: (1) the absence of intrathecal B‐cell/plasma cell activation based on SOMAscan and sCD27 data (IgG index and OCB were not assessed at diagnosis); (2) atypical MRI findings for MS: a 59‐year‐old woman with confluent supratentorial white matter involvement consistent with ischemic‐gliotic disease, a basal ganglia lacunar infarct, but lacking spinal cord involvement or contrast‐enhancing lesions; (3) discrepancy between markedly elevated CSF NFL (10,830 pg/mL) and absence of contrast‐enhancing lesions—such high NFL levels are typically seen in active MS with contrast‐enhancing lesions or in neurodegenerative disorders; and (4) atypical clinical course with rapid progression to dementia, hallucinations, and death within 12 years of diagnosis. Together, these features suggest a non‐inflammatory neurodegenerative disorder as a more likely diagnosis.

The third misclassified patient, who also lacked OCB and had a normal IgG index, showed marked CSF pleocytosis (120 white blood cells) and elevated albumin quotient—both atypical for MS. The clinical presentation included unilateral optic neuritis and longitudinally extensive transverse myelitis from T1‐11 and C5‐6 with patchy enhancement. Neuromyelitis optica‐IgG was negative in both the CSF and serum, but myelin oligodendrocyte glycoprotein antibody testing was not available at the time of diagnosis. Brain MRI revealed only 3 tiny, non‐evolving lesions over 2 years, whereas the spinal cord lesions resolved. Unfortunately, the treating neurologist is deceased and could not be consulted. The most likely diagnosis is a neuromyelitis optica spectrum disorder, possibly myelin oligodendrocyte glycoprotein antibody‐associated disease, where resolution of spinal cord lesions can occur.

Interestingly, the molecular classifier correctly identified this case as non‐MS, despite elevated levels of several CSF biomarkers associated with adaptive immunity (CD48, CXCL10, CXCL13, IgA, LY9, SLAMF7, TNFRSF17, and sCD27), proving that it captures disease‐specific immune patterns rather than inflammation alone.

The CSF‐based progMS classifier identified more individuals as having progMS than were clinically diagnosed as such at the time of CSF collection, a discrepancy particularly evident in older pwMS. This likely reflects the well‐established role of aging in driving MS progression, through mechanisms such as reduced immune‐mediated repair and diminished central nervous system compensatory capacity leading to loss of brain reserve.^24^ Importantly, the model's continuous output—the CSF‐predicted probability of progMS—was the strongest predictor of follow‐up EDSS in the multivariate model, highlighting the limitations of dichotomizing what is fundamentally a biologically continuous process. We interpret the statistically robust, non‐redundant association of CSF‐based progMS probability with follow‐up EDSS as evidence that the classifier captures intrathecal biology linked to disease progression, and can detect the transition to progMS with greater sensitivity than is possible through clinical observation alone. This feature may therefore be particularly valuable for patient management and for guiding the development of targeted therapies for progMS.

Together, these 2 CSF‐based diagnostic models—1 for distinguishing MS from phenotypic mimics and the other for staging disease at the time of its clinical presentation—offer a powerful, biologically grounded approach for improving diagnostic precision and early disease stratification in MS.

We also validated the CSF‐based prognostic model, by predicting future disability change based on the baseline CSF proteome. Using the COMRISv2 algorithm to estimate baseline EDSS and integrating CSF‐predicted disability slopes, we achieved a moderate correlation with follow‐up EDSS, explaining 40% of the variance, consistent with prior internal validation.^7^ When we similarly evaluated the predictive power of CSF NFL, we found an inverse association (data not shown): pwMS with higher NFL levels at diagnosis tended to progress less. This likely reflects the fact that NFL is predominantly released from newly forming lesions, which are more common in RRMS and associated with slower long‐term progression compared with prog MS. Finally, we externally validated that semiquantitative grading of clinical MRIs as input to COMRISv2 model predicts clinician‐derived EDSS. Thus, this study externally validated 4 machine‐learning models of clinical utility for MS: 3 CSF‐proteomic and 1 MRI‐based.

A major strength of this external validation study was its multicenter design, which relied on clinical and imaging data commonly available in routine practice. Additional strengths include prospective follow‐up with structured EDSS assessments, blinded data generation (ie, clinicians assessing follow‐up EDSS were blinded to CSF and MRI outcomes, and vice versa), as well as predefined power calculations and outcome measures.

However, the study also had notable limitations. First, the COVID‐19 pandemic disrupted in‐person follow‐up visits, limiting our ability to assess disability and to collect functional outcomes (T25FW, 9HPT, SDMT) in all participants. Second, the study population represents a convenience sample—pwMS who consented to CSF biobanking at the time of diagnosis and were willing to return for follow‐up. This may have reduced the quality of baseline clinical data and contributed to underreporting of progMS diagnoses, which may have been intentionally avoided to facilitate access to DMTs at a time when no treatments were approved for progMS. We also note that multiplex proteomic platforms, including SOMAscan, continue to evolve: expanding the number of measurable proteins, improving assay accuracy, and increasing the proportion of reagents with orthogonal validation. For example, the latest SOMAscan version quantifies >11,000 protein variants without a measurable loss in accuracy, and approximately 80% of reagents now have orthogonal validation.^25^, ^26^, ^27^ However, substantial methodological differences between the 1.1 and 1.3 k assay versions, including the removal of a key reagent, required recalibration of the MS diagnostic model and likely contributed to a mild reduction in its diagnostic performance. Importantly, these limitations collectively more likely attenuated effect sizes and inflated *p* values, rather than inflating associations.

At a time when most omics studies in MS lack even internal validation cohorts,^28^, ^29^ this multicenter external validation study delivers a compelling proof‐of‐concept: CSF biomarkers harbor rich molecular information about intrathecal disease mechanisms that can be harnessed to accurately diagnose, stage, and differentiate MS from its clinical mimics—while also offering prognostic insight. Beyond validating specific CSF‐based models, this study lays the groundwork for future clinical and commercial applications of CSF proteomics to personalize and optimize care for people with neurological diseases.

## Author Contributions

E.A., A.H.C., M.S.F., L.P., and B.B. contributed to the conception and design of the study; L.G., P.K., M.G., C.F., F.P., L.P., B.B. contributed to the acquisition and analysis of data; L.G., P.K., M.G., E.A., A.H.C., M.S.F., L.P., B.B. contributed to drafting the text and/or preparing the figures. [Correction added on 09 February 2026, after first online publication: Author contribution text has been revised in this version.]

## Potential Conflicts of Interest

Nothing to report.

## Supporting information


**Data S1** Supporting Information.

**Data S2** Final Results of SPINCOMS Validation Data.

## Data Availability

All analysis codes along with raw proteomic data and predictive model results are available as Supporting Information.
