# Supplementary material for: From Diagnosis to Disease Staging: Multisite Validation of Cerebrospinal Fluid Molecular Tests in Multiple Sclerosis
Source: Ann Neurol. 2025 Oct 3;99(2):328–40. doi: 10.1002/ana.78047 (PMC12894485; doi:10.1002/ana.78047)
Supplement: Supplementary file 2 — Data S2 Final Results of SPINCOMS Validation Data. [file ANA-99-328-s001.docx]

Final Results of SPINCOMS Validation Data

[All authors]

Updated JUly 23, 2025

## Data loading and wrangling

library(readxl)
SPINCOMS1 <- read_excel("SPINCOMS_RAW_DATA_wOCB_FINAL.xlsx",
 sheet = "CSF diagnostic test results", na = "NA") %>%
 clean_names()

SPINCOMS2 <- read_excel("SPINCOMS_RAW_DATA_wOCB_FINAL.xlsx",
 sheet = "Prognostic data - clinician") %>%
 clean_names()

## AUROC results for validation data: MS/Non-MS: Figure 2

- Version from the _Final.xslx read in as SPINCOMS1

df <- SPINCOMS1 %>%
 mutate(clinical_diagnosis = factor(clinical_diagnosis),
 csf_dichotomized_ms_classification = factor(csf_dichotomized_ms_classification))
# this can be found in the raw data spreadsheet under "CSF diagnostic test results"

# 2) Build a four‑level diagnosis_simple:
# • SP‑MS, PP‑MS → "progMS"
# • RR‑MS → "RRMS"
# • NIND,OIND → unchanged
df <- df %>%
 mutate(diagnosis_simple = clinical_diagnosis,
 diagnosis_simple = forcats::fct_collapse(diagnosis_simple,
 progMS = c("PP-MS", "SP-MS")),
 diagnosis_simple = forcats::fct_recode(diagnosis_simple,
 RRMS = "RR-MS"),
 diagnosis_simple = relevel(diagnosis_simple, "OIND"))

# 3) Misclassification at 50% cutoff

tally(diagnosis_simple ~ csf_dichotomized_ms_classification, data = df)

## csf_dichotomized_ms_classification
## diagnosis_simple MS non-MS
## OIND 14 16
## NIND 13 21
## progMS 31 0
## RRMS 62 3

df <- df %>%
 mutate(correct = case_when(
 # True MS called MS
 diagnosis_simple %in% c("progMS","RRMS") & csf_dichotomized_ms_classification == "MS" ~ TRUE,
 # True non‑MS called non‑MS
 diagnosis_simple %in% c("NIND","OIND") & csf_dichotomized_ms_classification == "non-MS" ~ TRUE,
 TRUE ~ FALSE
 )
 )

tally(correct ~ diagnosis_simple|csf_dichotomized_ms_classification, data = df)

## , , csf_dichotomized_ms_classification = MS
##
## diagnosis_simple
## correct OIND NIND progMS RRMS
## TRUE 0 0 31 62
## FALSE 14 13 0 0
##
## , , csf_dichotomized_ms_classification = non-MS
##
## diagnosis_simple
## correct OIND NIND progMS RRMS
## TRUE 16 21 0 0
## FALSE 0 0 0 3

misclass_ms <- df %>%
 group_by(diagnosis_simple) %>%
 summarize(
 total = n(),
 wrong = sum(!correct),
 pct_wrong = round(wrong / total * 100, 1)
 )
print(misclass_ms)

## # A tibble: 4 × 4
## diagnosis_simple total wrong pct_wrong
## <fct> <int> <int> <dbl>
## 1 OIND 30 14 46.7
## 2 NIND 34 13 38.2
## 3 progMS 31 0 0
## 4 RRMS 65 3 4.6

# A tibble: 4 × 4
# diagnosis_simple total wrong pct_wrong
# <fct> <int> <int> <dbl>
# 1 OIND … … …
# 2 NIND … … …
# 3 progMS … … …
# 4 RRMS … … …

# 4) Overall AUROC
df <- df %>%
 mutate(true_label = ifelse(diagnosis_simple %in% c("progMS","RRMS"), 1, 0))

roc_ms <- roc(df$true_label, df$csf_predicted_ms_probability)
auc_ms <- auc(roc_ms)
ci_ms <- ci.auc(roc_ms)
cat(sprintf(
 "MS vs non‑MS AUROC: %.3f (95%% CI %.3f–%.3f)\n",
 auc_ms, ci_ms[1], ci_ms[3]
))

## MS vs non‑MS AUROC: 0.937 (95% CI 0.899–0.974)

roc.area(df$true_label, df$csf_predicted_ms_probability)

## $A
## [1] 0.9366048
##
## $n.total
## [1] 160
##
## $n.events
## [1] 96
##
## $n.noevents
## [1] 64
##
## $p.value
## [1] 4.734573e-21

# 5) Center‑specific AUROCs
aucs_ms_by_center <- df %>%
 group_by(center) %>%
 summarize(
 auc = as.numeric(auc( roc(true_label, csf_predicted_ms_probability) )),
 ci_low = ci.auc(roc(true_label, csf_predicted_ms_probability))[1],
 ci_high = ci.auc(roc(true_label, csf_predicted_ms_probability))[3]
 )
print(aucs_ms_by_center)

## # A tibble: 3 × 4
## center auc ci_low ci_high
## <dbl> <dbl> <dbl> <dbl>
## 1 1 0.963 0.889 1
## 2 2 0.968 0.917 1
## 3 3 0.900 0.835 0.964

- Figure 2A: Overall MS/not AUc

get_roc_plot <- function(df) {
 roc_obj <- roc(df$true_label, df$csf_predicted_ms_probability)
 ci_obj <- ci.auc(roc_obj)
 roc_df <- tibble(
 fpr = rev(roc_obj$specificities),
 tpr = rev(roc_obj$sensitivities)
 )

 ggplot(roc_df, aes(fpr, tpr)) +
 geom_line(size = 0.75) +
 geom_abline(slope = 1, intercept = 1, linetype = "dashed", color = "darkgray") +
 scale_x_reverse() +
 labs(x = "False Positive Rate", y = "True Positive Rate") +
 annotate(
 "text", x = 0.66, y = 0.68,
 label = sprintf(
 "AUROC = %.2f\n95%% CI: %.2f–%.2f",
 auc(roc_obj), ci_obj[1], ci_obj[3]
 ),
 color = "red", size = 3.5
 ) +
 base_theme + theme(legend.position = "none")
}

p2A <- get_roc_plot(df)
p2A


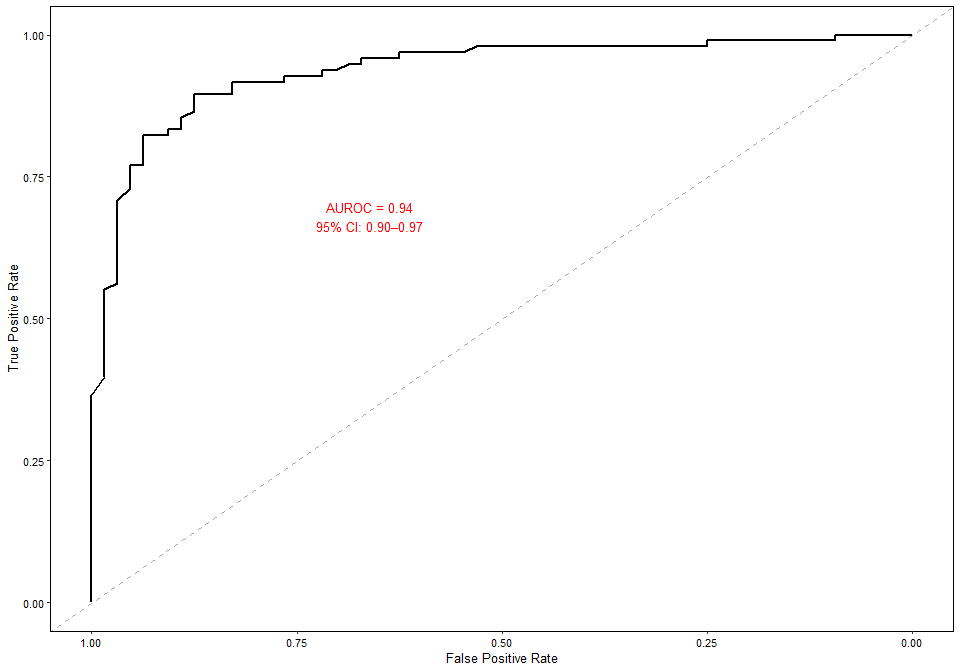


ggsave(p2A, filename = "./MS_classifier_AUROC.png", width = 3.5, height = 2.5, units = "in")

- Figure 2B: MS/not AUC by center:

colors <- c("magenta", "orange", "blue")
ci_list <- vector("list", 3)

roc_center_plot <- ggplot() +
 geom_abline(slope = 1, intercept = 1, linetype = "dashed", color = "darkgray")

for (i in 1:3) {
 df_c <- filter(df, center == i)
 roc_o <- roc(df_c$true_label, df_c$csf_predicted_ms_probability)
 ci_i <- ci.auc(roc_o)
 roc_df <- tibble(
 fpr = rev(roc_o$specificities),
 tpr = rev(roc_o$sensitivities)
 )
 ci_list[[i]] <- ci_i
 roc_center_plot <- roc_center_plot +
 geom_line(data = roc_df, aes(fpr, tpr), color = colors[i], size = 0.75)
}

roc_center_plot <- roc_center_plot +
 scale_x_reverse() +
 labs(x = "False Positive Rate", y = "True Positive Rate") +
 annotate(
 "rect", xmin = 0, xmax = 0.53, ymin = 0, ymax = 0.6,
 fill = "white", alpha = 0.75
 ) +
 annotate(
 "text", x = 0.23, y = c(0.5, 0.3, 0.1),
 label = sprintf(
 "Center %d: AUROC = %.2f\n95%% CI: %.2f–%.2f",
 1:3,
 map_dbl(1:3, ~ auc(roc(filter(df, center == .x)$true_label,
 filter(df, center == .x)$csf_predicted_ms_probability))),
 map_dbl(ci_list, ~ .x[1]),
 map_dbl(ci_list, ~ .x[3])
 ),
 color = colors, size = 3
 ) +
 base_theme + theme(legend.position = "none")


roc_center_plot


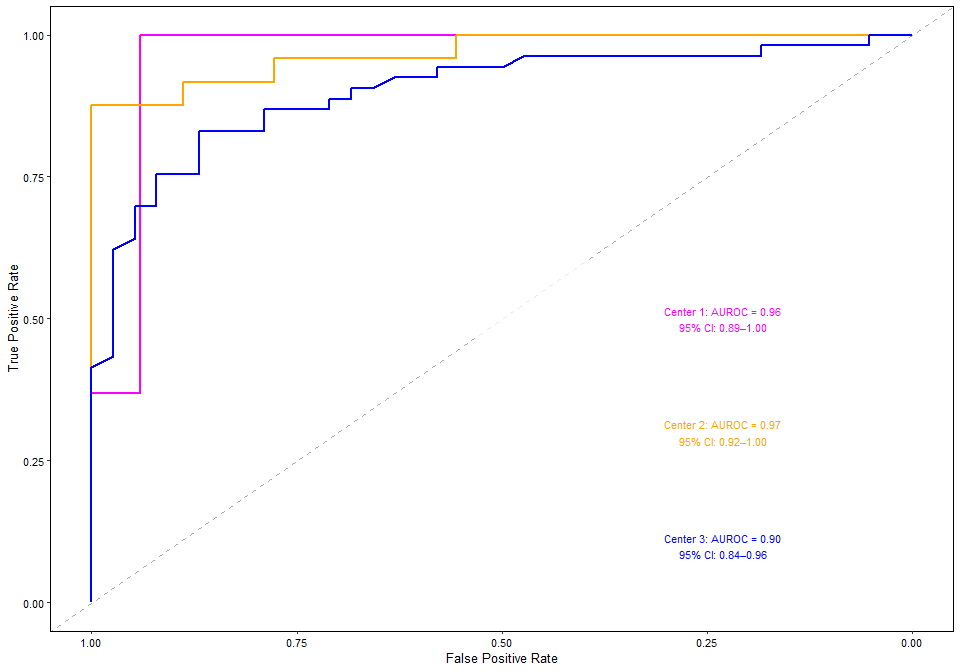


ggsave(roc_center_plot, filename = "./MS_classifier_AUROC_centers.png", width = 3.5, height = 2.5, units = "in")

- Comparison to traditional CSF biomarkers:

# Keep only rows with both OCB and IgG index info
df_othercsf <- df %>% drop_na(ocb, ig_g_index) %>% #n = 121
 mutate(igg_index_abnormal = factor(is_the_ig_g_index_out_of_range))

# Continuous predictor
y_true <- df_othercsf$true_label
p_cont <- df_othercsf$csf_predicted_ms_probability

roc_cont <- roc(y_true, p_cont)
ci_cont <- ci.auc(roc_cont)

# ---- Binary tests ----
ocb_bin <- if_else(df_othercsf$ocb == "Yes", 1L, 0L)
igg_bin <- if_else(df_othercsf$igg_index_abnormal == "Yes", 1L, 0L)
both_bin <- if_else(ocb_bin + igg_bin > 0, 1L, 0L)

tally(both_bin ~ igg_bin|ocb_bin)

## , , ocb_bin = 0
##
## igg_bin
## both_bin 0 1
## 0 40 0
## 1 0 5
##
## , , ocb_bin = 1
##
## igg_bin
## both_bin 0 1
## 0 0 0
## 1 26 50

# Helper to summarize AUCs and DeLong p-value
compare_to_cont <- function(bin_vec, name){
 roc_bin <- roc(y_true, bin_vec)
 test <- roc.test(roc_cont, roc_bin, method = "delong")
 tibble::tibble(
 Test = name,
 AUC_cont = as.numeric(auc(roc_cont)),
 AUC_bin = as.numeric(auc(roc_bin)),
 p_diff = as.numeric(test$p.value)
 )
}

summary_tbl <- dplyr::bind_rows(
 compare_to_cont(ocb_bin, "OCB"),
 compare_to_cont(igg_bin, "IgG index"),
 compare_to_cont(both_bin, "OCB + IgG")
)

print(summary_tbl)

## # A tibble: 3 × 4
## Test AUC_cont AUC_bin p_diff
## <chr> <dbl> <dbl> <dbl>
## 1 OCB 0.929 0.821 0.00328
## 2 IgG index 0.929 0.703 0.0000000220
## 3 OCB + IgG 0.929 0.785 0.000922

- Figure 2C: Clinical diagnosis by predicted MS probability

# 3. Added data wrangling------------------------------------------------

df <- df %>%
 mutate(
 center = factor(center, levels = c(1, 2, 3)),
 ms_classification_correct = case_when(
 diagnosis_simple %in% c("progMS", "RRMS") &
 csf_dichotomized_ms_classification == "MS" ~ "correct",
 diagnosis_simple %in% c("progMS", "RRMS") &
 csf_dichotomized_ms_classification == "non-MS" ~ "incorrect",
 diagnosis_simple %in% c("NIND", "OIND") &
 csf_dichotomized_ms_classification == "MS" ~ "incorrect",
 diagnosis_simple %in% c("NIND", "OIND") &
 csf_dichotomized_ms_classification == "non-MS" ~ "correct",
 TRUE ~ NA_character_
 ),
 true_label = if_else(
 clinical_diagnosis %in% c("OIND", "NIND"),
 0, 1
 )
 )

# Compute percentages for annotation
percs <- df %>%
 group_by(diagnosis_simple) %>%
 summarize(
 perc_no = scales::percent(sum(csf_predicted_ms_probability < 0.5) / n()),
 perc_yes = scales::percent(sum(csf_predicted_ms_probability > 0.5) / n()),
 .groups = "drop"
 )

# 4. Panel C: MS probability by diagnosis -------------------------------------
p2C <- df %>%
 ggplot(aes(diagnosis_simple, csf_predicted_ms_probability)) +
 geom_violin(fill = NA, color = "darkgray", size = 0.25) +
 geom_jitter(
 aes(shape = center, fill = ms_classification_correct, color = ms_classification_correct),
 position = position_jitter(width = 0.25), size = 2, alpha = 0.75, stroke = 0.2
 ) +
 scale_y_continuous(
 limits = c(0, 1.05),
 breaks = seq(0, 1, 0.25),
 labels = scales::percent
 ) +
 scale_shape_manual(values = c(21, 22, 24), labels = c("1", "2", "3"), name = "Center") +
 scale_fill_manual(
 values = c("#4CBB17", "salmon"),
 breaks = c("correct", "incorrect"),
 name = "CSF classification"
 ) +
 scale_color_manual(
 values = c("black", "red"),
 breaks = c("correct", "incorrect"),
 name = "CSF classification"
 ) +
 geom_hline(yintercept = 0.5, color = "blue", size = 0.25) +
 annotate(
 "text",
 x = rep(percs$diagnosis_simple, each = 2),
 y = rep(c(0, 1.05), times = 4),
 label = as.vector(t(as.matrix(percs[,2:3]))),
 color = c(rep(c("darkgreen", "salmon"), times = 2), rep(c("salmon", "darkgreen"), times = 2)),
 size = 3.5
 ) +
 coord_flip() +
 labs(x = "Clinical diagnosis", y = "MS probability (%)") +
 guides(fill = guide_legend(override.aes = list(shape = 21))) +
 base_theme

p2C


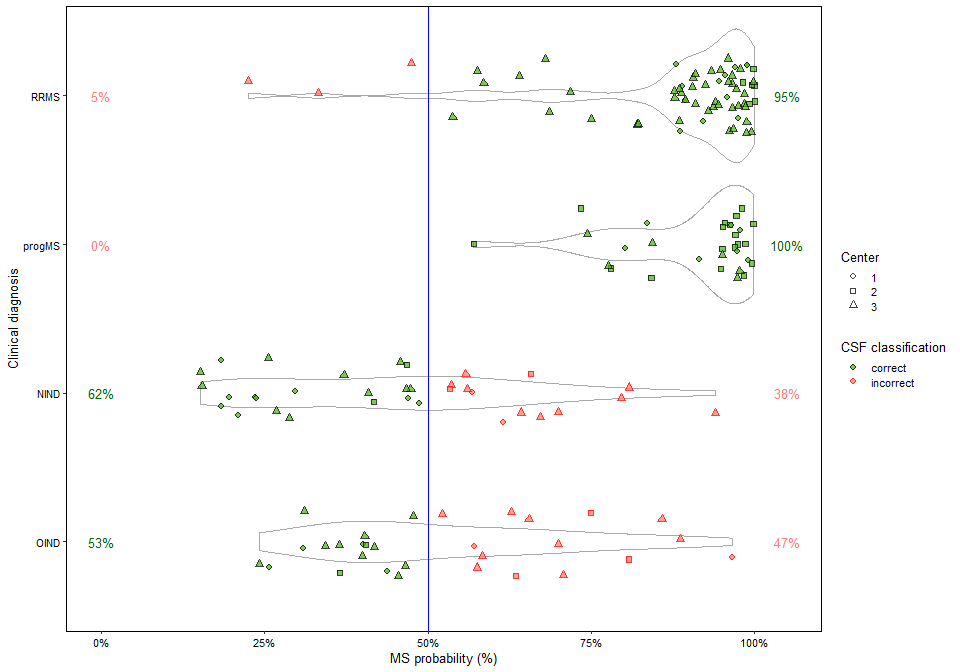


ggsave(p2C, filename = "./MS_classifier_probability.png", width = 7, height = 2.5, units = "in")

- Figure 2D: Clinical diagnosis by sCD27 (left) and IgG index (right)

#dfsCD <- df %>% drop_na(s_cd27_ng_ml)

#dim(dfsCD) #n = 160


hd_med <- 8.3
hd_sd <- 3.53

p2D_Left <- df %>%
 ggplot(aes(diagnosis_simple, log10(s_cd27_ng_ml))) +
 annotate(
 "rect",
 ymin = -Inf, ymax = log10(hd_med + 2 * hd_sd),
 xmin = -Inf, xmax = Inf,
 fill = "blue", alpha = 0.15
 ) +
 geom_violin(fill = NA, color = "gray", size = 0.25) +
 geom_jitter(
 aes(shape = center, fill = ms_classification_correct),
 position = position_jitter(width = 0.2), size = 2, alpha = 0.75, stroke = 0.2
 ) +
 geom_hline(
 yintercept = log10(c(hd_med - 2*hd_sd, hd_med, hd_med + 2*hd_sd)),
 linetype = c("dashed", "solid", "dashed"), color = "blue", size = 0.25
 ) +
 ylim(0.7, 3.3) +
 scale_shape_manual(values = c(21, 22, 24), labels = c("1", "2", "3"), name = "Center") +
 scale_fill_manual(
 values = c("#4CBB17", "salmon"),
 breaks = c("correct", "incorrect"),
 name = "CSF classification"
 ) +
 coord_flip() +
 labs(x = "Clinical diagnosis", y = "sCD27 [ng/mL] (log10)") +
 base_theme + theme(legend.position = "none")

ggsave(p2D_Left, filename = "./sCD27_log10.png", width = 3.5, height = 2.25, units = "in")

#IgG Index -------------------------------------------------------
p2D_Right <- df %>%
 ggplot(aes(diagnosis_simple, ig_g_index)) +
 annotate(
 "rect",
 ymin = -Inf, ymax = 0.62,
 xmin = -Inf, xmax = Inf,
 fill = "blue", alpha = 0.15
 ) +
 geom_violin(fill = NA, color = "gray", size = 0.25) +
 geom_jitter(
 aes(shape = center, fill = ms_classification_correct),
 position = position_jitter(width = 0.2), size = 2, alpha = 0.75, stroke = 0.2
 ) +
 geom_hline(yintercept = 0.62, color = "blue", size = 0.25) +
 ylim(0.11, 4.05) +
 scale_shape_manual(values = c(21, 22, 24), labels = c("1", "2", "3"), name = "Center") +
 scale_fill_manual(
 values = c("#4CBB17", "salmon"),
 breaks = c("correct", "incorrect"),
 name = "CSF classification"
 ) +
 coord_flip() +
 labs(x = "Clinical diagnosis", y = "IgG Index") +
 guides(fill = guide_legend(override.aes = list(shape = 21)))+
 base_theme +
 theme(
 legend.position = "right"
 )

p2D_Left + p2D_Right


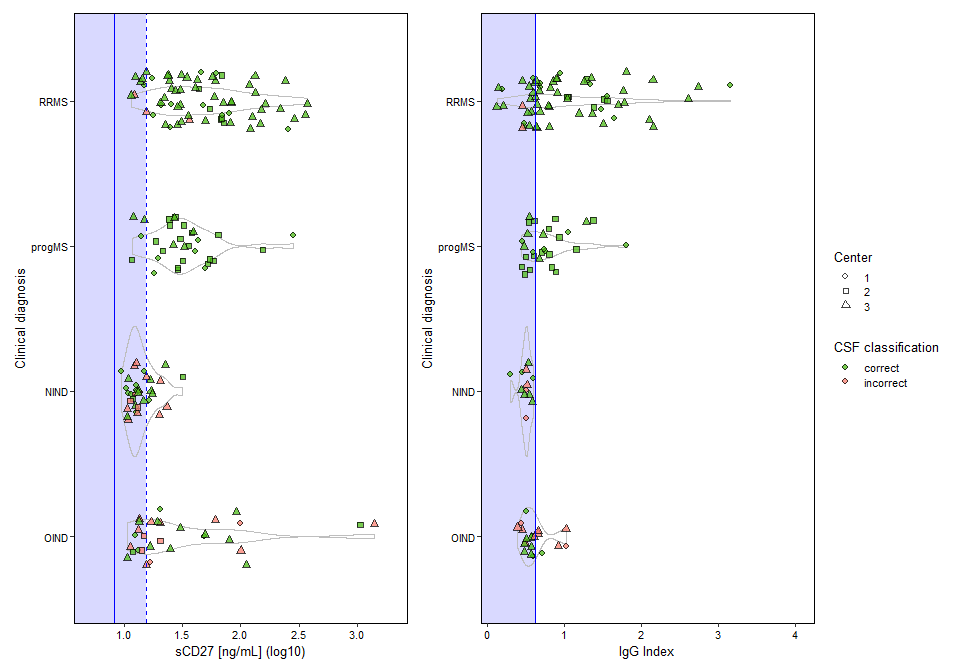


ggsave(p2D_Right, filename = "./IgG_index.png", width = 4.2, height = 2.25, units = "in")

# AUC results for Progressive MS vs RRMS: Figure 3

# 1) Load predictions & keep only MS patients
dfMSonly <- df %>%
 dplyr::filter(clinical_diagnosis %in% c("PP-MS","RR-MS","SP-MS"))

dim(dfMSonly)

## [1] 96 20

# 2) Same four‑level diagnosis_simple (only progMS & RRMS survive)
dfMSonly <- dfMSonly %>%
 mutate(
 diagnosis_simple = factor(diagnosis_simple, levels = c("progMS","RRMS"))
 )

# 3) Misclassification at 50% cutoff on progressive_ms_classification
dfMSonly <- dfMSonly %>%
 mutate(
 correct = case_when(
 diagnosis_simple == "progMS" & csf_dichotomized_progressive_ms_classification == "Progressive MS" ~ TRUE,
 diagnosis_simple == "RRMS" & csf_dichotomized_progressive_ms_classification == "RRMS" ~ TRUE,
 TRUE ~ FALSE
 )
 )

misclass_prog <- dfMSonly %>%
 group_by(diagnosis_simple) %>%
 summarize(
 total = n(),
 wrong = sum(!correct),
 pct_wrong = round(wrong / total * 100, 1)
 )
print(misclass_prog)

## # A tibble: 2 × 4
## diagnosis_simple total wrong pct_wrong
## <fct> <int> <int> <dbl>
## 1 progMS 31 3 9.7
## 2 RRMS 65 32 49.2

# A tibble: 2 × 4
# diagnosis_simple total wrong pct_wrong
# <fct> <int> <int> <dbl>
# 1 progMS … … …
# 2 RRMS … … …

# 4) Overall AUROC
dfMSonly <- dfMSonly %>%
 mutate(true_label = ifelse(diagnosis_simple == "progMS", 1, 0))

roc_prog <- roc(dfMSonly$true_label, dfMSonly$csf_predicted_progressive_ms_probability)
auc_prog <- auc(roc_prog)
ci_prog <- ci.auc(roc_prog)
cat(sprintf(
 "Progressive vs RRMS AUROC: %.3f (95%% CI %.3f–%.3f)\n",
 auc_prog, ci_prog[1], ci_prog[3]
))

## Progressive vs RRMS AUROC: 0.762 (95% CI 0.664–0.859)

roc.area(dfMSonly$true_label, dfMSonly$csf_predicted_progressive_ms_probability)

## $A
## [1] 0.7617866
##
## $n.total
## [1] 96
##
## $n.events
## [1] 31
##
## $n.noevents
## [1] 65
##
## $p.value
## [1] 1.818244e-05

# 5) Center‑specific AUROCs
aucs_prog_by_center <- dfMSonly %>%
 group_by(center) %>%
 summarize(
 auc = as.numeric(auc( roc(true_label, csf_predicted_progressive_ms_probability) )),
 ci_low = ci.auc(roc(true_label, csf_predicted_progressive_ms_probability))[1],
 ci_high = ci.auc(roc(true_label, csf_predicted_progressive_ms_probability))[3]
 )
print(aucs_prog_by_center)

## # A tibble: 3 × 4
## center auc ci_low ci_high
## <fct> <dbl> <dbl> <dbl>
## 1 1 0.762 0.524 1
## 2 2 0.759 0.516 1
## 3 3 0.631 0.429 0.833

- Figure 3A: Progressive/RRMS AUC overall

dfMSonly <- dfMSonly %>%
 mutate(
 prog_ms_classification_correct = case_when(
 diagnosis_simple == "progMS" &
 csf_dichotomized_progressive_ms_classification == "Progressive MS" ~ "correct",
 diagnosis_simple == "progMS" &
 csf_dichotomized_progressive_ms_classification == "RRMS" ~ "incorrect",
 diagnosis_simple == "RRMS" &
 csf_dichotomized_progressive_ms_classification == "RRMS" ~ "correct",
 diagnosis_simple == "RRMS" &
 csf_dichotomized_progressive_ms_classification == "Progressive MS" ~ "incorrect",
 TRUE ~ NA_character_
 ),
 true_label = if_else(diagnosis_simple == "RRMS", 0, 1)
 )

# Compute percentages for annotation (threshold = 0.5)
percs <- dfMSonly %>%
 group_by(diagnosis_simple) %>%
 summarize(
 perc_no = scales::percent(sum(csf_predicted_progressive_ms_probability < 0.5) / n()),
 perc_yes = scales::percent(sum(csf_predicted_progressive_ms_probability > 0.5) / n()),
 .groups = "drop"
 )


# Panel A: AUROC plot -----------------------------------------------------
get_roc_plot <- function(df, prob_col) {
 roc_obj <- roc(df$true_label, df[[prob_col]])
 ci_obj <- ci.auc(roc_obj)
 roc_df <- tibble(
 fpr = rev(roc_obj$specificities),
 tpr = rev(roc_obj$sensitivities)
 )

 ggplot(roc_df, aes(x = fpr, y = tpr)) +
 geom_line(size = 0.75) +
 geom_abline(slope = 1, intercept = 1, linetype = "dashed", color = "darkgray") +
 scale_x_reverse() +
 labs(x = "False Positive Rate", y = "True Positive Rate") +
 annotate(
 "text", x = 0.3, y = 0.25,
 label = sprintf("AUROC = %.2f\n95%% CI: %.2f–%.2f",
 auc(roc_obj), ci_obj[1], ci_obj[3]),
 color = "red", size = 3.5
 ) +
 base_theme + theme(legend.position = "none")
}

p3A <- get_roc_plot(dfMSonly, "csf_predicted_progressive_ms_probability")
p3A


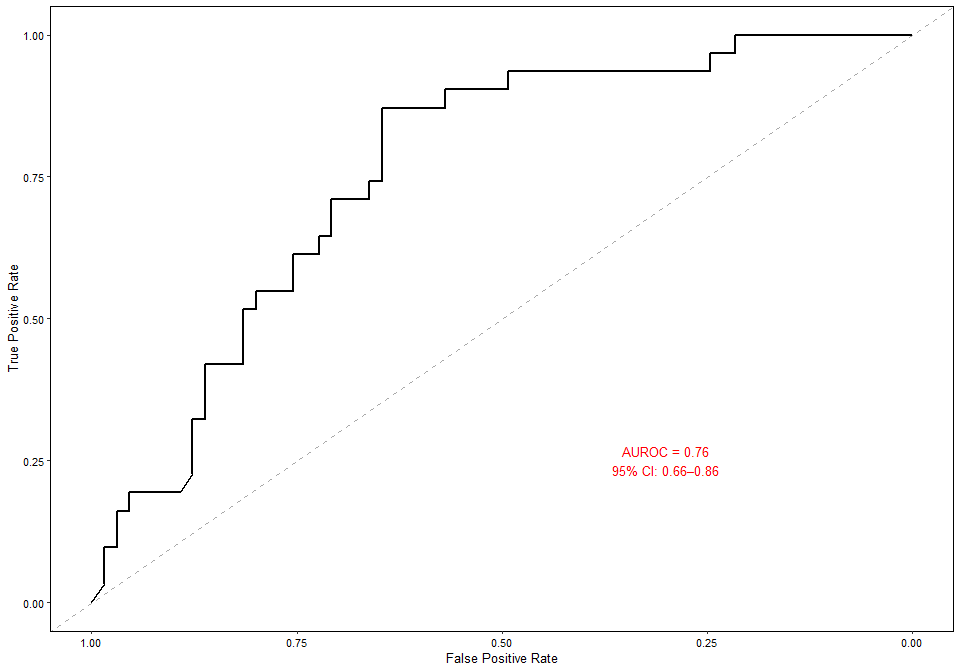


ggsave(p3A, filename = "./prog_MS_AUROC.png", width = 2.75, height = 2.5, units = "in")

- Figure 3B: Clinical diagnosis by predicted progressive probability

#Probability by diagnosis ---------------------------------------

p3B <- dfMSonly %>%
 ggplot(aes(x = diagnosis_simple, y = csf_predicted_progressive_ms_probability)) +
 geom_violin(fill = NA, color = "darkgray", size = 0.25) +
 geom_jitter(
 aes(shape = center, fill = prog_ms_classification_correct, color = ms_classification_correct),
 position = position_jitter(width = 0.25), size = 2, alpha = 0.75, stroke = 0.2
 ) +
 scale_y_continuous(limits = c(-0.1, 1.15), breaks = seq(0, 1, 0.25), labels = scales::percent) +
 scale_shape_manual(values = c(21, 22, 24), labels = c("1", "2", "3"), name = "Center") +
 scale_fill_manual(values = c("#4CBB17", "salmon"), breaks = c("correct", "incorrect"), name = "CSF classification") +
 scale_color_manual(values = c("black", "red"), breaks = c("correct", "incorrect"), name = "CSF classification") +
 geom_hline(yintercept = 0.5, color = "blue", size = 0.25) +
 annotate(
 "text",
 x = rep(percs$diagnosis_simple, each = 2),
 y = rep(c(-0.05, 1.1), times = nrow(percs)),
 label = as.vector(t(as.matrix(percs[,2:3]))), #c(percs$perc_no, percs$perc_yes),
 color = c("salmon", "darkgreen","darkgreen","salmon"),
 size = 3.5
 ) +
 coord_flip() +
 labs(x = "Clinical diagnosis", y = "Progressive MS probability (%)") +
 guides(fill = guide_legend(override.aes = list(shape = 21))) +
 base_theme

p3B


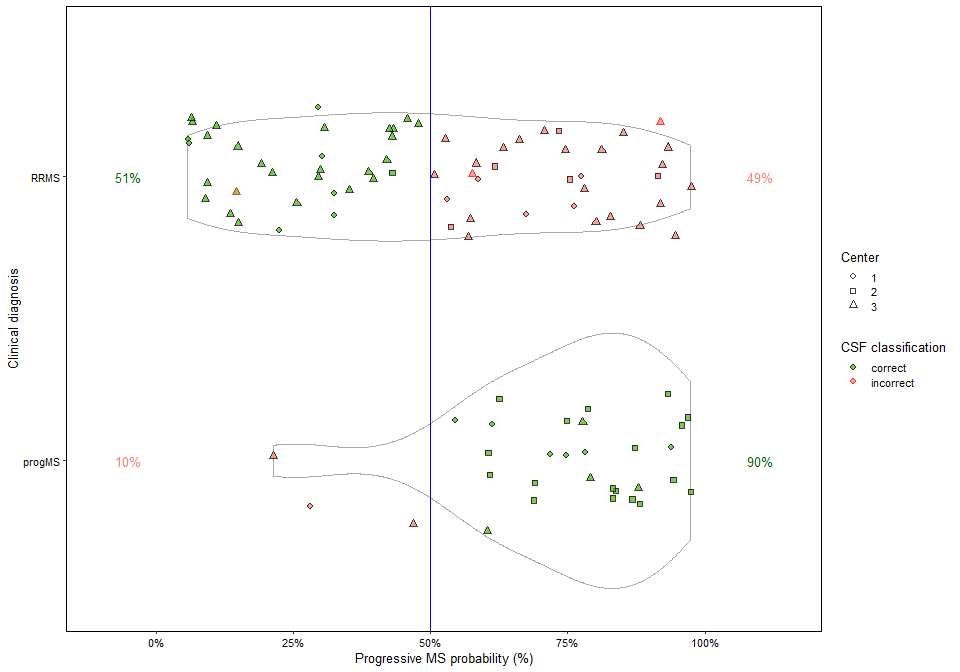


ggsave(p3B, filename = "./prog_MS_probability.png", width = 4.25, height = 2.5, units = "in")

- Figure 3C: Progressive probability by Age as CSF split by progressive/RRMS

#age is age at CSF


#RRMS results (left panel):

df_RR <- dfMSonly %>% dplyr::filter(diagnosis_simple == "RRMS")

lmprogvage_RRMS <- lm(csf_predicted_progressive_ms_probability ~ age, data = df_RR)
summary(lmprogvage_RRMS)

##
## Call:
## lm(formula = csf_predicted_progressive_ms_probability ~ age,
## data = df_RR)
##
## Residuals:
## Min 1Q Median 3Q Max
## -0.40188 -0.17115 -0.03133 0.14120 0.54950
##
## Coefficients:
## Estimate Std. Error t value Pr(>|t|)
## (Intercept) -0.061649 0.107736 -0.572 0.569
## age 0.014534 0.002722 5.339 1.36e-06
##
## Residual standard error: 0.234 on 63 degrees of freedom
## Multiple R-squared: 0.3116, Adjusted R-squared: 0.3006
## F-statistic: 28.51 on 1 and 63 DF, p-value: 1.358e-06

dim(df_RR)

## [1] 65 21

# 2) compute Spearman

corr <- mosaic::cor.test(csf_predicted_progressive_ms_probability ~ age, data = df_RR, method = "spearman")

rho_lbl <- sprintf(
 "rho = %.2f",
 corr$estimate
)
table(df_RR$diagnosis_simple)

##
## progMS RRMS
## 0 65

p3C_Left <- df_RR %>%

 ggplot(aes(x=age,y=csf_predicted_progressive_ms_probability))+
 geom_point(aes(shape=center, fill=diagnosis_simple), color="black",size=1.75,alpha=.75,stroke=.2)+
 scale_shape_manual(values = c(21,22,24),labels = c("1","2","3"),"Center")+
 scale_fill_manual(values = c("darkorange"),breaks = c("RRMS"),"Clinical\ndiagnosis")+
 scale_color_manual(values = c("darkorange"),breaks = c("RRMS"),"Clinical\ndiagnosis")+
 geom_smooth(aes(color=diagnosis_simple, fill=diagnosis_simple),method = "lm", alpha=0.2, linewidth=0.65)+
 scale_y_continuous(limits = c(0,1.2),breaks = c(0,0.25,0.5,0.75,1),labels = c("0","25","50","75","100"))+
 scale_x_continuous(limits = c(17,85),breaks = c(20,40,60,80),labels = c("20","40","60","80"))+
 xlab("Age @ CSF collection")+
 ylab("Progressive MS probability (%)")+
 guides(fill = guide_legend(override.aes = list(shape=21)))+
 geom_hline(yintercept = .5,color="blue",linewidth=.35, linetype="dashed")+
 # your equation
 ggpmisc::stat_poly_eq(
 ggpmisc::use_label(c("R2","P","n"), sep="*\"; \"*"),
 formula = y ~ x,
 size = 3
 ) +

 # plain‐text rho, top‐left with a downward nudge
 annotate(
 "text",
 x = -Inf, # left border
 y = Inf, # top border
 label = rho_lbl,
 hjust = -0.2, # left-align at x = -Inf
 vjust = 4.5, # push it downward ~1 line under the eqn
 size = 3
 ) +
 theme_classic2()+
 theme(
 axis.text.x = element_text(size=8,colour="black",hjust = 0.5, vjust = .5),
 axis.text.y = element_text(size=8,colour="black",hjust = 0.5,vjust = .5),
 axis.title.y = element_text(color="black",size=10,vjust=2),
 axis.title.x = element_text(color="black",size=10),
 legend.title = element_text(size=9, margin = margin(0,0,5,0)),
 # legend.box.background = element_rect(colour = "black",fill="white"),
 legend.justification = c(0, 1),
 legend.text = element_text(size=8, margin = margin(0,0,0,1)),
 legend.key.height = unit(0.15,"in"),

 # legend.spacing = unit(0.25, "lines"),
 axis.line = element_blank(),
 axis.line.x = element_blank(),
 axis.line.y = element_blank(),
 # panel.grid.major.y = element_line(color="gray",size=.25))
 panel.border = element_rect(colour = "black", fill=NA, size=0.5))
p3C_Left


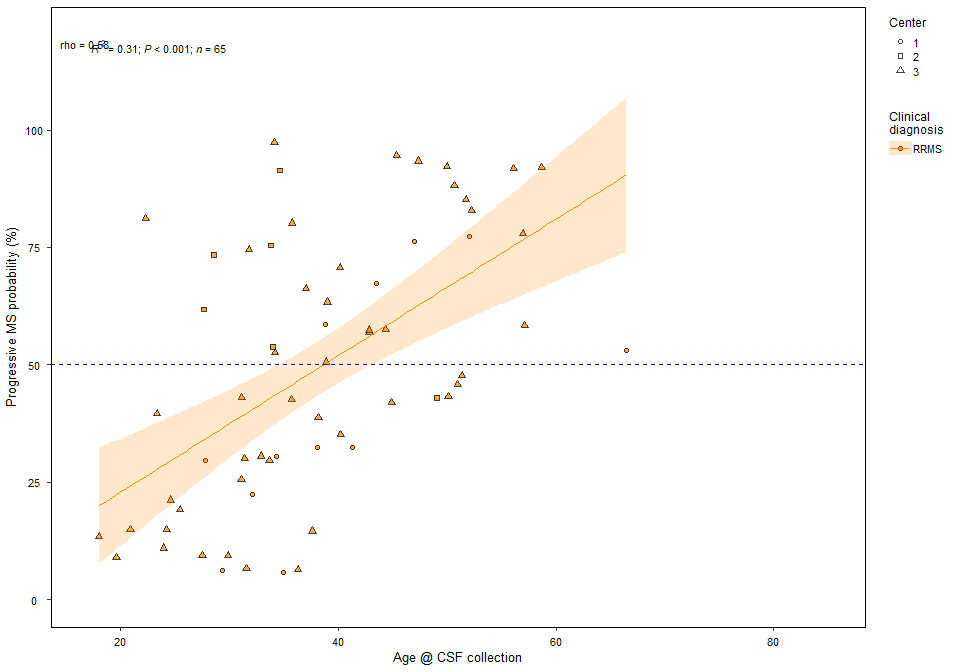


ggsave(plot = p3C_Left,"./progressive_MS_classifier_probability_vs_age_RRMS.png",width = 4,height = 2.5,units = "in")


#progMS results (right panel):

df_prog <- dfMSonly %>% dplyr::filter(diagnosis_simple == "progMS")

lmprogvage_progMS <- lm(csf_predicted_progressive_ms_probability ~ age, data = df_prog)
summary(lmprogvage_progMS)

##
## Call:
## lm(formula = csf_predicted_progressive_ms_probability ~ age,
## data = df_prog)
##
## Residuals:
## Min 1Q Median 3Q Max
## -0.49993 -0.07783 0.02296 0.10612 0.28239
##
## Coefficients:
## Estimate Std. Error t value Pr(>|t|)
## (Intercept) 0.537018 0.169397 3.170 0.00358
## age 0.004020 0.003254 1.235 0.22662
##
## Residual standard error: 0.187 on 29 degrees of freedom
## Multiple R-squared: 0.04999, Adjusted R-squared: 0.01723
## F-statistic: 1.526 on 1 and 29 DF, p-value: 0.2266

dim(df_prog)

## [1] 31 21

# 2) compute Spearman

corr <- mosaic::cor.test(csf_predicted_progressive_ms_probability ~ age, data = df_prog, method = "spearman")

rho_lbl <- sprintf(
 "rho = %.2f",
 corr$estimate
)
table(df_prog$diagnosis_simple)

##
## progMS RRMS
## 31 0

p3C_Right <- df_prog %>%

 ggplot(aes(x=age,y=csf_predicted_progressive_ms_probability))+
 geom_point(aes(shape=center, fill=diagnosis_simple), color="black",size=1.75,alpha=.75,stroke=.2)+
 scale_shape_manual(values = c(21,22,24),labels = c("1","2","3"),"Center")+
 scale_fill_manual(values = c("purple"),breaks = c("progMS"),"Clinical\ndiagnosis")+
 scale_color_manual(values = c("purple"),breaks = c("progMS"),"Clinical\ndiagnosis")+
 geom_smooth(aes(color=diagnosis_simple, fill=diagnosis_simple),method = "lm", alpha=0.2, linewidth=0.65)+
 scale_y_continuous(limits = c(0,1.2),breaks = c(0,0.25,0.5,0.75,1),labels = c("0","25","50","75","100"))+
 scale_x_continuous(limits = c(17,85),breaks = c(20,40,60,80),labels = c("20","40","60","80"))+
 xlab("Age @ CSF collection")+
 ylab("Progressive MS probability (%)")+
 guides(fill = guide_legend(override.aes = list(shape=21)))+
 geom_hline(yintercept = .5,color="blue",linewidth=.35, linetype="dashed")+
 # your equation
 ggpmisc::stat_poly_eq(
 ggpmisc::use_label(c("R2","P","n"), sep="*\"; \"*"),
 formula = y ~ x,
 size = 3
 ) +

 # plain‐text rho, top‐left with a downward nudge
 annotate(
 "text",
 x = -Inf, # left border
 y = Inf, # top border
 label = rho_lbl,
 hjust = -0.2, # left-align at x = -Inf
 vjust = 4.5, # push it downward ~1 line under the eqn
 size = 3
 ) +
 theme_classic2()+
 theme(
 axis.text.x = element_text(size=8,colour="black",hjust = 0.5, vjust = .5),
 axis.text.y = element_text(size=8,colour="black",hjust = 0.5,vjust = .5),
 axis.title.y = element_text(color="black",size=10,vjust=2),
 axis.title.x = element_text(color="black",size=10),
 legend.title = element_text(size=9, margin = margin(0,0,5,0)),
 # legend.box.background = element_rect(colour = "black",fill="white"),
 legend.justification = c(0, 1),
 legend.text = element_text(size=8, margin = margin(0,0,0,1)),
 legend.key.height = unit(0.15,"in"),

 # legend.spacing = unit(0.25, "lines"),
 axis.line = element_blank(),
 axis.line.x = element_blank(),
 axis.line.y = element_blank(),
 # panel.grid.major.y = element_line(color="gray",size=.25))
 panel.border = element_rect(colour = "black", fill=NA, size=0.5))
p3C_Right


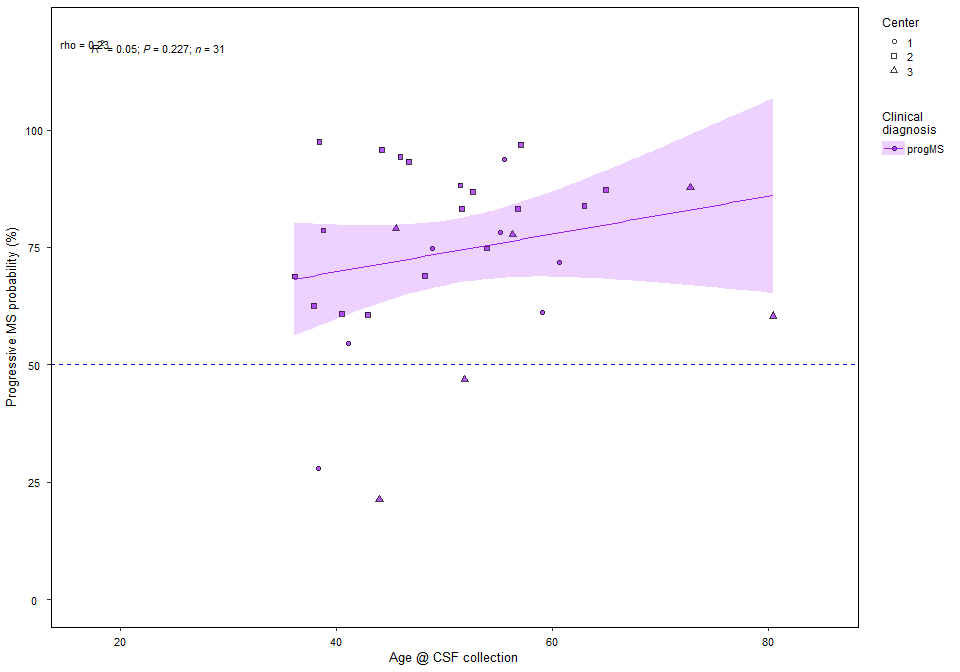


ggsave(plot = p3C_Right,"./progressive_MS_classifier_probability_vs_age_progMS.png",width = 4,height = 2.5,units = "in")

p3C_Left + p3C_Right


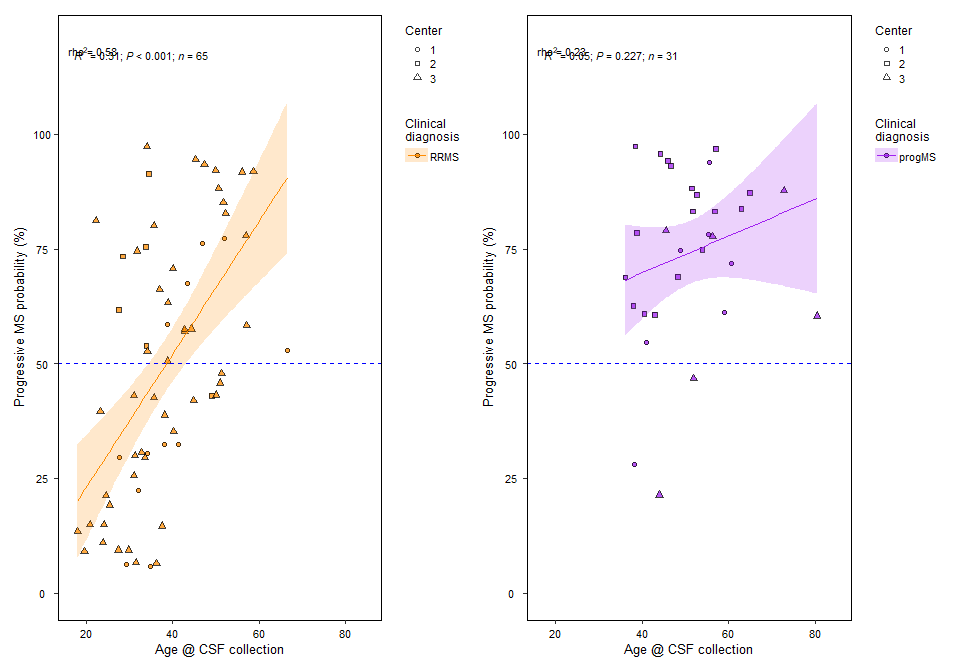


- Figure 3D: Progressive probability by EDSS at followup faceted by progressive/RRMS

#SPINCOMS2 contains EDSS values, join with dfMSonly:

dfCombined <- dfMSonly %>% left_join(SPINCOMS2 %>% dplyr::select(-center),
 c("patient_code" = "patientcode"))

dfCombined <- data.frame(dfCombined) %>% mutate_if(is.character, as.factor)

dfCombinedR <- dfCombined %>% drop_na(measured_edss_at_follow_up) #Remove observations with no EDSS at follow up

#RRMS results (Left panel):

df_RR_EDSS <- dfCombinedR %>% dplyr::filter(diagnosis_simple == "RRMS") %>%
 dplyr::filter(patient_code != c("CCP311", "CCP318")) #Missing age at follow-up


lmprogvEDSS_RRMS <- lm(csf_predicted_progressive_ms_probability ~ measured_edss_at_follow_up, data = df_RR_EDSS)
summary(lmprogvEDSS_RRMS)

##
## Call:
## lm(formula = csf_predicted_progressive_ms_probability ~ measured_edss_at_follow_up,
## data = df_RR_EDSS)
##
## Residuals:
## Min 1Q Median 3Q Max
## -0.62595 -0.18948 -0.00416 0.18589 0.46310
##
## Coefficients:
## Estimate Std. Error t value Pr(>|t|)
## (Intercept) 0.28700 0.05379 5.335 1.53e-06
## measured_edss_at_follow_up 0.06716 0.01491 4.504 3.13e-05
##
## Residual standard error: 0.2436 on 60 degrees of freedom
## Multiple R-squared: 0.2527, Adjusted R-squared: 0.2402
## F-statistic: 20.29 on 1 and 60 DF, p-value: 3.132e-05

dim(df_RR_EDSS)

## [1] 62 28

corr <- mosaic::cor.test(csf_predicted_progressive_ms_probability ~ measured_edss_at_follow_up,
 data = df_RR_EDSS,
 method = "spearman")

table(df_RR_EDSS$diagnosis_simple)

##
## progMS RRMS
## 0 62

rho_lbl <- sprintf(
 "rho = %.2f",
 corr$estimate
)

p3D_Left <- df_RR_EDSS %>%

 ggplot(aes(x=measured_edss_at_follow_up,y=csf_predicted_progressive_ms_probability))+
 geom_point(aes(shape=center, fill=diagnosis_simple), color="black",size=1.75,alpha=.75,stroke=.2)+
 scale_shape_manual(values = c(21,22,24),labels = c("1","2","3"),"Center")+
 scale_fill_manual(values = c("darkorange"),breaks = c("RRMS"),"Clinical\ndiagnosis")+
 scale_color_manual(values = c("darkorange"),breaks = c("RRMS"),"Clinical\ndiagnosis")+
 geom_smooth(aes(color=diagnosis_simple, fill=diagnosis_simple),method = "lm", alpha=0.2, linewidth=0.65)+
 scale_y_continuous(limits = c(0,1.2),breaks = c(0,0.25,0.5,0.75,1),labels = c("0","25","50","75","100"))+
 scale_x_continuous(limits = c(-0.25,9),breaks = c(0,2,4,6,8),labels = c("0","2","4","6","8"))+
 xlab("EDSS @ follow-up")+
 ylab("Progressive MS probability (%)")+
 guides(fill = guide_legend(override.aes = list(shape=21)))+
 geom_hline(yintercept = .5,color="blue",linewidth=.35, linetype="dashed")+
 # your equation
 ggpmisc::stat_poly_eq(
 ggpmisc::use_label(c("R2","P","n"), sep="*\"; \"*"),
 formula = y ~ x,
 size = 3
 ) +

 # plain‐text rho, top‐left with a downward nudge
 annotate(
 "text",
 x = -Inf, # left border
 y = Inf, # top border
 label = rho_lbl,
 hjust = -0.2, # left-align at x = -Inf
 vjust = 4.5, # push it downward ~1 line under the eqn
 size = 3
 ) +
 theme_classic2()+
 theme(
 axis.text.x = element_text(size=8,colour="black",hjust = 0.5, vjust = .5),
 axis.text.y = element_text(size=8,colour="black",hjust = 0.5,vjust = .5),
 axis.title.y = element_text(color="black",size=10,vjust=2),
 axis.title.x = element_text(color="black",size=10),
 legend.title = element_text(size=9, margin = margin(0,0,5,0)),
 # legend.box.background = element_rect(colour = "black",fill="white"),
 legend.justification = c(0, 1),
 legend.text = element_text(size=8, margin = margin(0,0,0,1)),
 legend.key.height = unit(0.15,"in"),

 # legend.spacing = unit(0.25, "lines"),
 axis.line = element_blank(),
 axis.line.x = element_blank(),
 axis.line.y = element_blank(),
 # panel.grid.major.y = element_line(color="gray",size=.25))
 panel.border = element_rect(colour = "black", fill=NA, size=0.5))

p3D_Left


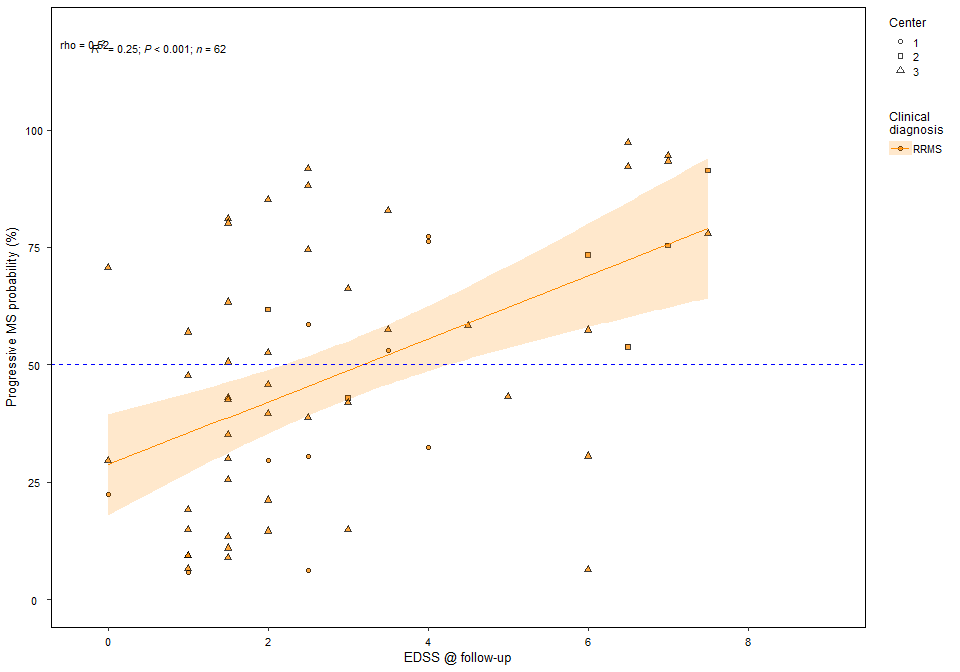


ggsave(plot = p3D_Left,"./progressive_MS_classifier_probability_vs_EDSS_RRMS.png",width = 4,height = 2.5,units = "in")

##
#progMS results (right panel):

df_prog_EDSS <- dfCombinedR %>% dplyr::filter(diagnosis_simple == "progMS")


lmprogvEDSS_progMS <- lm(csf_predicted_progressive_ms_probability ~ measured_edss_at_follow_up, data = df_prog_EDSS)
summary(lmprogvEDSS_progMS)

##
## Call:
## lm(formula = csf_predicted_progressive_ms_probability ~ measured_edss_at_follow_up,
## data = df_prog_EDSS)
##
## Residuals:
## Min 1Q Median 3Q Max
## -0.50331 -0.11131 0.01392 0.17513 0.25748
##
## Coefficients:
## Estimate Std. Error t value Pr(>|t|)
## (Intercept) 0.65854 0.09516 6.920 1.95e-07
## measured_edss_at_follow_up 0.01679 0.01771 0.948 0.351
##
## Residual standard error: 0.1921 on 27 degrees of freedom
## Multiple R-squared: 0.03224, Adjusted R-squared: -0.003604
## F-statistic: 0.8994 on 1 and 27 DF, p-value: 0.3513

dim(df_prog_EDSS)

## [1] 29 28

corr <- mosaic::cor.test(csf_predicted_progressive_ms_probability ~ measured_edss_at_follow_up,
 data = df_prog_EDSS,
 method = "spearman")

table(df_prog_EDSS$diagnosis_simple)

##
## progMS RRMS
## 29 0

rho_lbl <- sprintf(
 "rho = %.2f",
 corr$estimate
)


p3D_Right <- df_prog_EDSS %>%

 ggplot(aes(x=measured_edss_at_follow_up,y=csf_predicted_progressive_ms_probability))+
 geom_point(aes(shape=center, fill=diagnosis_simple), color="black",size=1.75,alpha=.75,stroke=.2)+
 scale_shape_manual(values = c(21,22,24),labels = c("1","2","3"),"Center")+
 scale_fill_manual(values = c("purple"),breaks = c("progMS"),"Clinical\ndiagnosis")+
 scale_color_manual(values = c("purple"),breaks = c("progMS"),"Clinical\ndiagnosis")+
 geom_smooth(aes(color=diagnosis_simple, fill=diagnosis_simple),method = "lm", alpha=0.2, linewidth=0.65)+
 scale_y_continuous(limits = c(0,1.2),breaks = c(0,0.25,0.5,0.75,1),labels = c("0","25","50","75","100"))+
 scale_x_continuous(limits = c(-0.25,9),breaks = c(0,2,4,6,8),labels = c("0","2","4","6","8"))+
 xlab("EDSS @ follow-up")+
 ylab("Progressive MS probability (%)")+
 guides(fill = guide_legend(override.aes = list(shape=21)))+
 geom_hline(yintercept = .5,color="blue",linewidth=.35, linetype="dashed")+
 # your equation
 ggpmisc::stat_poly_eq(
 ggpmisc::use_label(c("R2","P","n"), sep="*\"; \"*"),
 formula = y ~ x,
 size = 3
 ) +

 # plain‐text rho, top‐left with a downward nudge
 annotate(
 "text",
 x = -Inf, # left border
 y = Inf, # top border
 label = rho_lbl,
 hjust = -0.2, # left-align at x = -Inf
 vjust = 4.5, # push it downward ~1 line under the eqn
 size = 3
 ) +
 theme_classic2()+
 theme(
 axis.text.x = element_text(size=8,colour="black",hjust = 0.5, vjust = .5),
 axis.text.y = element_text(size=8,colour="black",hjust = 0.5,vjust = .5),
 axis.title.y = element_text(color="black",size=10,vjust=2),
 axis.title.x = element_text(color="black",size=10),
 legend.title = element_text(size=9, margin = margin(0,0,5,0)),
 # legend.box.background = element_rect(colour = "black",fill="white"),
 legend.justification = c(0, 1),
 legend.text = element_text(size=8, margin = margin(0,0,0,1)),
 legend.key.height = unit(0.15,"in"),

 # legend.spacing = unit(0.25, "lines"),
 axis.line = element_blank(),
 axis.line.x = element_blank(),
 axis.line.y = element_blank(),
 # panel.grid.major.y = element_line(color="gray",size=.25))
 panel.border = element_rect(colour = "black", fill=NA, size=0.5))
p3D_Right


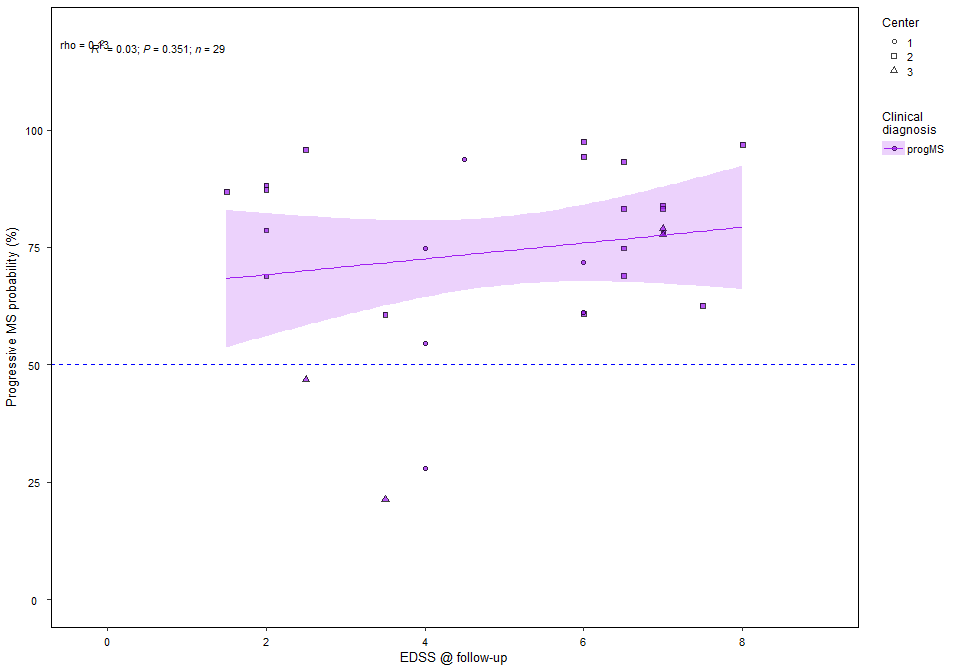


ggsave(plot = p3D_Right,"./progressive_MS_classifier_probability_vs_EDSS_progMS.png",width = 4,height = 2.5,units = "in")


p3D_Left + p3D_Right


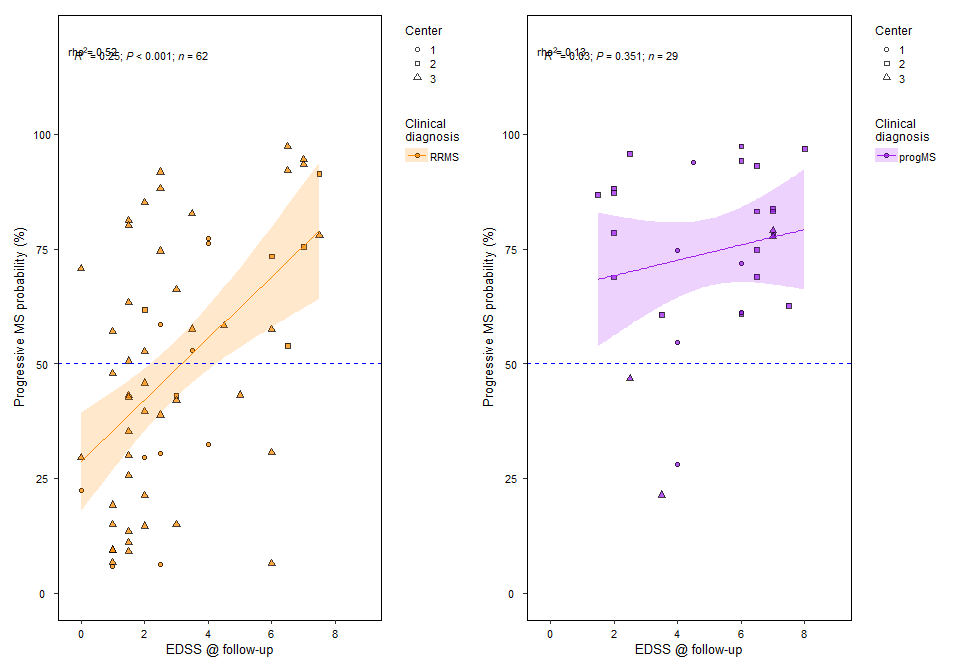


# Figure 4

- Figue 4B: CSF predicted MS-DSS vs Combiwise slope

SPINCOMS4B <- read_excel("SPINCOMS_RAW_DATA_wOCB_FINAL.xlsx",
 sheet = "NDS MSDSS vs Combi slope") %>%
 clean_names()


corr <- mosaic::cor.test(csf_predicted_msdss_at_baseline ~ measured_combi_wise_slope,
 method = "spearman",
 data = SPINCOMS4B)
rho_value <- round(corr$estimate, 2)

p4B <- SPINCOMS4B %>%
 ggplot(aes(x=csf_predicted_msdss_at_baseline,y=measured_combi_wise_slope)) +
 geom_point(shape=21,size=1.5,fill="gray",alpha=0.5,stroke=0.25) +
 ylab("CombiWISE slope")+
 xlab("CSF-predicted baseline MS-DSS")+
 ylim(c(-3,12.1))+
 stat_poly_eq(use_label(c("R2", "P", "n"), sep = "*\"; \"*"),formula = y ~ x ,color="darkgreen", size=2.5) +
 annotate("text", x = 0.5, y = 10,
 label = paste("rho = ", rho_value),
 size = 2.5, hjust = 0, color="darkgreen") + # Adjust size and position as needed
 # stat_poly_eq(label.y = 0.9,color="darkgreen") +

 geom_smooth(method = "lm",formula = y ~ x ,se = TRUE, color="darkgreen", fill="darkgreen",alpha=0.15,linewidth=.5)+
 theme_classic2()+
 theme(
 axis.text.x = element_text(size=7,colour="black",hjust = 0.5, vjust = .5,margin = margin(0,0,0,0)),
 axis.text.y = element_text(size=7,colour="black",hjust = 0.5,vjust = .5,margin = margin(0,0,0,0)),
 axis.title.y = element_text(color="black",size=8,vjust=2,margin = margin(0,0,0,0)),
 axis.title.x = element_text(color="black",size=8,margin = margin(1,0,0,0)),
 legend.title = element_text(size=9),
 # legend.box.background = element_rect(colour = "black",fill="white"),
 # legend.justification = c(0, 1),
 legend.text = element_text(size=8, margin = margin(0,0,0,1)),
 legend.key.height = unit(0.15,"in"),
 legend.position = "none",
 # legend.spacing = unit(0.25, "lines"),
 axis.line = element_blank(),
 axis.line.x = element_blank(),
 axis.line.y = element_blank(),
 # panel.grid.major.y = element_line(color="gray",size=.25))
 panel.border = element_rect(colour = "black", fill=NA, size=0.5))
p4B


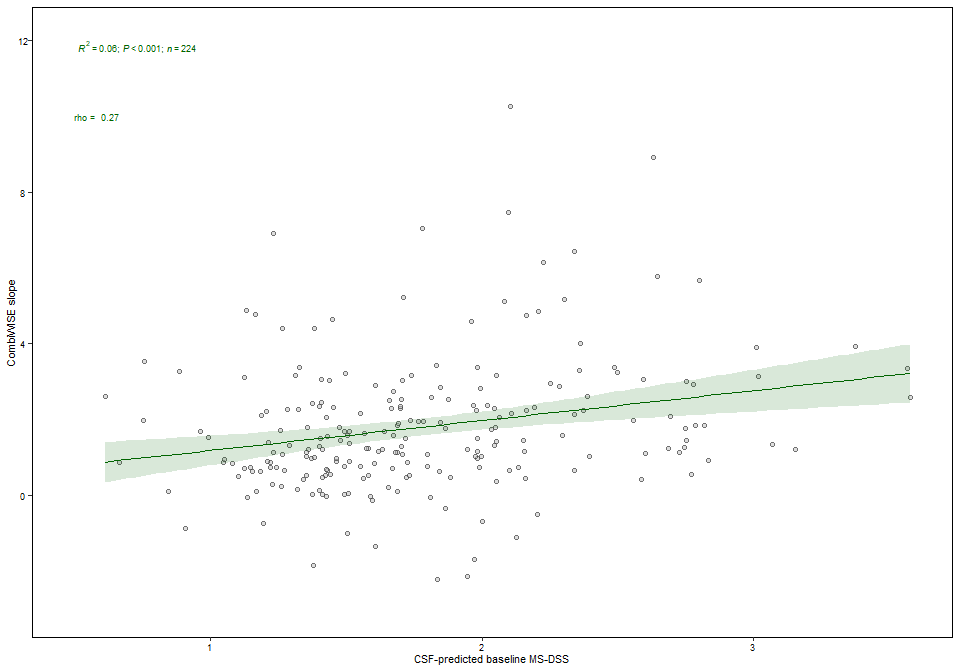


ggsave(plot = p4B,"./nds_csf_baseline_msdss_vs_combi_slope.png",width = 2.3,height = 1.8,units = "in")

- Figure 4C: Combiwise vs EDSS [at follow up]

SPINCOMS4C <- read_excel("SPINCOMS_RAW_DATA_wOCB_FINAL.xlsx",
 sheet = "NDS clinical data", na = "NA") %>%
 clean_names()

SPINCOMS4C <- SPINCOMS4C %>% drop_na(combi_wise, edss)

p4C <- SPINCOMS4C %>%
 ggplot(aes(x=combi_wise,y=edss)) +
 geom_point(shape=21,size=1.5,fill="gray",alpha=0.25,stroke=0.25) +
 ylab("EDSS")+
 xlab("CombiWISE")+
 #ylim(c(0,10))+
 stat_poly_eq(use_label(c("R2", "P", "n"), sep = "*\"; \"*"),formula = y ~ x + I(x^2),color="darkgreen", size=2.5) +
 # stat_poly_eq(label.y = 0.9,color="darkgreen") +
 scale_y_continuous(limits = c(0,10),breaks = c(0,2,4,6,8,10))+
 geom_smooth(method = "lm",formula = y ~ x + I(x^2),se = FALSE, color="darkgreen",fill="darkgreen",alpha=0.25, linewidth=.5)+
 theme_classic2()+
 theme(
 axis.text.x = element_text(size=7,colour="black",hjust = 0.5, vjust = .5,margin = margin(0,0,0,0)),
 axis.text.y = element_text(size=7,colour="black",hjust = 0.5,vjust = .5,margin = margin(0,0,0,0)),
 axis.title.y = element_text(color="black",size=8,vjust=2,margin = margin(0,0,0,0)),
 axis.title.x = element_text(color="black",size=8,margin = margin(1,0,0,0)),
 legend.title = element_text(size=9),
 # legend.box.background = element_rect(colour = "black",fill="white"),
 # legend.justification = c(0, 1),
 legend.text = element_text(size=8, margin = margin(0,0,0,1)),
 legend.key.height = unit(0.15,"in"),
 legend.position = "none",
 # legend.spacing = unit(0.25, "lines"),
 axis.line = element_blank(),
 axis.line.x = element_blank(),
 axis.line.y = element_blank(),
 # panel.grid.major.y = element_line(color="gray",size=.25))
 panel.border = element_rect(colour = "black", fill=NA, size=0.5))
p4C


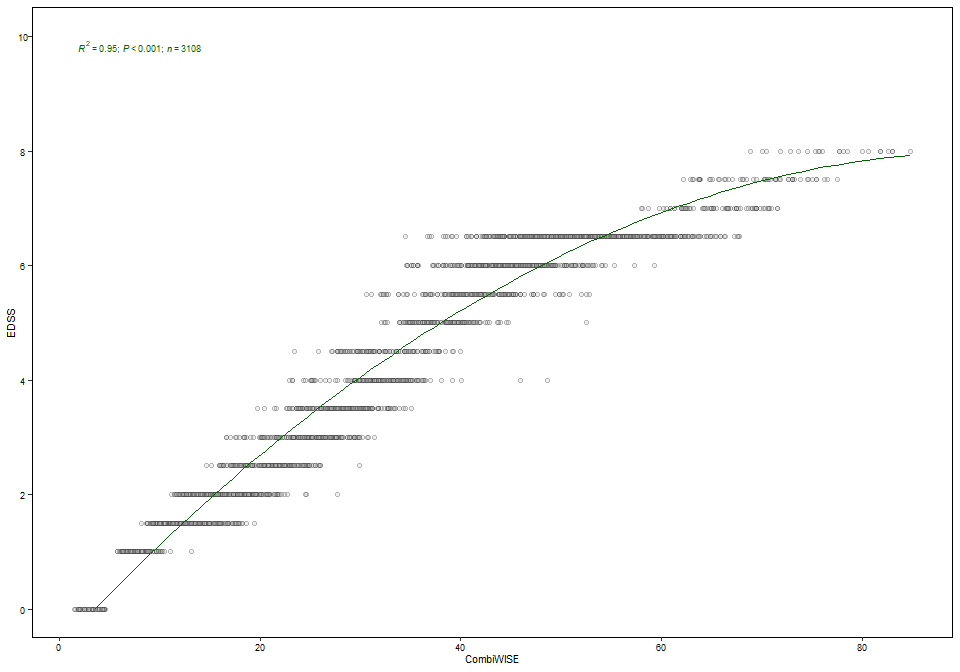


ggsave(plot = p4C,"./nds_edss_vs_combi_quadratic.png",width = 2.3,height = 1.8,units = "in")

- Figure 4D: Post EDSS vs Pre (left) and Predicted Post (right)

dfCombinedR2 <- dfCombined %>% drop_na(edss_at_baseline, measured_edss_at_follow_up)

dim(dfCombinedR2)

## [1] 84 28

Post EDSS based on baseline EDSS (left panel):

corr <- mosaic::cor.test(edss_at_baseline ~ measured_edss_at_follow_up, method = "spearman", data = dfCombinedR2)
rho_value <- round(corr$estimate, 2)


EDSSfollowupmodel1 <- lm(measured_edss_at_follow_up ~ edss_at_baseline, data= dfCombinedR2)
summary(EDSSfollowupmodel1) #R-squared = 0.1567

##
## Call:
## lm(formula = measured_edss_at_follow_up ~ edss_at_baseline, data = dfCombinedR2)
##
## Residuals:
## Min 1Q Median 3Q Max
## -3.7013 -1.6646 -0.4379 1.6654 4.5621
##
## Coefficients:
## Estimate Std. Error t value Pr(>|t|)
## (Intercept) 2.0479 0.4500 4.551 1.83e-05
## edss_at_baseline 0.5933 0.1520 3.904 0.000193
##
## Residual standard error: 2.102 on 82 degrees of freedom
## Multiple R-squared: 0.1567, Adjusted R-squared: 0.1465
## F-statistic: 15.24 on 1 and 82 DF, p-value: 0.0001933

EDSSfollowupmodel1 %>% tbl_regression(intercept = T)

| **Characteristic** | **Beta** | **95% CI** | **p-value** |
| --- | --- | --- | --- |
| (Intercept) | 2.0 | 1.2, 2.9 | <0.001 |
| edss_at_baseline | 0.59 | 0.29, 0.90 | <0.001 |
| Abbreviation: CI = Confidence Interval | | | |

p4D_Left <- dfCombinedR2 %>%
 ggplot(aes(x=edss_at_baseline,y=measured_edss_at_follow_up)) +
 geom_point(size=1.5,aes(fill=diagnosis_simple, shape=center),color="black",alpha=0.5,stroke=0.25) +
 xlab("EDSS @ baseline\n(clinician-based)")+
 ylab("EDSS @ follow-up\n(clinician)")+
 # ylim(c(-11,13))+
 scale_shape_manual(values = c(21,22,24),labels=c("1","2","3"), "Center")+
 scale_fill_manual(values = c("darkorange","purple"),
 breaks = c("RRMS","progMS"),
 "Diagnosis")+
 # geom_abline(slope = 1,intercept = 0,color="black",linewidth=0.25, linetype="dashed")+
 # stat_poly_eq(use_label(c("R2", "P", "n"), sep = "*\"; \"*"),formula = y ~ x ,color="darkgreen",size=2.5) +
 annotate("text", x = -0.2, y = 10.5,
 label = paste0("R\u00B2=", round(summary(EDSSfollowupmodel1)$r.squared, 2),
 "; p=", sprintf("%.2e", summary(EDSSfollowupmodel1)$coefficients[2, 4]),
 "; n=", nobs(EDSSfollowupmodel1)),
 size = 2.5, hjust = 0, color="darkgreen") +
 annotate("text", x = -0.2, y = 9.2,
 label = paste("rho=", rho_value),
 size = 2.5, hjust = 0, color="darkgreen") + # Adjust size and position as needed
 # stat_poly_eq(label.y = 0.9,color="darkgreen") +
 xlim(c(-0.2,11))+
 ylim(c(-0.2,11))+
 # geom_abline(slope = 1,intercept = 0,color="red",linewidth=0.25)+
 geom_smooth(method = "lm",formula = y ~ x ,se = TRUE, color="darkgreen", linewidth=.5,alpha=0.3)+
 guides(fill = guide_legend(override.aes = list(shape=21)))+
 theme_classic2()+
 theme(
 axis.text.x = element_text(size=7,colour="black",hjust = 0.5, vjust = .5),
 axis.text.y = element_text(size=7,colour="black",hjust = 0.5,vjust = .5),
 axis.title.y = element_text(color="#C04F15",size=8,vjust=2),
 axis.title.x = element_text(color="black",size=8),
 legend.title = element_text(size=9),
 # legend.box.background = element_rect(colour = "black",fill="white"),
 # legend.justification = c(0, 1),
 legend.text = element_text(size=8, margin = margin(0,0,0,1)),
 legend.key.height = unit(0.15,"in"),
 legend.position = "none",
 # legend.spacing = unit(0.25, "lines"),
 axis.line = element_blank(),
 axis.line.x = element_blank(),
 axis.line.y = element_blank(),
 # panel.grid.major.y = element_line(color="gray",size=.25))
 panel.border = element_rect(colour = "black", fill=NA, size=0.5))
p4D_Left


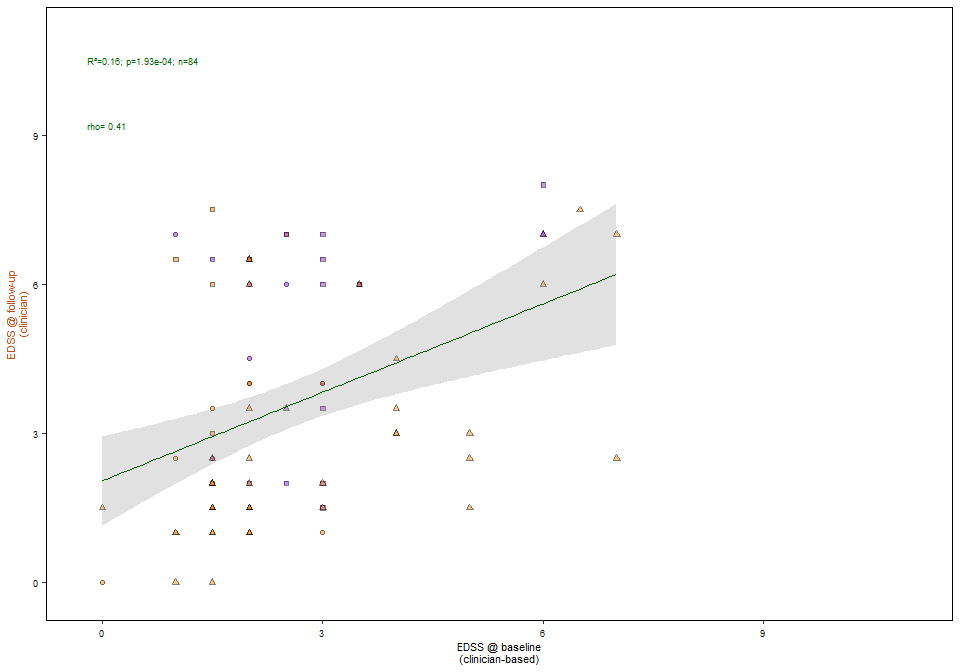


ggsave(plot = p4D_Left,"./edss_base_vs_edss_fu.png",width = 2,height = 1.8,units = "in")

Post EDSS based on CSF predicted post EDSS (right panel):

corr <- mosaic::cor.test(csf_predicted_edss_at_follow_up ~ measured_edss_at_follow_up, method = "spearman", data = dfCombinedR2)
rho_value <- round(corr$estimate, 2)

EDSSfollowupmodel2 <- lm(measured_edss_at_follow_up~csf_predicted_edss_at_follow_up, data = dfCombinedR2)
summary(EDSSfollowupmodel2)

##
## Call:
## lm(formula = measured_edss_at_follow_up ~ csf_predicted_edss_at_follow_up,
## data = dfCombinedR2)
##
## Residuals:
## Min 1Q Median 3Q Max
## -3.8150 -1.6240 -0.3044 1.4662 4.3243
##
## Coefficients:
## Estimate Std. Error t value Pr(>|t|)
## (Intercept) 1.4626 0.5204 2.811 0.00618
## csf_predicted_edss_at_follow_up 0.4852 0.1087 4.464 2.54e-05
##
## Residual standard error: 2.053 on 82 degrees of freedom
## Multiple R-squared: 0.1955, Adjusted R-squared: 0.1857
## F-statistic: 19.93 on 1 and 82 DF, p-value: 2.541e-05

p4D_Right <- dfCombinedR2 %>%
 ggplot(aes(x=csf_predicted_edss_at_follow_up,y=measured_edss_at_follow_up)) +
 geom_point(size=1.5,aes(fill=diagnosis_simple, shape=center),color="black",alpha=0.5,stroke=0.25) +
 xlab("predicted EDSS @ follow-up\n(clinician-based)")+
 ylab("EDSS @ follow-up\n(clinician)")+
 # ylim(c(-11,13))+
 scale_shape_manual(values = c(21,22,24),labels=c("1","2","3"), "Center")+
 scale_fill_manual(values = c("darkorange","purple"),
 breaks = c("RRMS","progMS"),
 "Diagnosis")+
 # geom_abline(slope = 1,intercept = 0,color="black",linewidth=0.25, linetype="dashed")+
 # stat_poly_eq(use_label(c("R2", "P", "n"), sep = "*\"; \"*"),formula = y ~ x ,color="darkgreen",size=2.5) +
 annotate("text", x = -0.2, y = 10.5,
 label = paste0("R\u00B2=", round(summary(EDSSfollowupmodel2)$r.squared, 2),
 "; p=", sprintf("%.2e", summary(EDSSfollowupmodel2)$coefficients[2, 4]),
 "; n=", nobs(EDSSfollowupmodel2)),
 size = 2.5, hjust = 0, color="darkgreen") +
 annotate("text", x = -0.2, y = 9.2,
 label = paste("rho = ", rho_value),
 size = 2.5, hjust = 0, color="darkgreen") + # Adjust size and position as needed
 # stat_poly_eq(label.y = 0.9,color="darkgreen") +
 xlim(c(-0.2,11))+
 ylim(c(-0.2,11))+
 # geom_abline(slope = 1,intercept = 0,color="red",linewidth=0.25)+
 geom_smooth(method = "lm",formula = y ~ x ,se = TRUE, color="darkgreen", linewidth=.5,alpha=0.3)+
 guides(fill = guide_legend(override.aes = list(shape=21)))+
 theme_classic2()+
 theme(
 axis.text.x = element_text(size=7,colour="black",hjust = 0.5, vjust = .5),
 axis.text.y = element_text(size=7,colour="black",hjust = 0.5,vjust = .5),
 axis.title.y = element_text(color="#C04F15",size=8,vjust=2),
 axis.title.x = element_text(color="#7030A0",size=8),
 legend.title = element_text(size=9),
 # legend.box.background = element_rect(colour = "black",fill="white"),
 # legend.justification = c(0, 1),
 legend.text = element_text(size=8, margin = margin(0,0,0,1)),
 legend.key.height = unit(0.15,"in"),
 legend.position = "none",
 # legend.spacing = unit(0.25, "lines"),
 axis.line = element_blank(),
 axis.line.x = element_blank(),
 axis.line.y = element_blank(),
 # panel.grid.major.y = element_line(color="gray",size=.25))
 panel.border = element_rect(colour = "black", fill=NA, size=0.5))
p4D_Right


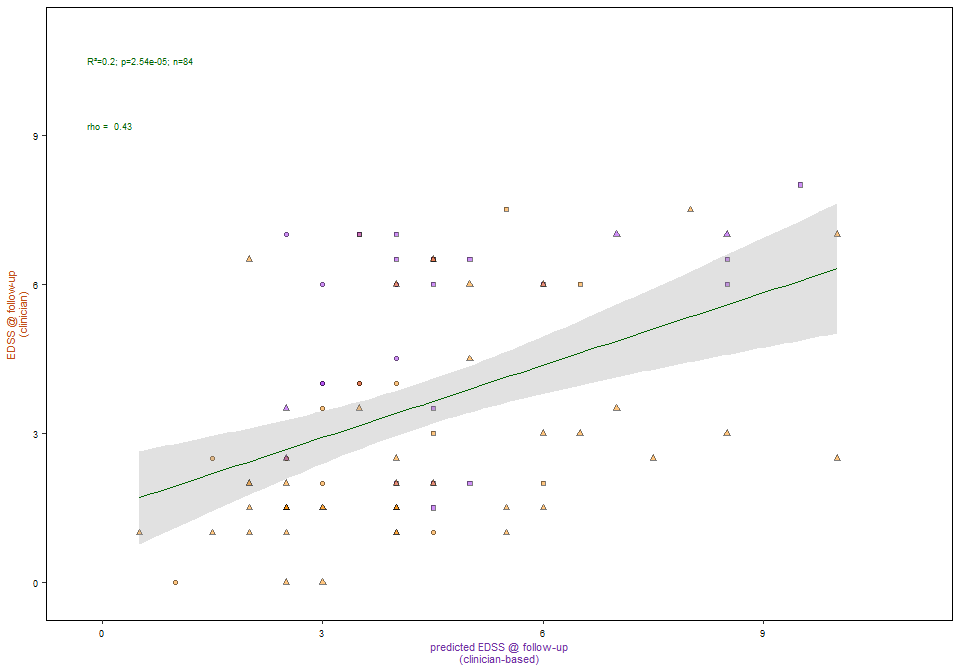


ggsave(plot = p4D_Right,"./edss_clinician_predicted_vs_edss_fu.png",width = 2,height = 1.8,units = "in")

p4D_Left + p4D_Right


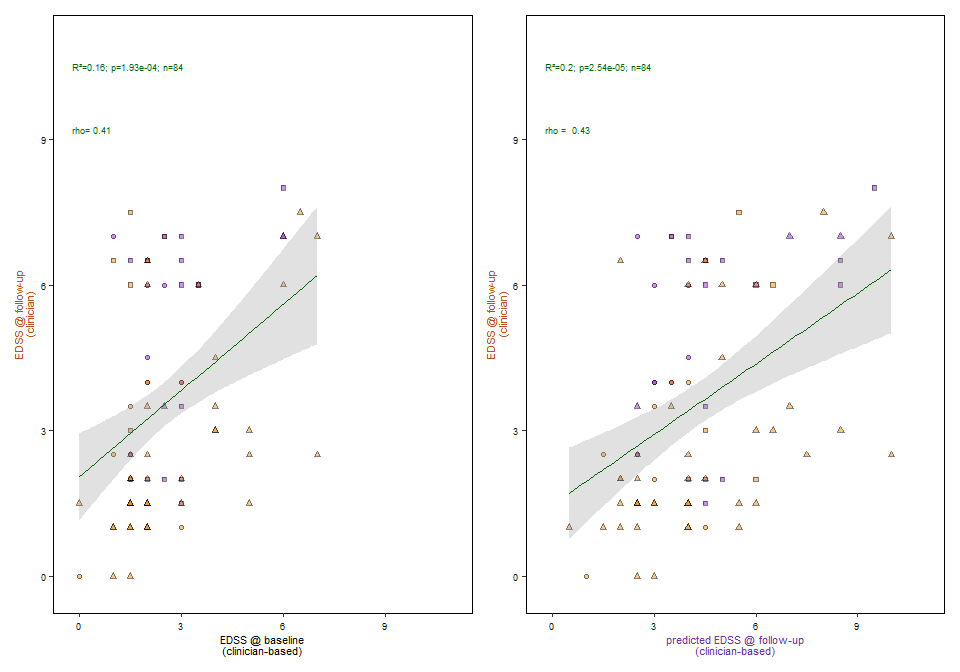


Post EDSS based on baseline EDSS and CSF-predicted change in EDSS

EDSSfollowupmodel_change <- lm(measured_edss_at_follow_up ~ edss_at_baseline + baseline_csf_predicted_edss_change, data= dfCombinedR2)
summary(EDSSfollowupmodel_change) #R-squared = 0.2097

##
## Call:
## lm(formula = measured_edss_at_follow_up ~ edss_at_baseline +
## baseline_csf_predicted_edss_change, data = dfCombinedR2)
##
## Residuals:
## Min 1Q Median 3Q Max
## -3.9768 -1.5039 -0.3416 1.4688 4.3652
##
## Coefficients:
## Estimate Std. Error t value Pr(>|t|)
## (Intercept) 1.3791 0.5239 2.632 0.010149
## edss_at_baseline 0.5473 0.1493 3.664 0.000441
## baseline_csf_predicted_edss_change 0.4425 0.1899 2.331 0.022262
##
## Residual standard error: 2.048 on 81 degrees of freedom
## Multiple R-squared: 0.2097, Adjusted R-squared: 0.1902
## F-statistic: 10.75 on 2 and 81 DF, p-value: 7.242e-05

EDSSfollowupmodel_change %>% tbl_regression(intercept = T)

| **Characteristic** | **Beta** | **95% CI** | **p-value** |
| --- | --- | --- | --- |
| (Intercept) | 1.4 | 0.34, 2.4 | 0.010 |
| edss_at_baseline | 0.55 | 0.25, 0.84 | <0.001 |
| baseline_csf_predicted_edss_change | 0.44 | 0.06, 0.82 | 0.022 |
| Abbreviation: CI = Confidence Interval | | | |

plot(allEffects(EDSSfollowupmodel_change, residuals = T), grid = T) #Not included in figures in paper


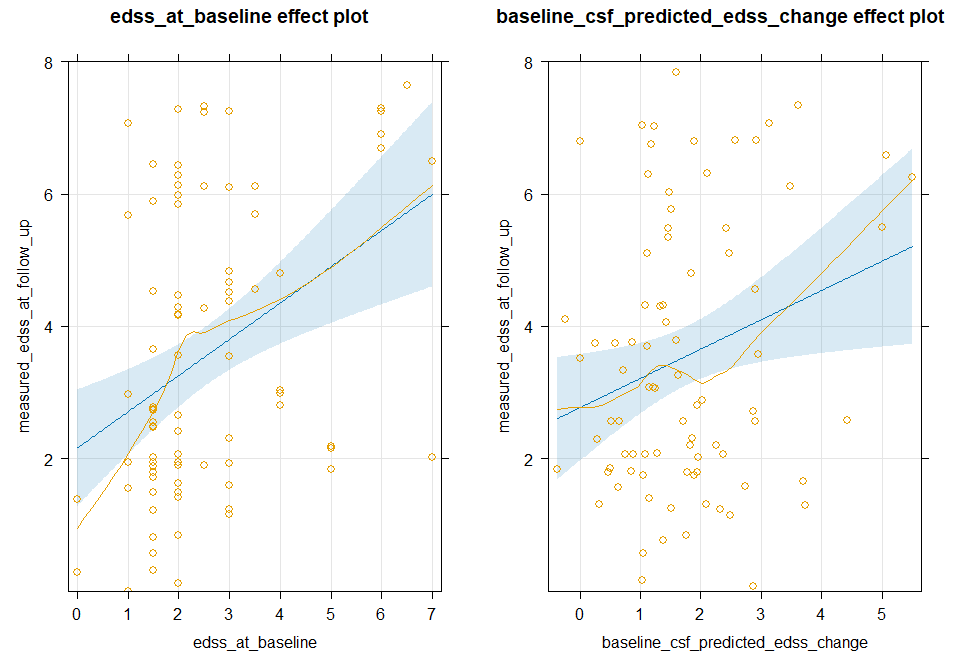


vif(EDSSfollowupmodel_change) #Almost no multicollinearity present

## edss_at_baseline baseline_csf_predicted_edss_change
## 1.017843 1.017843

- Post EDSS ~ Pre EDSS + predicted progressive MS prob + baseline Age

EDSSfollowupmodel4 <- lm(measured_edss_at_follow_up ~ edss_at_baseline + csf_predicted_progressive_ms_probability + age, data= dfCombinedR2)

summary(EDSSfollowupmodel4)

##
## Call:
## lm(formula = measured_edss_at_follow_up ~ edss_at_baseline +
## csf_predicted_progressive_ms_probability + age, data = dfCombinedR2)
##
## Residuals:
## Min 1Q Median 3Q Max
## -3.8526 -1.1139 -0.2409 1.3030 4.2327
##
## Coefficients:
## Estimate Std. Error t value Pr(>|t|)
## (Intercept) -0.47508 0.76297 -0.623 0.53527
## edss_at_baseline 0.32919 0.14085 2.337 0.02193
## csf_predicted_progressive_ms_probability 2.91707 0.92259 3.162 0.00222
## age 0.03850 0.02207 1.744 0.08491
##
## Residual standard error: 1.824 on 80 degrees of freedom
## Multiple R-squared: 0.3808, Adjusted R-squared: 0.3575
## F-statistic: 16.4 on 3 and 80 DF, p-value: 2.142e-08

EDSSfollowupmodel4 %>% tbl_regression(intercept = T)

| **Characteristic** | **Beta** | **95% CI** | **p-value** |
| --- | --- | --- | --- |
| (Intercept) | -0.48 | -2.0, 1.0 | 0.5 |
| edss_at_baseline | 0.33 | 0.05, 0.61 | 0.022 |
| csf_predicted_progressive_ms_probability | 2.9 | 1.1, 4.8 | 0.002 |
| age | 0.04 | -0.01, 0.08 | 0.085 |
| Abbreviation: CI = Confidence Interval | | | |

plot(allEffects(EDSSfollowupmodel4, residuals = T), grid = T) #Not included in figures in paper


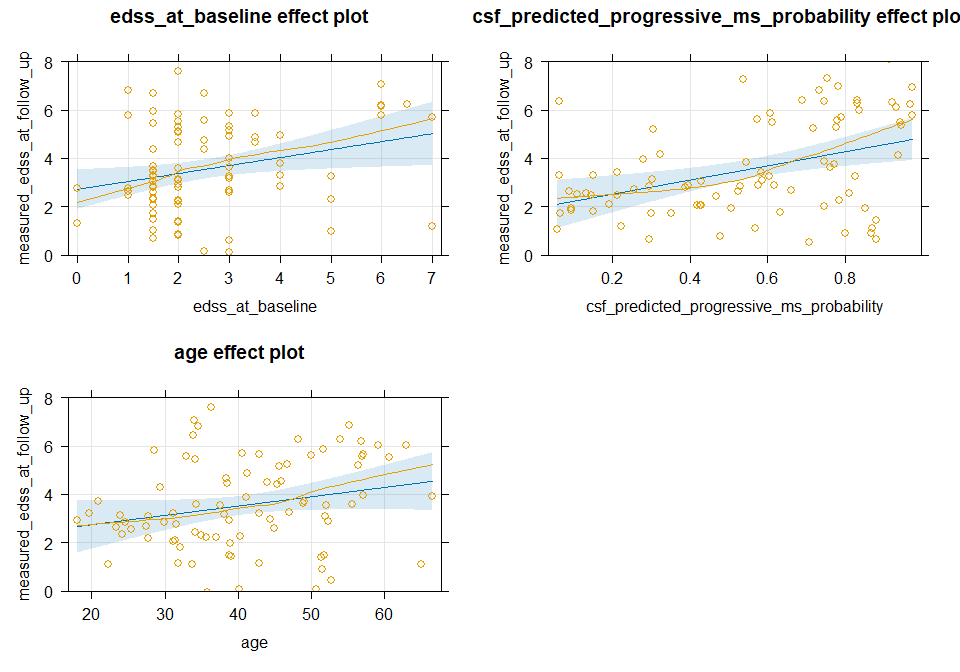


#resid_panel(EDSSfollowupmodel4, "R")

vif(EDSSfollowupmodel4) #Fairly minimal multi-collinearity present

## edss_at_baseline
## 1.141055
## csf_predicted_progressive_ms_probability
## 1.695582
## age
## 1.604256

- Predicted Progressive MS is an important predictor controlled for baseline EDSS and age, t(80) = 3.16, p-value = 0.0022, for the $n$ = 84 subjects with all those variables available. For two otherwise similar subjects but that differ by 1 on predicted probability (so from 0 to 1 on the probability), the estimated mean EDSS at follow-up is 2.92 points higher (95% CI from 1.1 to 4.8), controlled for baseline EDSS and baseline age.
- Note that multi-collinearity is minimal in the model with the highest VIF of 1.7.
- The model has an R-squared of 38.1%, so the combined variables explain a decent but not high amount of variation in the response.
- Figure 4E: MRI-based version: Post EDSS vs Pre (left) and Predicted Post (right)

n = 50 had MRI results

SPINCOMS3 <- read_excel("SPINCOMS_RAW_DATA_wOCB_FINAL.xlsx",
 sheet = "Prognostic data - MRI") %>%
 clean_names() %>%
 mutate(center = factor(center))

SPINCOMS3A <- SPINCOMS3 %>% left_join(dfMSonly %>%
 dplyr::select(patient_code, diagnosis_simple),
 c("patientcode" = "patient_code"))

MRI-based Post EDSS based on baseline EDSS (left panel):

corr <- mosaic::cor.test(mri_predicted_edss_at_baseline ~ measured_edss_at_follow_up, method = "spearman", data = SPINCOMS3A)
rho_value <- round(corr$estimate, 2)


EDSSfollowupmodel1_MRI <- lm(measured_edss_at_follow_up ~ mri_predicted_edss_at_baseline, data= SPINCOMS3A)
summary(EDSSfollowupmodel1_MRI) #R-squared = 0.334

##
## Call:
## lm(formula = measured_edss_at_follow_up ~ mri_predicted_edss_at_baseline,
## data = SPINCOMS3A)
##
## Residuals:
## Min 1Q Median 3Q Max
## -3.4467 -1.3249 0.0897 1.1570 3.8824
##
## Coefficients:
## Estimate Std. Error t value Pr(>|t|)
## (Intercept) 0.8495 0.5447 1.559 0.125
## mri_predicted_edss_at_baseline 0.7073 0.1440 4.910 1.09e-05
##
## Residual standard error: 1.727 on 48 degrees of freedom
## Multiple R-squared: 0.3343, Adjusted R-squared: 0.3205
## F-statistic: 24.11 on 1 and 48 DF, p-value: 1.095e-05

p4E_Left <- SPINCOMS3A %>%
 ggplot(aes(x=mri_predicted_edss_at_baseline,y=measured_edss_at_follow_up)) +
 geom_point(size=1.5,aes(fill=diagnosis_simple, shape=center),color="black",alpha=0.5,stroke=0.25) +
 xlab("EDSS @ baseline\n(MRI-based)")+
 ylab("EDSS @ follow-up\n(clinician)")+
 # ylim(c(-11,13))+
 scale_shape_manual(values = c(21,22,24),labels=c("1","2","3"), "Center")+
 scale_fill_manual(values = c("darkorange","purple"),
 breaks = c("RRMS","progMS"),
 "Diagnosis")+
 # geom_abline(slope = 1,intercept = 0,color="black",linewidth=0.25, linetype="dashed")+
 # stat_poly_eq(use_label(c("R2", "P", "n"), sep = "*\"; \"*"),formula = y ~ x ,color="darkgreen",size=2.5) +
 annotate("text", x = -0.2, y = 10.5,
 label = paste0("R\u00B2=", round(summary(EDSSfollowupmodel1_MRI)$r.squared, 2),
 "; p=", sprintf("%.2e", summary(EDSSfollowupmodel1_MRI)$coefficients[2, 4]),
 "; n=", nobs(EDSSfollowupmodel1_MRI)),
 size = 2.5, hjust = 0, color="darkgreen") +
 annotate("text", x = -0.2, y = 9.2,
 label = paste("rho=", rho_value),
 size = 2.5, hjust = 0, color="darkgreen") + # Adjust size and position as needed
 # stat_poly_eq(label.y = 0.9,color="darkgreen") +
 xlim(c(-0.2,11))+
 ylim(c(-0.2,11))+
 # geom_abline(slope = 1,intercept = 0,color="red",linewidth=0.25)+
 geom_smooth(method = "lm",formula = y ~ x ,se = TRUE, color="darkgreen", linewidth=.5,alpha=0.3)+
 guides(fill = guide_legend(override.aes = list(shape=21)))+
 theme_classic2()+
 theme(
 axis.text.x = element_text(size=7,colour="black",hjust = 0.5, vjust = .5),
 axis.text.y = element_text(size=7,colour="black",hjust = 0.5,vjust = .5),
 axis.title.y = element_text(color="#C04F15",size=8,vjust=2),
 axis.title.x = element_text(color="black",size=8),
 legend.title = element_text(size=9),
 # legend.box.background = element_rect(colour = "black",fill="white"),
 # legend.justification = c(0, 1),
 legend.text = element_text(size=8, margin = margin(0,0,0,1)),
 legend.key.height = unit(0.15,"in"),
 legend.position = "none",
 # legend.spacing = unit(0.25, "lines"),
 axis.line = element_blank(),
 axis.line.x = element_blank(),
 axis.line.y = element_blank(),
 # panel.grid.major.y = element_line(color="gray",size=.25))
 panel.border = element_rect(colour = "black", fill=NA, size=0.5))
p4E_Left


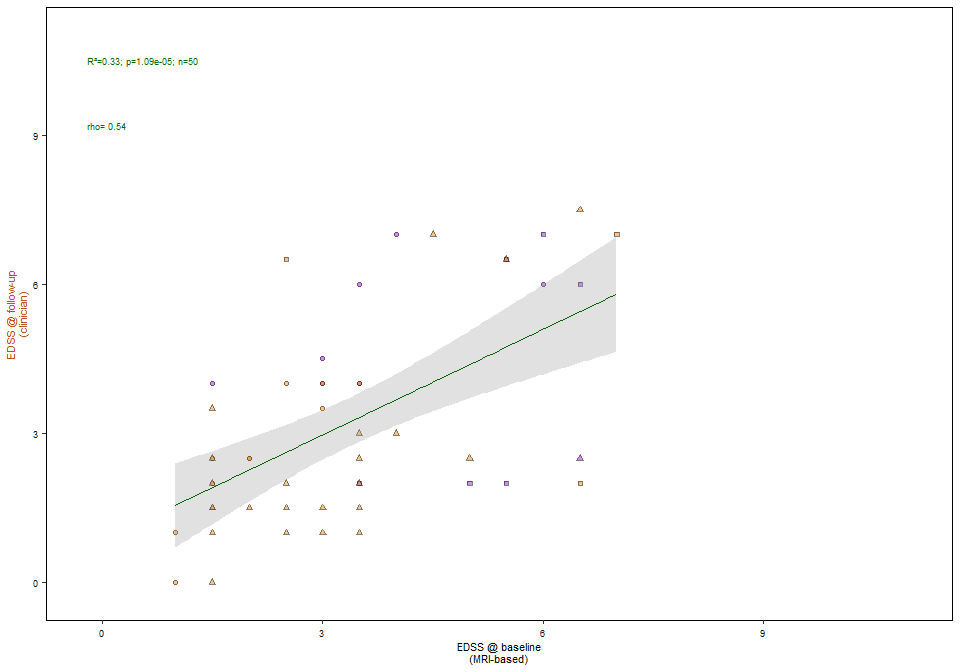


ggsave(plot = p4E_Left,"./edss_base_MRI_vs_edss_fu.png",width = 2,height = 1.8,units = "in")

EDSSfollowupmodel1_MRI_change <- lm(measured_edss_at_follow_up ~ mri_predicted_edss_at_baseline + baseline_csf_predicted_edss_change, data= SPINCOMS3A)
summary(EDSSfollowupmodel1_MRI_change) #R-squared = 0.39

##
## Call:
## lm(formula = measured_edss_at_follow_up ~ mri_predicted_edss_at_baseline +
## baseline_csf_predicted_edss_change, data = SPINCOMS3A)
##
## Residuals:
## Min 1Q Median 3Q Max
## -3.1352 -1.3433 0.1102 1.2158 3.2011
##
## Coefficients:
## Estimate Std. Error t value Pr(>|t|)
## (Intercept) 0.1664 0.6284 0.265 0.7924
## mri_predicted_edss_at_baseline 0.7076 0.1397 5.065 6.75e-06
## baseline_csf_predicted_edss_change 0.5212 0.2597 2.007 0.0505
##
## Residual standard error: 1.675 on 47 degrees of freedom
## Multiple R-squared: 0.3869, Adjusted R-squared: 0.3608
## F-statistic: 14.83 on 2 and 47 DF, p-value: 1.017e-05

plot(allEffects(EDSSfollowupmodel1_MRI_change, residuals = T), grid = T) #Not included in figures in paper


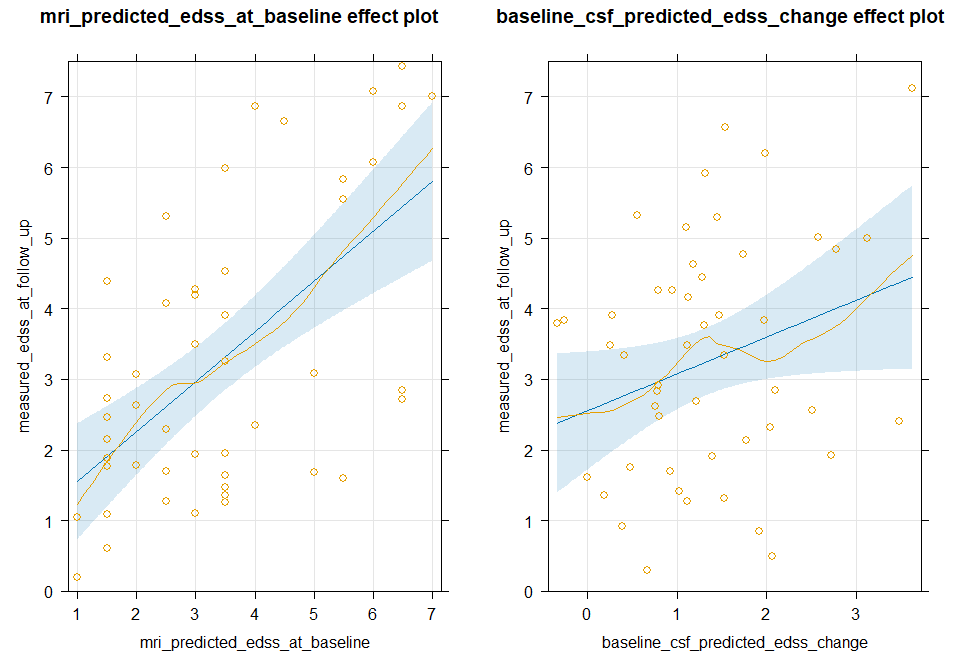


#resid_panel(EDSSfollowupmodel1_MRI_change, "R")

vif(EDSSfollowupmodel1_MRI_change) #No multi-collinearity present

## mri_predicted_edss_at_baseline baseline_csf_predicted_edss_change
## 1.000001 1.000001

MRI-based Post EDSS based on baseline EDSS (right panel): predicted EDSS (MRI) @ follow-up vs EDSS (Clinician) @ follow-up

corr <- mosaic::cor.test(csf_predicted_edss_at_follow_up ~ measured_edss_at_follow_up, method = "spearman", data = SPINCOMS3A)
rho_value <- round(corr$estimate, 2)


EDSSfollowupmodel2_MRI <- lm(measured_edss_at_follow_up~csf_predicted_edss_at_follow_up, data = SPINCOMS3A)

summary(EDSSfollowupmodel2_MRI) #R-squared = 0.379

##
## Call:
## lm(formula = measured_edss_at_follow_up ~ csf_predicted_edss_at_follow_up,
## data = SPINCOMS3A)
##
## Residuals:
## Min 1Q Median 3Q Max
## -3.1122 -1.4470 -0.0303 1.2207 3.2203
##
## Coefficients:
## Estimate Std. Error t value Pr(>|t|)
## (Intercept) 0.1153 0.6242 0.185 0.854
## csf_predicted_edss_at_follow_up 0.6662 0.1232 5.407 1.99e-06
##
## Residual standard error: 1.669 on 48 degrees of freedom
## Multiple R-squared: 0.3785, Adjusted R-squared: 0.3656
## F-statistic: 29.24 on 1 and 48 DF, p-value: 1.99e-06

p4E_Right <- SPINCOMS3A %>%
 ggplot(aes(x=csf_predicted_edss_at_follow_up,y=measured_edss_at_follow_up)) +
 geom_point(size=1.5,aes(fill=diagnosis_simple, shape=center),color="black",alpha=0.5,stroke=0.25) +
 xlab("EDSS @ baseline\n(MRI-based)")+
 ylab("EDSS @ follow-up\n(clinician)")+
 # ylim(c(-11,13))+
 scale_shape_manual(values = c(21,22,24),labels=c("1","2","3"), "Center")+
 scale_fill_manual(values = c("darkorange","purple"),
 breaks = c("RRMS","progMS"),
 "Diagnosis")+
 # geom_abline(slope = 1,intercept = 0,color="black",linewidth=0.25, linetype="dashed")+
 # stat_poly_eq(use_label(c("R2", "P", "n"), sep = "*\"; \"*"),formula = y ~ x ,color="darkgreen",size=2.5) +
 annotate("text", x = -0.2, y = 10.5,
 label = paste0("R\u00B2=", round(summary(EDSSfollowupmodel2_MRI)$r.squared, 2),
 "; p=", sprintf("%.2e", summary(EDSSfollowupmodel2_MRI)$coefficients[2, 4]),
 "; n=", nobs(EDSSfollowupmodel2_MRI)),
 size = 2.5, hjust = 0, color="darkgreen") +
 annotate("text", x = -0.2, y = 9.2,
 label = paste("rho = ", rho_value),
 size = 2.5, hjust = 0, color="darkgreen") + # Adjust size and position as needed
 # stat_poly_eq(label.y = 0.9,color="darkgreen") +
 xlim(c(-0.2,11))+
 ylim(c(-0.2,11))+
 # geom_abline(slope = 1,intercept = 0,color="red",linewidth=0.25)+
 geom_smooth(method = "lm",formula = y ~ x ,se = TRUE, color="darkgreen", linewidth=.5,alpha=0.3)+
 guides(fill = guide_legend(override.aes = list(shape=21)))+
 theme_classic2()+
 theme(
 axis.text.x = element_text(size=7,colour="black",hjust = 0.5, vjust = .5),
 axis.text.y = element_text(size=7,colour="black",hjust = 0.5,vjust = .5),
 axis.title.y = element_text(color="#C04F15",size=8,vjust=2),
 axis.title.x = element_text(color="#7030A0",size=8),
 legend.title = element_text(size=9),
 # legend.box.background = element_rect(colour = "black",fill="white"),
 # legend.justification = c(0, 1),
 legend.text = element_text(size=8, margin = margin(0,0,0,1)),
 legend.key.height = unit(0.15,"in"),
 legend.position = "none",
 # legend.spacing = unit(0.25, "lines"),
 axis.line = element_blank(),
 axis.line.x = element_blank(),
 axis.line.y = element_blank(),
 # panel.grid.major.y = element_line(color="gray",size=.25))
 panel.border = element_rect(colour = "black", fill=NA, size=0.5))
p4E_Right


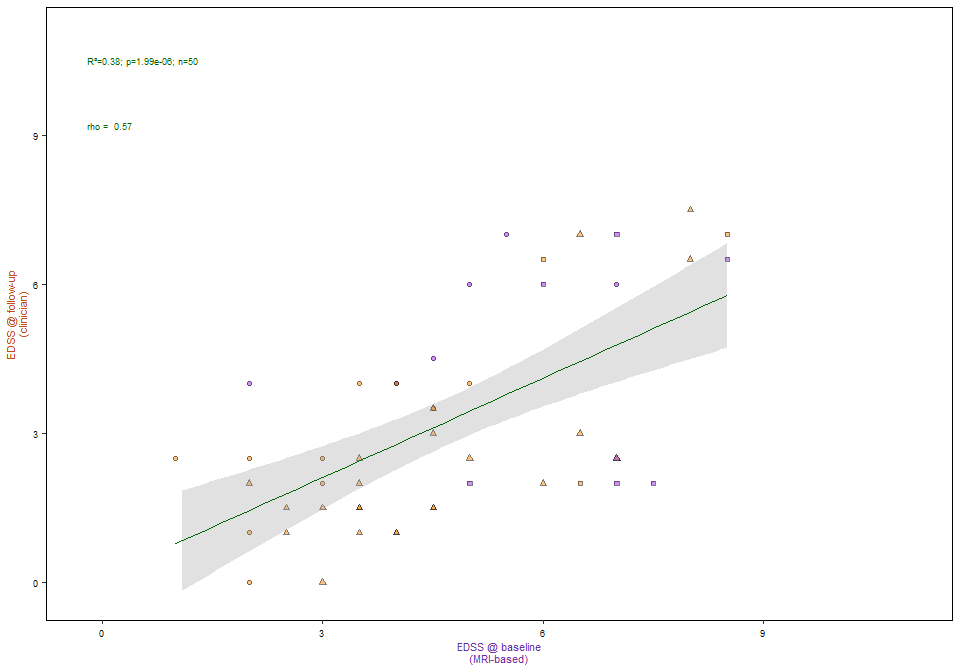


ggsave(plot = p4E_Right,"./edss_mri_predicted_vs_edss_fu.png",width = 2,height = 1.8,units = "in")

p4E_Left + p4E_Right


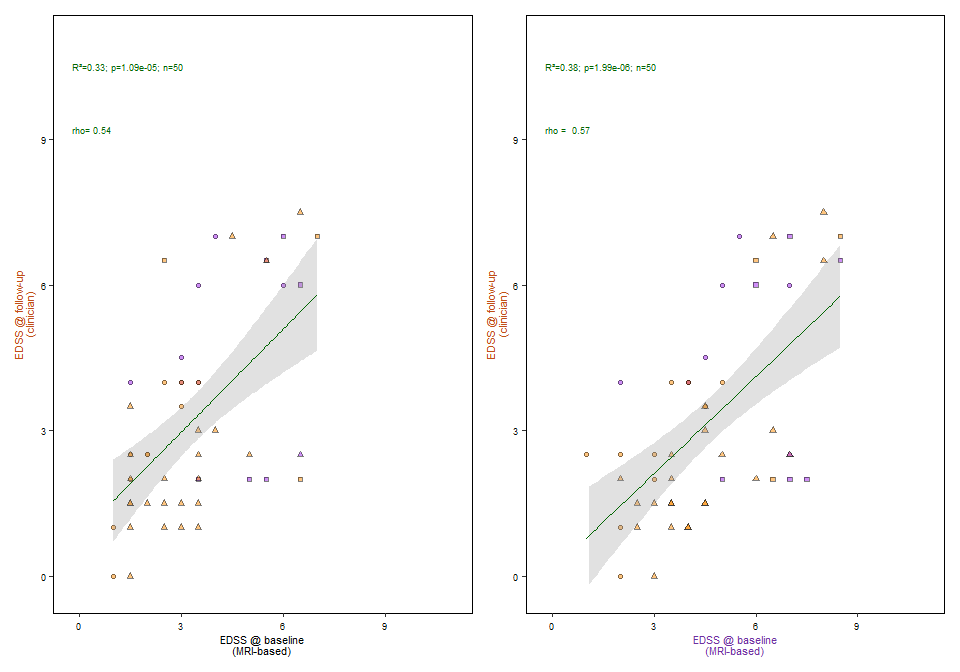


- Figure 4F: rerun in the cohort of clinician-MRI- matching samples

n = 47, subset of data from Figure 4D that were available for MRI-based results

#Merge clinician results from dfCombinedR2 version of edss_at_baseline and drop observations with missing values on initial EDSS:
SPINCOMS3AR <- SPINCOMS3A %>%
 left_join(dfCombinedR2 %>% dplyr::select(patient_code, edss_at_baseline),
 c("patientcode" = "patient_code")) %>%
 drop_na(edss_at_baseline)

dim(SPINCOMS3AR)

## [1] 47 11

Measured EDSS follow up vs EDSS at baseline in subset (left)

corr <- mosaic::cor.test(edss_at_baseline ~ measured_edss_at_follow_up, method = "spearman", data = SPINCOMS3AR)
rho_value <- round(corr$estimate, 2)

EDSSfollowupmodel1_subset <- lm(measured_edss_at_follow_up~edss_at_baseline, data = SPINCOMS3AR)
summary(EDSSfollowupmodel1_subset) #R-squared = 0.0774

##
## Call:
## lm(formula = measured_edss_at_follow_up ~ edss_at_baseline, data = SPINCOMS3AR)
##
## Residuals:
## Min 1Q Median 3Q Max
## -2.7840 -1.5595 -0.5901 0.9099 4.3487
##
## Coefficients:
## Estimate Std. Error t value Pr(>|t|)
## (Intercept) 2.2125 0.5883 3.761 0.000487
## edss_at_baseline 0.4388 0.2258 1.943 0.058285
##
## Residual standard error: 2.019 on 45 degrees of freedom
## Multiple R-squared: 0.0774, Adjusted R-squared: 0.0569
## F-statistic: 3.775 on 1 and 45 DF, p-value: 0.05828

p4F_Left <- SPINCOMS3AR %>%
 ggplot(aes(x=edss_at_baseline,y=measured_edss_at_follow_up)) +
 geom_point(size=1.5,aes(fill=diagnosis_simple, shape=center),color="black",alpha=0.5,stroke=0.25) +
 xlab("EDSS @ baseline\n(clinician-based)")+
 ylab("EDSS @ follow-up\n(clinician)")+
 # ylim(c(-11,13))+
 scale_shape_manual(values = c(21,22,24),labels=c("1","2","3"), "Center")+
 scale_fill_manual(values = c("darkorange","purple"),
 breaks = c("RRMS","progMS"),
 "Diagnosis")+
 # geom_abline(slope = 1,intercept = 0,color="black",linewidth=0.25, linetype="dashed")+
 # stat_poly_eq(use_label(c("R2", "P", "n"), sep = "*\"; \"*"),formula = y ~ x ,color="darkgreen",size=2.5) +
 annotate("text", x = -0.2, y = 10.5,
 label = paste0("R\u00B2=", round(summary(EDSSfollowupmodel1_subset)$r.squared, 2),
 "; p=", sprintf("%.2e", summary(EDSSfollowupmodel1_subset)$coefficients[2, 4]),
 "; n=", nobs(EDSSfollowupmodel1_subset)),
 size = 2.5, hjust = 0, color="darkgreen") +
 annotate("text", x = -0.2, y = 9.2,
 label = paste("rho=", rho_value),
 size = 2.5, hjust = 0, color="darkgreen") + # Adjust size and position as needed
 # stat_poly_eq(label.y = 0.9,color="darkgreen") +
 xlim(c(-0.2,11))+
 ylim(c(-0.2,11))+
 # geom_abline(slope = 1,intercept = 0,color="red",linewidth=0.25)+
 geom_smooth(method = "lm",formula = y ~ x ,se = TRUE, color="darkgreen", linewidth=.5,alpha=0.3)+
 guides(fill = guide_legend(override.aes = list(shape=21)))+
 theme_classic2()+
 theme(
 axis.text.x = element_text(size=7,colour="black",hjust = 0.5, vjust = .5),
 axis.text.y = element_text(size=7,colour="black",hjust = 0.5,vjust = .5),
 axis.title.y = element_text(color="#C04F15",size=8,vjust=2),
 axis.title.x = element_text(color="black",size=8),
 legend.title = element_text(size=9),
 # legend.box.background = element_rect(colour = "black",fill="white"),
 # legend.justification = c(0, 1),
 legend.text = element_text(size=8, margin = margin(0,0,0,1)),
 legend.key.height = unit(0.15,"in"),
 legend.position = "none",
 # legend.spacing = unit(0.25, "lines"),
 axis.line = element_blank(),
 axis.line.x = element_blank(),
 axis.line.y = element_blank(),
 # panel.grid.major.y = element_line(color="gray",size=.25))
 panel.border = element_rect(colour = "black", fill=NA, size=0.5))
p4F_Left


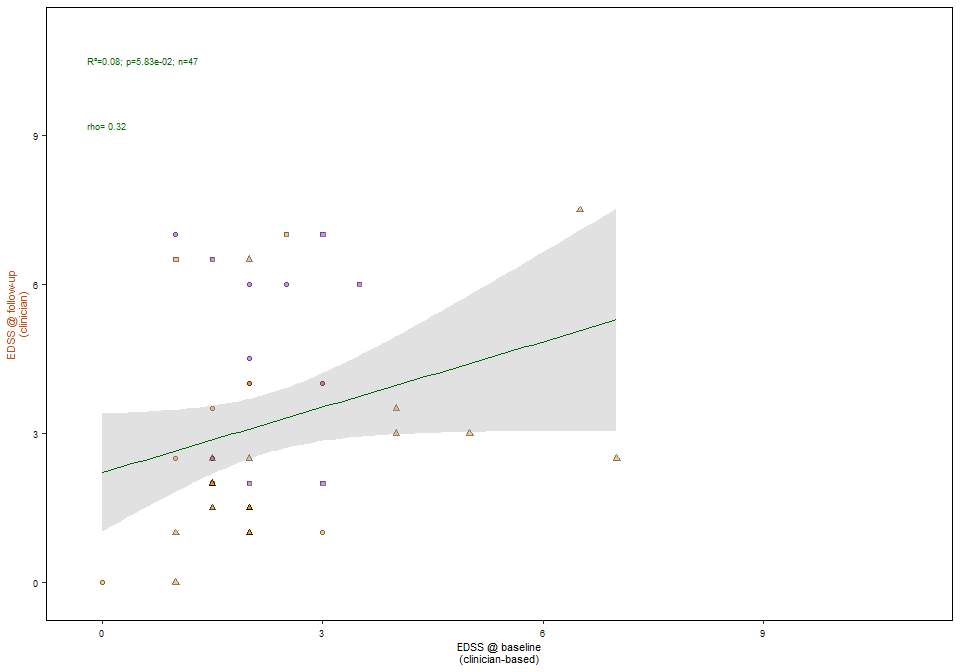


ggsave(plot = p4F_Left,"./edss_base_vs_edss_fu_CLEAN.png",width = 2,height = 1.8,units = "in")

Predicted EDSS (Clinician) @ follow-up vs EDSS (Clinician) @ follow-up (Right) - MRI subjects n= 47 only

#Just the subjects in MRI data and center and diagnosis simple:
MRIonly <- SPINCOMS3AR %>% dplyr::select(patientcode, center, diagnosis_simple)

MRIonly <- MRIonly %>% left_join(dfCombinedR2 %>% dplyr::select(-center, -diagnosis_simple, csf_predicted_edss_at_follow_up, measured_edss_at_follow_up), c("patientcode" = "patient_code"))


corr <- mosaic::cor.test(csf_predicted_edss_at_follow_up ~ measured_edss_at_follow_up, method = "spearman", data = MRIonly)
rho_value <- round(corr$estimate, 2)


EDSSfollowupmodel2_subset <- lm(measured_edss_at_follow_up~csf_predicted_edss_at_follow_up, data = MRIonly)
summary(EDSSfollowupmodel2_subset) #R-squared = 0.07

##
## Call:
## lm(formula = measured_edss_at_follow_up ~ csf_predicted_edss_at_follow_up,
## data = MRIonly)
##
## Residuals:
## Min 1Q Median 3Q Max
## -2.9211 -1.4929 -0.6339 1.0789 4.2225
##
## Coefficients:
## Estimate Std. Error t value Pr(>|t|)
## (Intercept) 2.0597 0.6846 3.009 0.00429
## csf_predicted_edss_at_follow_up 0.2871 0.1552 1.850 0.07085
##
## Residual standard error: 2.026 on 45 degrees of freedom
## Multiple R-squared: 0.0707, Adjusted R-squared: 0.05004
## F-statistic: 3.423 on 1 and 45 DF, p-value: 0.07085

p4F_Right <- MRIonly %>%
 ggplot(aes(x=csf_predicted_edss_at_follow_up,y=measured_edss_at_follow_up)) +
 geom_point(size=1.5,aes(fill=diagnosis_simple, shape=center),color="black",alpha=0.5,stroke=0.25) +
 xlab("predicted EDSS @ follow-up\n(clinician-based)")+
 ylab("EDSS @ follow-up\n(clinician)")+
 # ylim(c(-11,13))+
 scale_shape_manual(values = c(21,22,24),labels=c("1","2","3"), "Center")+
 scale_fill_manual(values = c("darkorange","purple"),
 breaks = c("RRMS","progMS"),
 "Diagnosis")+
 # geom_abline(slope = 1,intercept = 0,color="black",linewidth=0.25, linetype="dashed")+
 # stat_poly_eq(use_label(c("R2", "P", "n"), sep = "*\"; \"*"),formula = y ~ x ,color="darkgreen",size=2.5) +
 annotate("text", x = -0.2, y = 10.5,
 label = paste0("R\u00B2=", round(summary(EDSSfollowupmodel2_subset)$r.squared, 2),
 "; p=", sprintf("%.2e", summary(EDSSfollowupmodel2_subset)$coefficients[2, 4]),
 "; n=", nobs(EDSSfollowupmodel2_subset)),
 size = 2.5, hjust = 0, color="darkgreen") +
 annotate("text", x = -0.2, y = 9.2,
 label = paste("rho = ", rho_value),
 size = 2.5, hjust = 0, color="darkgreen") + # Adjust size and position as needed
 # stat_poly_eq(label.y = 0.9,color="darkgreen") +
 xlim(c(-0.2,11))+
 ylim(c(-0.2,11))+
 # geom_abline(slope = 1,intercept = 0,color="red",linewidth=0.25)+
 geom_smooth(method = "lm",formula = y ~ x ,se = TRUE, color="darkgreen", linewidth=.5,alpha=0.3)+
 guides(fill = guide_legend(override.aes = list(shape=21)))+
 theme_classic2()+
 theme(
 axis.text.x = element_text(size=7,colour="black",hjust = 0.5, vjust = .5),
 axis.text.y = element_text(size=7,colour="black",hjust = 0.5,vjust = .5),
 axis.title.y = element_text(color="#C04F15",size=8,vjust=2),
 axis.title.x = element_text(color="#7030A0",size=8),
 legend.title = element_text(size=9),
 # legend.box.background = element_rect(colour = "black",fill="white"),
 # legend.justification = c(0, 1),
 legend.text = element_text(size=8, margin = margin(0,0,0,1)),
 legend.key.height = unit(0.15,"in"),
 legend.position = "none",
 # legend.spacing = unit(0.25, "lines"),
 axis.line = element_blank(),
 axis.line.x = element_blank(),
 axis.line.y = element_blank(),
 # panel.grid.major.y = element_line(color="gray",size=.25))
 panel.border = element_rect(colour = "black", fill=NA, size=0.5))
p4F_Right


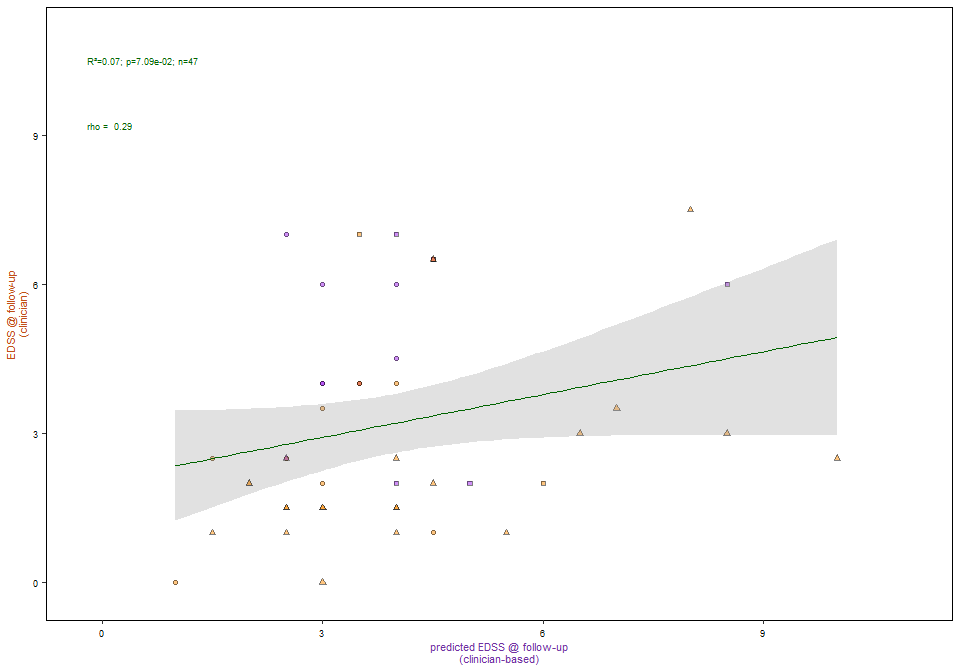


ggsave(plot = p4F_Right ,"./edss_clinician_predicted_vs_edss_fu_CLEAN.png",width = 2,height = 1.8,units = "in")


p4F_Left + p4F_Right


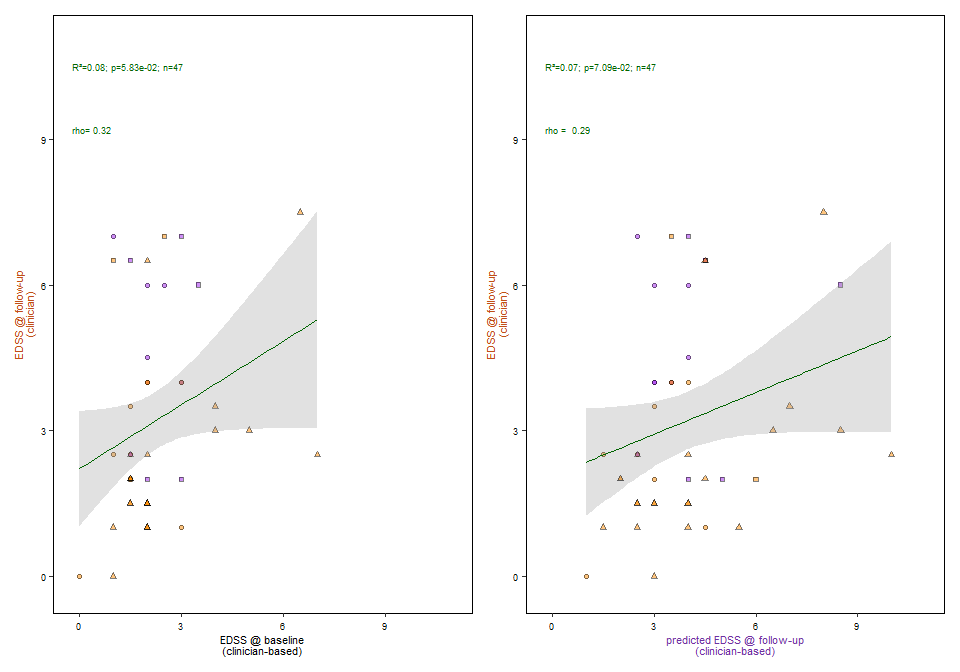


- Figure 4G: rerun in the cohort of clinician-MRI- matching samples

n = 47, subset of data from Figure 4E that were available for MRI-based results

dim(SPINCOMS3AR)

## [1] 47 11

corr <- mosaic::cor.test(mri_predicted_edss_at_baseline ~ measured_edss_at_follow_up, method = "spearman", data = SPINCOMS3AR)
rho_value <- round(corr$estimate, 2)

EDSSfollowupmodel1_MRI_subset <- lm(measured_edss_at_follow_up~mri_predicted_edss_at_baseline, data = SPINCOMS3AR)
summary(EDSSfollowupmodel1_MRI_subset)

##
## Call:
## lm(formula = measured_edss_at_follow_up ~ mri_predicted_edss_at_baseline,
## data = SPINCOMS3AR)
##
## Residuals:
## Min 1Q Median 3Q Max
## -3.4496 -1.2266 0.0832 1.1102 3.8767
##
## Coefficients:
## Estimate Std. Error t value Pr(>|t|)
## (Intercept) 0.8569 0.5359 1.599 0.117
## mri_predicted_edss_at_baseline 0.7066 0.1433 4.931 1.16e-05
##
## Residual standard error: 1.694 on 45 degrees of freedom
## Multiple R-squared: 0.3508, Adjusted R-squared: 0.3364
## F-statistic: 24.32 on 1 and 45 DF, p-value: 1.156e-05

p4G_Left <- SPINCOMS3AR %>%
 ggplot(aes(x=mri_predicted_edss_at_baseline,y=measured_edss_at_follow_up)) +
 geom_point(size=1.5,aes(fill=diagnosis_simple, shape=center),color="black",alpha=0.5,stroke=0.25) +
 xlab("EDSS @ baseline\n(MRI-based)")+
 ylab("EDSS @ follow-up\n(clinician)")+
 # ylim(c(-11,13))+
 scale_shape_manual(values = c(21,22,24),labels=c("1","2","3"), "Center")+
 scale_fill_manual(values = c("darkorange","purple"),
 breaks = c("RRMS","progMS"),
 "Diagnosis")+
 # geom_abline(slope = 1,intercept = 0,color="black",linewidth=0.25, linetype="dashed")+
 # stat_poly_eq(use_label(c("R2", "P", "n"), sep = "*\"; \"*"),formula = y ~ x ,color="darkgreen",size=2.5) +
 annotate("text", x = -0.2, y = 10.5,
 label = paste0("R\u00B2=", round(summary(EDSSfollowupmodel1_MRI_subset)$r.squared, 2),
 "; p=", sprintf("%.2e", summary(EDSSfollowupmodel1_MRI_subset)$coefficients[2, 4]),
 "; n=", nobs(EDSSfollowupmodel1_MRI_subset)),
 size = 2.5, hjust = 0, color="darkgreen") +
 annotate("text", x = -0.2, y = 9.2,
 label = paste("rho=", rho_value),
 size = 2.5, hjust = 0, color="darkgreen") + # Adjust size and position as needed
 # stat_poly_eq(label.y = 0.9,color="darkgreen") +
 xlim(c(-0.2,11))+
 ylim(c(-0.2,11))+
 # geom_abline(slope = 1,intercept = 0,color="red",linewidth=0.25)+
 geom_smooth(method = "lm",formula = y ~ x ,se = TRUE, color="darkgreen", linewidth=.5,alpha=0.3)+
 guides(fill = guide_legend(override.aes = list(shape=21)))+
 theme_classic2()+
 theme(
 axis.text.x = element_text(size=7,colour="black",hjust = 0.5, vjust = .5),
 axis.text.y = element_text(size=7,colour="black",hjust = 0.5,vjust = .5),
 axis.title.y = element_text(color="#C04F15",size=8,vjust=2),
 axis.title.x = element_text(color="black",size=8),
 legend.title = element_text(size=9),
 # legend.box.background = element_rect(colour = "black",fill="white"),
 # legend.justification = c(0, 1),
 legend.text = element_text(size=8, margin = margin(0,0,0,1)),
 legend.key.height = unit(0.15,"in"),
 legend.position = "none",
 # legend.spacing = unit(0.25, "lines"),
 axis.line = element_blank(),
 axis.line.x = element_blank(),
 axis.line.y = element_blank(),
 # panel.grid.major.y = element_line(color="gray",size=.25))
 panel.border = element_rect(colour = "black", fill=NA, size=0.5))
p4G_Left


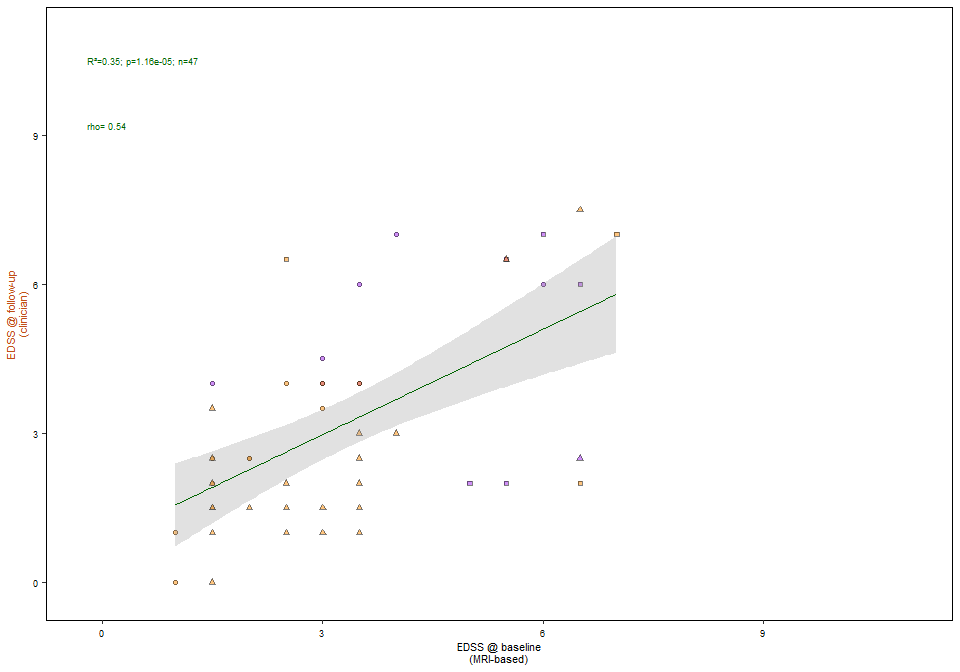


ggsave(plot = p4G_Left,"./edss_base_MRI_vs_edss_fu_CLEAN.png",width = 2,height = 1.8,units = "in")

EDSSfollowupmodel1_MRI_subset_change <- lm(measured_edss_at_follow_up~mri_predicted_edss_at_baseline + baseline_csf_predicted_edss_change, data = SPINCOMS3AR)
summary(EDSSfollowupmodel1_MRI_subset_change) #R-squared = 39%

##
## Call:
## lm(formula = measured_edss_at_follow_up ~ mri_predicted_edss_at_baseline +
## baseline_csf_predicted_edss_change, data = SPINCOMS3AR)
##
## Residuals:
## Min 1Q Median 3Q Max
## -3.0688 -1.2847 0.2878 1.2149 3.2202
##
## Coefficients:
## Estimate Std. Error t value Pr(>|t|)
## (Intercept) 0.2841 0.6256 0.454 0.652
## mri_predicted_edss_at_baseline 0.7035 0.1405 5.009 9.37e-06
## baseline_csf_predicted_edss_change 0.4430 0.2628 1.685 0.099
##
## Residual standard error: 1.66 on 44 degrees of freedom
## Multiple R-squared: 0.3902, Adjusted R-squared: 0.3625
## F-statistic: 14.08 on 2 and 44 DF, p-value: 1.881e-05

plot(allEffects(EDSSfollowupmodel1_MRI_subset_change, residuals = T), grid = T) #Not included in figures in paper


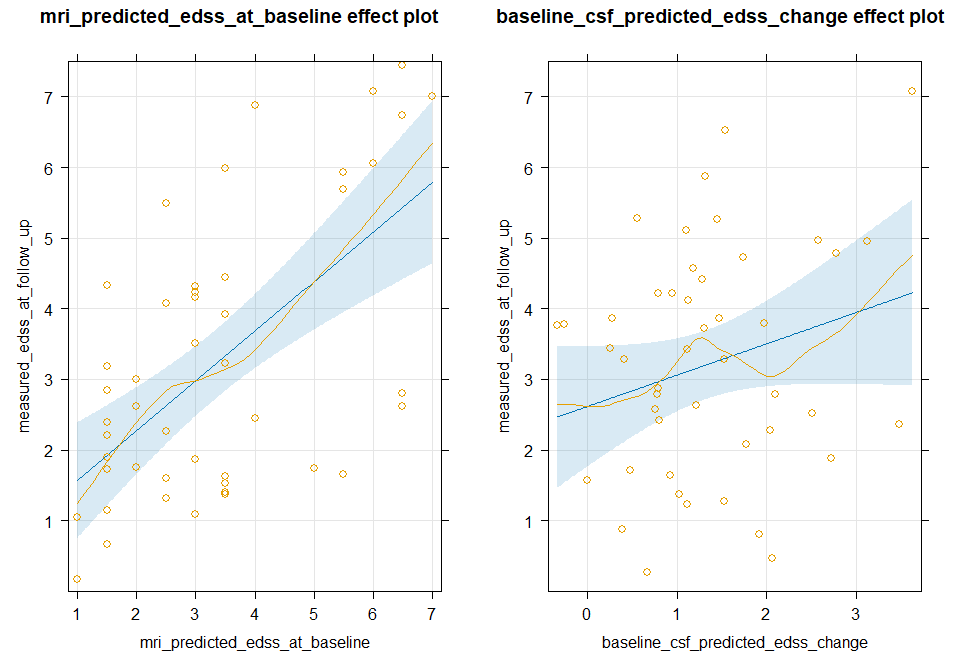


#resid_panel(EDSSfollowupmodel1_MRI_subset_change, "R")

vif(EDSSfollowupmodel1_MRI_subset_change) #No multi-collinearity present

## mri_predicted_edss_at_baseline baseline_csf_predicted_edss_change
## 1.000171 1.000171

predicted EDSS (MRI) @ follow-up vs EDSS (Clinician) @ follow-up (Right)

# Compute Spearman's rho manually
corr <- mosaic::cor.test(csf_predicted_edss_at_follow_up ~ measured_edss_at_follow_up, method = "spearman", data = SPINCOMS3AR)
rho_value <- round(corr$estimate, 2)


EDSSfollowupmodel2_MRI_subset <- lm(measured_edss_at_follow_up~csf_predicted_edss_at_follow_up, data = SPINCOMS3AR)
summary(EDSSfollowupmodel2_MRI_subset)

##
## Call:
## lm(formula = measured_edss_at_follow_up ~ csf_predicted_edss_at_follow_up,
## data = SPINCOMS3AR)
##
## Residuals:
## Min 1Q Median 3Q Max
## -3.0468 -1.4849 0.0316 1.2424 3.2424
##
## Coefficients:
## Estimate Std. Error t value Pr(>|t|)
## (Intercept) 0.2122 0.6233 0.340 0.735
## csf_predicted_edss_at_follow_up 0.6446 0.1238 5.206 4.62e-06
##
## Residual standard error: 1.661 on 45 degrees of freedom
## Multiple R-squared: 0.3759, Adjusted R-squared: 0.362
## F-statistic: 27.1 on 1 and 45 DF, p-value: 4.622e-06

p4G_Right <- SPINCOMS3AR %>%
 ggplot(aes(x=csf_predicted_edss_at_follow_up,y=measured_edss_at_follow_up)) +
 geom_point(size=1.5,aes(fill=diagnosis_simple, shape=center),color="black",alpha=0.5,stroke=0.25) +
 xlab("EDSS @ baseline\n(MRI-based)")+
 ylab("EDSS @ follow-up\n(clinician)")+
 # ylim(c(-11,13))+
 scale_shape_manual(values = c(21,22,24),labels=c("1","2","3"), "Center")+
 scale_fill_manual(values = c("darkorange","purple"),
 breaks = c("RRMS","progMS"),
 "Diagnosis")+
 # geom_abline(slope = 1,intercept = 0,color="black",linewidth=0.25, linetype="dashed")+
 # stat_poly_eq(use_label(c("R2", "P", "n"), sep = "*\"; \"*"),formula = y ~ x ,color="darkgreen",size=2.5) +
 annotate("text", x = -0.2, y = 10.5,
 label = paste0("R\u00B2=", round(summary(EDSSfollowupmodel2_MRI_subset)$r.squared, 2),
 "; p=", sprintf("%.2e", summary(EDSSfollowupmodel2_MRI_subset)$coefficients[2, 4]),
 "; n=", nobs(EDSSfollowupmodel2_MRI_subset)),
 size = 2.5, hjust = 0, color="darkgreen") +
 annotate("text", x = -0.2, y = 9.2,
 label = paste("rho = ", rho_value),
 size = 2.5, hjust = 0, color="darkgreen") + # Adjust size and position as needed
 # stat_poly_eq(label.y = 0.9,color="darkgreen") +
 xlim(c(-0.2,11))+
 ylim(c(-0.2,11))+
 # geom_abline(slope = 1,intercept = 0,color="red",linewidth=0.25)+
 geom_smooth(method = "lm",formula = y ~ x ,se = TRUE, color="darkgreen", linewidth=.5,alpha=0.3)+
 guides(fill = guide_legend(override.aes = list(shape=21)))+
 theme_classic2()+
 theme(
 axis.text.x = element_text(size=7,colour="black",hjust = 0.5, vjust = .5),
 axis.text.y = element_text(size=7,colour="black",hjust = 0.5,vjust = .5),
 axis.title.y = element_text(color="#C04F15",size=8,vjust=2),
 axis.title.x = element_text(color="#7030A0",size=8),
 legend.title = element_text(size=9),
 # legend.box.background = element_rect(colour = "black",fill="white"),
 # legend.justification = c(0, 1),
 legend.text = element_text(size=8, margin = margin(0,0,0,1)),
 legend.key.height = unit(0.15,"in"),
 legend.position = "none",
 # legend.spacing = unit(0.25, "lines"),
 axis.line = element_blank(),
 axis.line.x = element_blank(),
 axis.line.y = element_blank(),
 # panel.grid.major.y = element_line(color="gray",size=.25))
 panel.border = element_rect(colour = "black", fill=NA, size=0.5))
p4G_Right


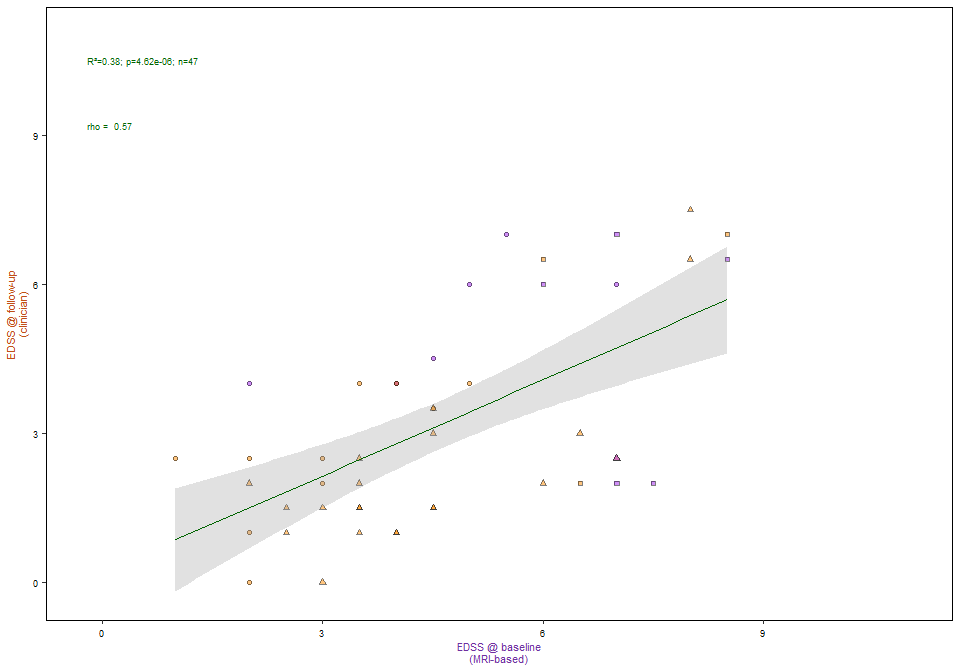


ggsave(plot = p4G_Right,"./edss_mri_predicted_vs_edss_fu_CLEAN.png",width = 2,height = 1.8,units = "in")

p4G_Left + p4G_Right


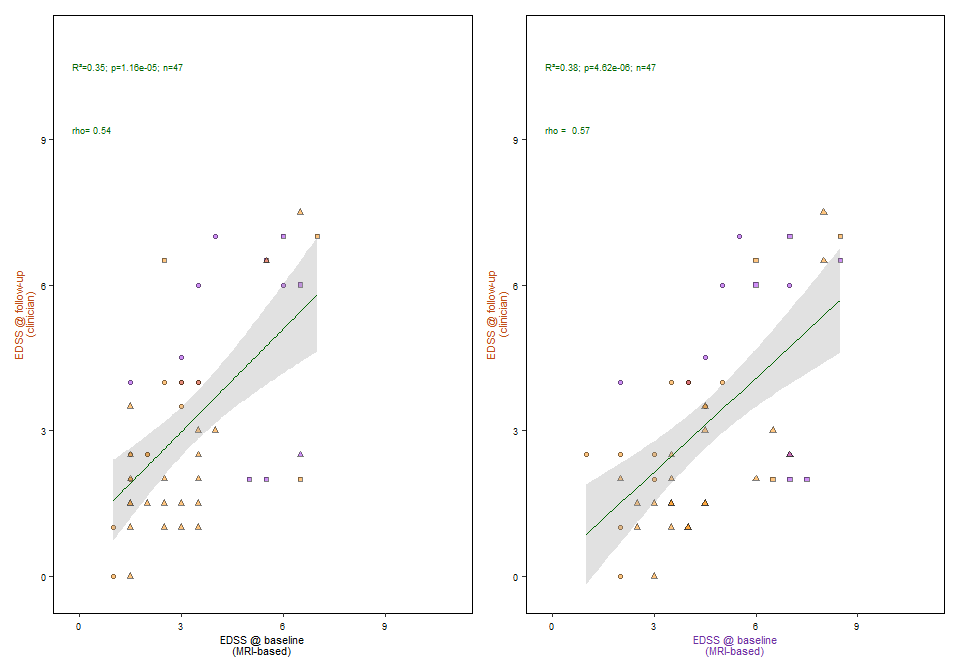


# Table 1 (Raw Material for Final Table in Word)

fortable1 <- df %>% dplyr::select(
 'Center' = center,
 'Age at CSF (years)' = age,
 'Sex' = sex,
 'OCB' = ocb,
 'IgG Index Altered (Y/N)' = is_the_ig_g_index_out_of_range,
 diagnosis_simple,
 ) %>%
 mutate(diagnosis_simple = factor(diagnosis_simple,
 levels = c("RRMS", "progMS", "OIND", "NIND")),
 Sex = factor(Sex))

datasummary_balance(~ diagnosis_simple, data = fortable1,
 title = "Table 1. Summary statistics for full data set.",
 stars = F,
 dinm = T)

Table 1. Summary statistics for full data set.

|  | | RRMS (N=65) | | progMS (N=31) | | OIND (N=30) | | NIND (N=34) | |
| --- | --- | --- | --- | --- | --- | --- | --- | --- | --- |
|  |  | Mean | Std. Dev. | Mean | Std. Dev. | Mean | Std. Dev. | Mean | Std. Dev. |
| Age at CSF (years) |  | 38.1 | 10.7 | 51.0 | 10.5 | 41.3 | 13.2 | 39.7 | 12.5 |
|  |  | N | Pct. | N | Pct. | N | Pct. | N | Pct. |
| Center | 1 | 12 | 18.5 | 7 | 22.6 | 6 | 20.0 | 11 | 32.4 |
|  | 2 | 6 | 9.2 | 18 | 58.1 | 5 | 16.7 | 4 | 11.8 |
|  | 3 | 47 | 72.3 | 6 | 19.4 | 19 | 63.3 | 19 | 55.9 |
| Sex | Female | 44 | 67.7 | 17 | 54.8 | 20 | 66.7 | 28 | 82.4 |
|  | Male | 21 | 32.3 | 14 | 45.2 | 10 | 33.3 | 6 | 17.6 |
| OCB | No | 12 | 18.5 | 6 | 19.4 | 15 | 50.0 | 12 | 35.3 |
|  | Yes | 49 | 75.4 | 23 | 74.2 | 5 | 16.7 | 0 | 0.0 |
|  | NA | 4 | 6.2 | 2 | 6.5 | 10 | 33.3 | 22 | 64.7 |
| IgG Index Altered (Y/N) | No | 25 | 38.5 | 14 | 45.2 | 16 | 53.3 | 12 | 35.3 |
|  | Yes | 36 | 55.4 | 14 | 45.2 | 5 | 16.7 | 0 | 0.0 |
|  | NA | 4 | 6.2 | 3 | 9.7 | 9 | 30.0 | 22 | 64.7 |

datasummary_skim(data = fortable1, by = "diagnosis_simple", type = "numeric", fun_numeric = getOption("modelsummary_fun_numeric",
 default = list(Unique = NUnique, `Missing Pct.` = PercentMissing,
 Mean = Mean, SD = SD, Min = Min, Median = Median,
 Max = Max)))

|  | diagnosis_simple | Unique | Missing Pct. | Mean | SD | Min | Median | Max |
| --- | --- | --- | --- | --- | --- | --- | --- | --- |
| Age at CSF (years) | progMS | 31 | 0 | 51.0 | 10.5 | 36.2 | 51.5 | 80.5 |
|  | RRMS | 65 | 0 | 38.1 | 10.7 | 18.0 | 36.3 | 66.5 |
|  | NIND | 34 | 0 | 39.7 | 12.5 | 17.8 | 36.7 | 68.8 |
|  | OIND | 30 | 0 | 41.3 | 13.2 | 17.1 | 40.0 | 61.6 |

# Summary statistics for MS patients only in Table 1:

fortable1_part2 <- dfCombined %>%
 mutate(FollowupPeriod = age_at_follow_up - age) %>%
 dplyr::select(
 'Center' = center,
 'Age at CSF (years)' = age,
 'Age at follow-up' = age_at_follow_up,
 diagnosis_simple,
 'OCB' = ocb,
 'IgG Index Altered (Y/N)' = is_the_ig_g_index_out_of_range,
 'EDSS (Baseline)' = edss_at_baseline,
 'EDSS (Follow-up)' = measured_edss_at_follow_up,
 'Follow up period' = FollowupPeriod)

datasummary_balance(~ diagnosis_simple, data = fortable1_part2,
 title = "Table 1. Summary statistics for MS patients",
 stars = F,
 dinm = T)

Table 1. Summary statistics for MS patients

|  | | progMS (N=31) | | RRMS (N=65) | |  | |
| --- | --- | --- | --- | --- | --- | --- | --- |
|  |  | Mean | Std. Dev. | Mean | Std. Dev. | Diff. in Means | Std. Error |
| Age at CSF (years) |  | 51.0 | 10.5 | 38.1 | 10.7 | -12.9 | 2.3 |
| Age at follow-up |  | 60.1 | 9.1 | 47.2 | 11.5 | -12.9 | 2.2 |
| EDSS (Baseline) |  | 2.9 | 1.3 | 2.4 | 1.6 | -0.5 | 0.3 |
| EDSS (Follow-up) |  | 5.0 | 2.1 | 3.0 | 2.1 | -2.0 | 0.5 |
| Follow up period |  | 10.8 | 6.2 | 9.5 | 5.3 | -1.3 | 1.3 |
|  |  | N | Pct. | N | Pct. |  |  |
| Center | 1 | 7 | 22.6 | 12 | 18.5 |  |  |
|  | 2 | 18 | 58.1 | 6 | 9.2 |  |  |
|  | 3 | 6 | 19.4 | 47 | 72.3 |  |  |
| OCB | No | 6 | 19.4 | 12 | 18.5 |  |  |
|  | Yes | 23 | 74.2 | 49 | 75.4 |  |  |
| IgG Index Altered (Y/N) | No | 14 | 45.2 | 25 | 38.5 |  |  |
|  | Yes | 14 | 45.2 | 36 | 55.4 |  |  |

datasummary_skim(data = fortable1_part2, by = "diagnosis_simple", type = "numeric", fun_numeric = getOption("modelsummary_fun_numeric",
 default = list(Unique = NUnique, `Missing Pct.` = PercentMissing,
 Mean = Mean, SD = SD, Min = Min, Median = Median,
 Max = Max)))

|  | diagnosis_simple | Unique | Missing Pct. | Mean | SD | Min | Median | Max |
| --- | --- | --- | --- | --- | --- | --- | --- | --- |
| Age at CSF (years) | progMS | 31 | 0 | 51.0 | 10.5 | 36.2 | 51.5 | 80.5 |
|  | RRMS | 65 | 0 | 38.1 | 10.7 | 18.0 | 36.3 | 66.5 |
| Age at follow-up | progMS | 29 | 6 | 60.1 | 9.1 | 43.2 | 62.0 | 74.7 |
|  | RRMS | 59 | 5 | 47.2 | 11.5 | 25.4 | 46.6 | 74.2 |
| EDSS (Baseline) | progMS | 8 | 16 | 2.9 | 1.3 | 1.0 | 2.8 | 6.0 |
|  | RRMS | 13 | 11 | 2.4 | 1.6 | 0.0 | 2.0 | 7.0 |
| EDSS (Follow-up) | progMS | 12 | 6 | 5.0 | 2.1 | 1.5 | 6.0 | 8.0 |
|  | RRMS | 15 | 5 | 3.0 | 2.1 | 0.0 | 2.2 | 7.5 |
| Follow up period | progMS | 30 | 6 | 10.8 | 6.2 | 2.9 | 8.8 | 26.8 |
|  | RRMS | 63 | 5 | 9.5 | 5.3 | 0.7 | 8.9 | 33.7 |

favstats(`EDSS (Baseline)` ~ diagnosis_simple, data = fortable1_part2)

## diagnosis_simple min Q1 median Q3 max mean sd n missing
## 1 progMS 1 2.0 2.75 3 6 2.884615 1.306198 26 5
## 2 RRMS 0 1.5 2.00 3 7 2.396552 1.591531 58 7

favstats(`EDSS (Follow-up)` ~ diagnosis_simple, data = fortable1_part2)

## diagnosis_simple min Q1 median Q3 max mean sd n missing
## 1 progMS 1.5 3.5 6.00 6.5 8.0 4.982759 2.050622 29 2
## 2 RRMS 0.0 1.5 2.25 4.0 7.5 2.951613 2.091571 62 3
